# Citrate Transporter NaCT and Enamel Mineralization: The *Slc13a5*^R337*^ Mouse Model

**DOI:** 10.3390/ijms27167129

**Published:** 2026-08-09

**Authors:** Charles E. Smith, James P. Simmer, Tian Liang, Yuanyuan Hu, Olamide Animasahun, Ajay Shankaran, Deepak Nagrath, Hong Zhang, Ravi Prakash, Chuhua Zhang, Lauren E. Surface, Jie Ren Gerald Har, Julian Zora, Hui Li, Jan Ching-Chun Hu

**Affiliations:** 1 Department of Anatomy & Cell Biology, Faculty of Medicine & Health Sciences, McGill University, Montreal, QC H3A 0C7, Canada; charles.smith@mcgill.ca; 2 Department of Biologic and Materials Sciences & Prosthodontics, University of Michigan School of Dentistry, 1011 N University Ave., Ann Arbor, MI 48109, USA; jsimmer@umich.edu (J.P.S.); zhanghon@umich.edu (H.Z.); rprak@umich.edu (R.P.); chuhuaz@umich.edu (C.Z.); lsurface@umich.edu (L.E.S.); jierenha@umich.edu (J.R.G.H.); lih2051@gmail.com (H.L.); 3 Department of Orthodontic and Pediatric Dentistry, University of Michigan School of Dentistry, 1011 N University Ave., Ann Arbor, MI 48109, USA; tianl@umich.edu (T.L.); yyhu@umich.edu (Y.H.); 4 Biomedical Engineering, College of Engineering, University of Michigan, Ann Arbor, MI 48109, USA; aolamide@umich.edu (O.A.); ajaysh@umich.edu (A.S.); dnagrath@umich.edu (D.N.); 5 Department of Chemical Engineering, College of Engineering, University of Michigan, Ann Arbor, MI 48109, USA; 6 Biomolecular Science, College of Literature, Science, and the Arts, University of Michigan, Ann Arbor, MI 48109, USA; jjzora@umich.edu

**Keywords:** enamel hypoplasia, amelogenesis, TCA metabolites, *Ank* expression, *Slc25a1* expression

## Abstract

Solute Carrier Family 13 Member 5 (*SLC13A5*) encodes the sodium-dependent citrate cotransporter NaCT, which mediates citrate transport across cell membranes. Pathogenic variants in *SLC13A5* cause developmental and epileptic encephalopathy 25 with amelogenesis imperfecta, DEE25; OMIM #615905, a debilitating autosomal recessive disorder. To better define the role of NaCT in ameloblast function and enamel mineralization, we used CRISPR/Cas9 genome editing to generate *Slc13a5*^R337*^ knock-in mice that terminate NaCT translation at the Arg337 codon, which is homologous to the human *SLC13A5*^R333*^ variant associated with DEE25. We compared enamel phenotypes among wild-type, *Slc13a5*^+/+^; heterozygous, *Slc13a5*^+/R337*^; and homozygous, *Slc13a5*^R337*/R337*^ mice using light microscopy, in situ hybridization, immunohistochemistry, backscattered scanning electron microscopy (bSEM); and focused ion beam–scanning electron microscopy (FIB-SEM) with quantitative imaging of organelles and matrix. Citrate bioassays were performed on serum, long bones, such as the femur and tibia, and developing mouse first molars, including enamel organ epithelium, mineralized tooth matrix, and pulp mesenchyme, to assess citrate levels during the presecretory, secretory, and maturation stages of enamel formation. In addition, first molars collected at postnatal days 0, 3, 5, and 12 were analyzed to characterize glycolytic and TCA cycle-related metabolic signatures. Homozygous *Slc13a5*^R337*/R337*^ mice exhibited severe defects during the secretory and maturation stages of amelogenesis. Most notably, *Slc13a5*^R337*/R337*^ ameloblasts failed to develop a Tomes’ process, detached from the enamel matrix surface, and produced a thin, poorly mineralized crust on the dentin surface rather than organized enamel ribbons. Despite the absence of normal enamel deposition, ameloblasts initially appeared viable and did not become dysplastic until the late secretory stage. Cellular and subcellular analyses revealed increased secondary lysosomes and intracellular accumulation of enamel matrix proteins, consistent with impaired matrix processing or secretion. Citrate concentrations were elevated in serum and long bones at both 7 and 35 weeks of age. Citrate was elevated in secretory-stage *Slc13a5*^R337*/R337*^ molars at days 0 and 3, the enamel organ epithelium (including ameloblasts), the pulp mesenchyme (including odontoblasts), and mineralizing dentin and enamel matrices. These levels gradually declined at day 5 and into the enamel maturation stage (day 12). GC-MS-based analysis of central carbon metabolites revealed increased intracellular accumulation of citrate, malate, and pyruvate, suggesting altered energy metabolism and reduced metabolic efficiency in *Slc13a5*^R337*/R337*^ mice. Together, these findings indicate that loss of NaCT function in the ameloblasts causes citrate accumulation, which impairs hydroxyapatite formation. Consequently, only a thin, structurally defective mineral crust forms on the dentin surface, while mineral nodules develop ectopically within the maturation-stage enamel organ epithelium. We conclude that regulating citrate concentration is essential for proper appositional growth of enamel.

## 1. Introduction

Mutations in *SLC13A5*, which encodes the sodium-dependent citrate transporter NaCT, cause developmental epileptic encephalopathy-25, DEE-25, MIM #615905 [1,2,3], a rare but devastating disorder characterized by intractable neonatal-onset epilepsy, motor and cognitive abnormalities, and severe hypoplastic amelogenesis imperfecta [4]. Amelogenesis imperfecta (AI) is a clinically and genetically heterogeneous condition. Depending on the population studied, its prevalence ranges from 1 in 700 to 1 in 14,000. More than 115 genes and disorders have been associated with hereditary enamel defects [5]. DEE25 is a prime example of AI accompanied by neurologic and musculoskeletal systemic defects. Complete absence of dental enamel has been reported in patients with DEE-25 [1,6] and in *Slc13a5* knockout mice [7]. A similar arrest of enamel formation has been observed in mice lacking three enamel-specific matrix or membrane-associated proteins: ameloblastin [8], enamelin [9], and acid phosphatase 4 [10]. Ameloblastin and enamelin are secreted enamel matrix proteins critical for enamel ribbon elongation [11], while acid phosphatase 4 primarily functions to process enamel matrix proteins and presumably provides the phosphate for tissue mineralization [10]. However, in those models, the phenotype is restricted to teeth. NaCT, therefore, stands out as a membrane protein essential for both amelogenesis and nervous system function. Our study aims to elucidate the mechanism of citrate-mediated amelogenesis to advance our understanding of DEE-25-associated tooth defects.

Citrate is intimately linked to metabolic health, mediating biological processes essential for energy production, lipogenesis, inhibition of glycolysis, blood clotting, and pH regulation [12]. Citrate is also critical for the formation and maintenance of mineralized tissues, including bones and teeth. Citrate accounts for approximately 1.6% of bone content. Nearly 80% of total body citrate is stored in bone [13]. Intracellular citrate is a key regulator of cellular energy status, and its concentration reflects the metabolic state of the cell. When ATP levels are high and energy demand is low, excess citrate is exported from mitochondria to the cytosol through the mitochondrial citrate carrier SLC25A1 [14]. In the cytosol, citrate supports lipid biosynthesis in highly proliferative cells [15] and contributes to specialized tissue functions, including extracellular matrix mineralization by osteoblasts in bone [16]. NaCT mediates citrate uptake from the extracellular space, including the bloodstream, into cells, thereby supporting cytoplasmic processes in tissues such as the liver and brain [17,18,19]. As NaCT likely functions exclusively as a citrate influx transporter, and NaCT is abundant in the secretory ameloblasts’ proximal, lateral, and Tomes’ process membranes. It has been proposed that the semipermeable proximal and distal tight junctions connecting adjacent ameloblasts allow citrate delivered by the blood supply to pass between ameloblasts to enter into the developing enamel space as NaCT imports some of it into the ameloblasts for energy metabolism and apparently reduces the extracellular citrate concentration to levels appropriate for successful enamel mineralization [20]. Normally, the citrate concentration in mature enamel is low, approximately 0.1% of enamel dry weight, whereas it is approximately 0.9% in dentin or cementum [20].

NaCT is required in soft tissues, including liver, brain, and muscle, and mineralized tissues, including bones and teeth. Notably, most of the body’s citrate is stored within the mineralized matrix of bone. Citrate is also an intermediate of the tricarboxylic acid (TCA) cycle, where it is generated from oxaloacetate, acetyl-coenzyme A (acetyl-CoA), and water by citrate synthase in the mitochondrial matrix. In developing teeth, citrate is transported into ameloblasts to support energy metabolism. Based on the established presence and function of citrate in bone, we speculate that citrate in developing enamel regulates enamel ribbon deposition and elongation. In bone, the mitochondrial citrate/malate antiporter SLC25A1/CIC transports citrate into the cytosol, whereas ANK, the progressive ankylosis protein, regulates the release of TCA cycle intermediates, including citrate, into the extracellular space [21,22]. ANK is highly expressed by osteoblasts and contributes to bone strength, in part by regulating citrate homeostasis [23]. In humans, *ANKH* variants cause craniometaphyseal dysplasia, MIM #123000; chondrocalcinosis 2, MIM #118600; and progressive arthritis [24]. In addition, cytosolic citrate accumulation resulting from ANK deficiency promotes aortic aneurysm [25]. *Ank* was not detected in secretory- or maturation-stage ameloblasts, suggesting that it is unlikely to mediate citrate export during normal enamel formation [26].

Previously, we characterized *Slc13a5*^Flag^ mice to precisely localize NaCT during tooth development and to evaluate enamel formed in the presence of SLC13A5-Flag [26]. NaCT was expressed by ameloblasts, odontoblasts, and osteoblasts. In ameloblasts, NaCT localized throughout the cell membrane in a stage-specific manner, with strong enrichment at the Tomes’ process. In the present study, we characterized the *Slc13a5*^R337*/R337*^ mouse model, which is homologous to the human *SLC13A5*^R333*/R333*^ mutation that causes DEE-25, to investigate the function of NaCT during enamel formation. We characterized developing enamel and ameloblasts, quantified changes in citrate content in developing tooth matrix, bone, and serum, and evaluated the expression of *Slc13a5*, *Ank*, and *Slc25a1* during amelogenesis. Based on evidence from the literature and preliminary data, we hypothesized that the absence of functional NaCT globally, and specifically in the odontoblast and ameloblast membranes, results in abnormally high extracellular citrate levels that impair dentin and enamel mineralization. This study was designed to advance understanding of citrate transport in ameloblasts and to provide mechanistic insight into DEE-25-associated tooth defects.

## 2. Results

### 2.1. Generation of the Slc13a5^R337*/R337*^ Mouse

**Figure 1:**
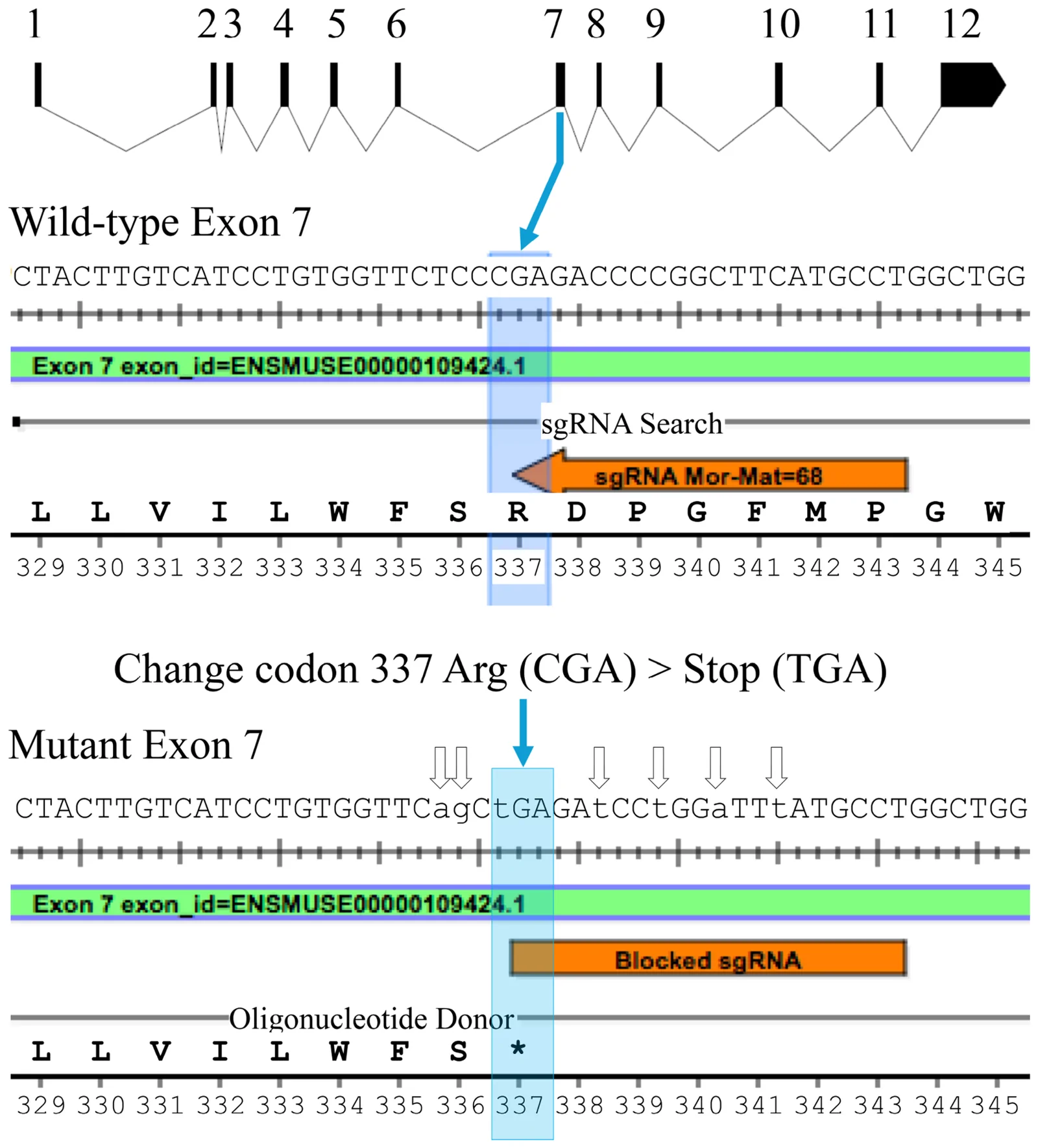
**CRISPR/Cas9 gene-editing strategy for *****Slc13a5***^R337*^ mouse model. Mouse *Slc13a5*^R337*^ is homologous to human *SLC13A5*^R333*^ and has 12 exons. All are coding. Green highlights a segment of mouse *Slc13a5* exon 7 that was modified to replace the wild-type Arg^337^ codon (CGA) with a premature stop (tGA) codon analogous to a mutation that causes autosomal recessive amelogenesis imperfecta in humans. The orange box indicates the location of the Cas9 target. Blue arrows indicate the positions (in blue boxes) of the wild-type CGA Arg^337^ codon, which is modified to a tGA opal translation termination codon. White arrows show six silent nucleotide changes (that do not alter the protein sequence) that were introduced to block sgRNA annealing and facilitate genotyping.

**Figure 2:**
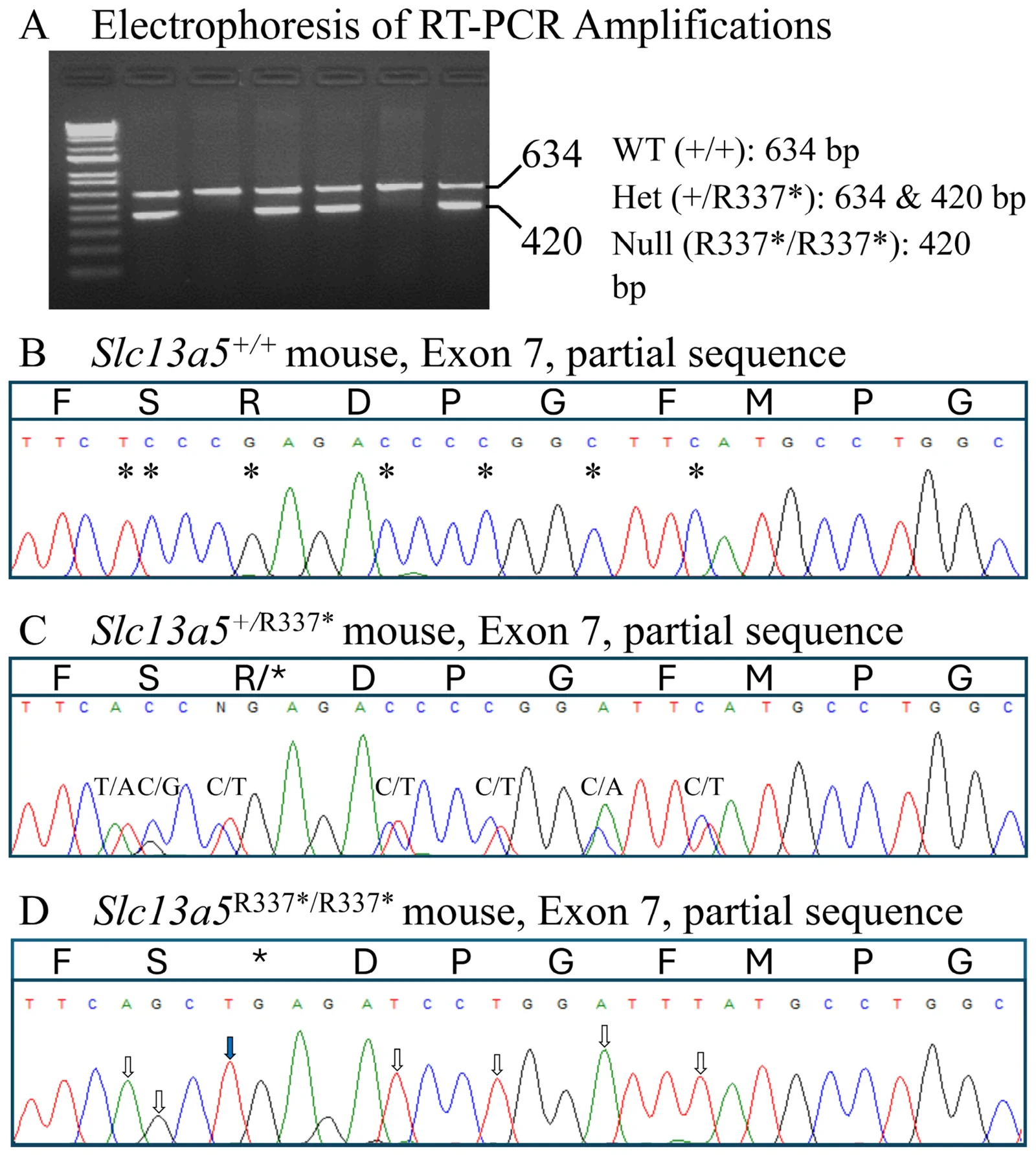
Sequencing confirmation of the CRISPR/Cas9 gene-edited *Slc13a5*^R337*^ mouse model. The sequence change in *Slc13a5* c.1009C>T; p.R337* produced a premature stop codon homologous to the human *SLC13A5*^333*^ mutation associated with severe hypoplastic amelogenesis imperfecta. (**A**) PCR genotyping strategy distinguishing *Slc13a5^+^*^/^*^+^* (*n* = 64), *Slc13a5^+^*^/R337*^ (*n* = 111), and *Slc13a*^R337*/R337*^ mice (*n* = 94). (**B**–**D**) Sanger sequencing of the F2 wild-type *Slc13a5^+^*^/^*^+^*, heterozygous *Slc13a5^+^*^/R337*^, and homozygous *Slc13a5*^R337*/R337*^ mice demonstrated accurate introduction of C>T nucleotide change (blue arrow). Additionally, 6 nucleotide changes (white arrows) without altering the amino acid codons were introduced accurately to prevent sgRNA annealing and to facilitate genotyping.

To generate *Slc13a5*^R337*/R337*^ mice, inbred C57BL/6N mice were used throughout the gene-editing process to minimize background sequence variations across the genome. The CRISPR/Cas9 construct was delivered by pronuclear microinjection into fertilized eggs collected from superovulated C57BL/6N female mice mated with C57BL/6N males. Thus, the only expected genomic differences between wild-type C57BL/6N mice and the resulting mutant mice were those introduced by CRISPR/Cas9 editing (Figure 1). Targeted *Slc13a5* mutants were identified by PCR genotyping of genomic DNA from tail biopsies. Positive mice were bred with wild-type C57BL/6N mice for two generations to dilute potential off-target effects that may have been caused by CRISPR/Cas9 editing and to confirm germline transmission. Tail biopsies from the offspring were characterized by PCR genotyping to verify the presence of mutated *Slc13a5*. All 12 exons and adjacent intron borders were characterized by DNA sequencing to identify mice carrying the intended *Slc13a5*^R337*^ allele (Figure 2).

The *Slc13a5*^R337*^ mouse model is available at MMRRC ID:67433, under the strain name C57BL/6N-*Slc13a5^em1Jcch^*/Mmucd. Growth and development of the homozygous mutant mice from this strain showed no observable difference from wild-type littermates. The average litter size was 6.3 pups based on 12 litters produced from 7 breeding cages over 6 months. By comparison, C57BL/6N breeding cages produced an average litter size of 7.8 pups, based on 140 pups from 18 litters generated by 12 breeding cages during the same period. No apparent growth or developmental delays, neurological abnormalities, or behavioral differences were observed among *Slc13a5*^R337*/R337*^ mice. In addition, body size did not differ between 7-week-old wild-type and *Slc13a5*^R337*/R337*^ mice (Figure S1).

### 2.2. Characterization of Slc13a5^R337*/R337*^ Mice

Transcript analyses demonstrated that *Slc13a5* was expressed in wild-type and *Slc13a5*^R337*/R337*^ D5 molars and 7-week-old mouse testes, but not in salivary glands or 7-week-old mouse seminal vesicles (Figure S2). *Ank* transcripts were detected by RT-PCR in D5, D8, and D12 whole molar samples from both wild-type and *Slc13a5*^R337*/R337*^ mice. *Ank* expression was further evaluated in D5 molars, incisors, and tail tissue from both genotypes (Figures S3 and S4). In these experiments, the molar and incisor samples included trace alveolar bone that could not be completely removed.

**Figure 3:**
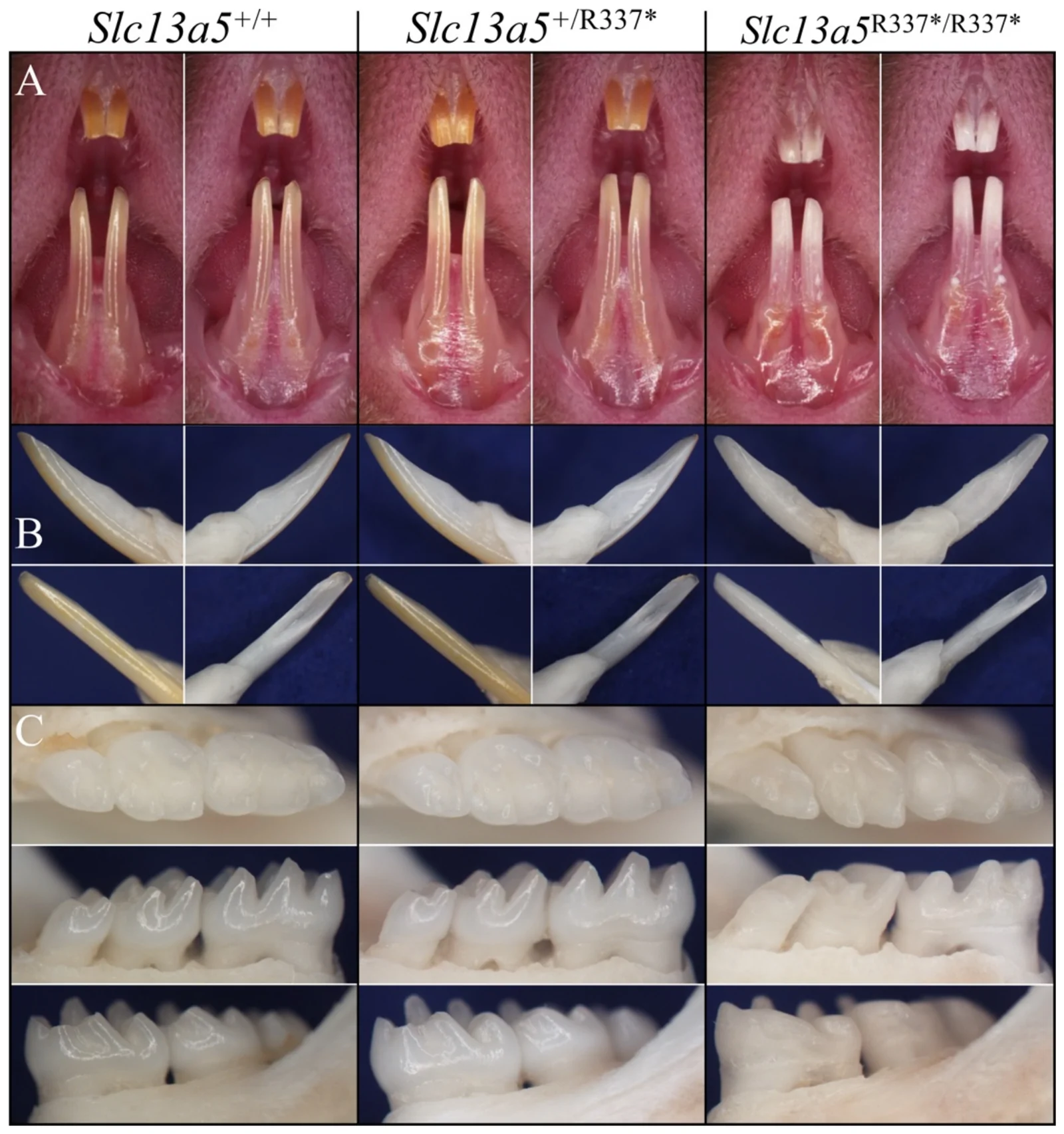
Dissection microscopy evaluation of *Slc13a5^+^*^/^*^+^*, *Slc13a5*^+/R337*^, and *Slc13a5*^R337*/R337*^ mouse teeth. Mouse incisors (**A**,**B**) and molars (**C**) were imaged at 7 weeks using a dissection microscope. No apparent differences between the WT (*Slc13a5*^+/+^ *n* = 6) and heterozygous (*Slc13a5*^+/R337*^ *n* = 8) incisors (**A**,**B**) or molars (**C**) were observed. In contrast, no enamel layer was observed on the homozygous (*Slc13a5*^R337*/R337*^ *n* = 7) incisors, which appear shorter and chalky-white (**A**,**B**), or the molars (**C**), which had short, rounded cusp tips flattened by occlusal attrition. Key: (**A**) Incisor facial surfaces, in situ. (**B**) Top: incisor mesial (left) and distal (right) surfaces; bottom: incisor labial (left) and lingual (right) surfaces. (**C**) Mandibular molars. Top: occlusal; middle: lingual; and bottom: buccal surfaces.

*Slc13a5*^R337*/R337*^ mice typically exhibited chalky white incisors and occlusally flattened molars by 7 weeks of age (Figure 3). No detectable enamel was present on the teeth of *Slc13a5*^R337*/R337*^ mice. In contrast, no differences in molar size, shape, or color were observed between the wild-type and *Slc13a5*^+/R337*^ mice.

**Figure 4:**
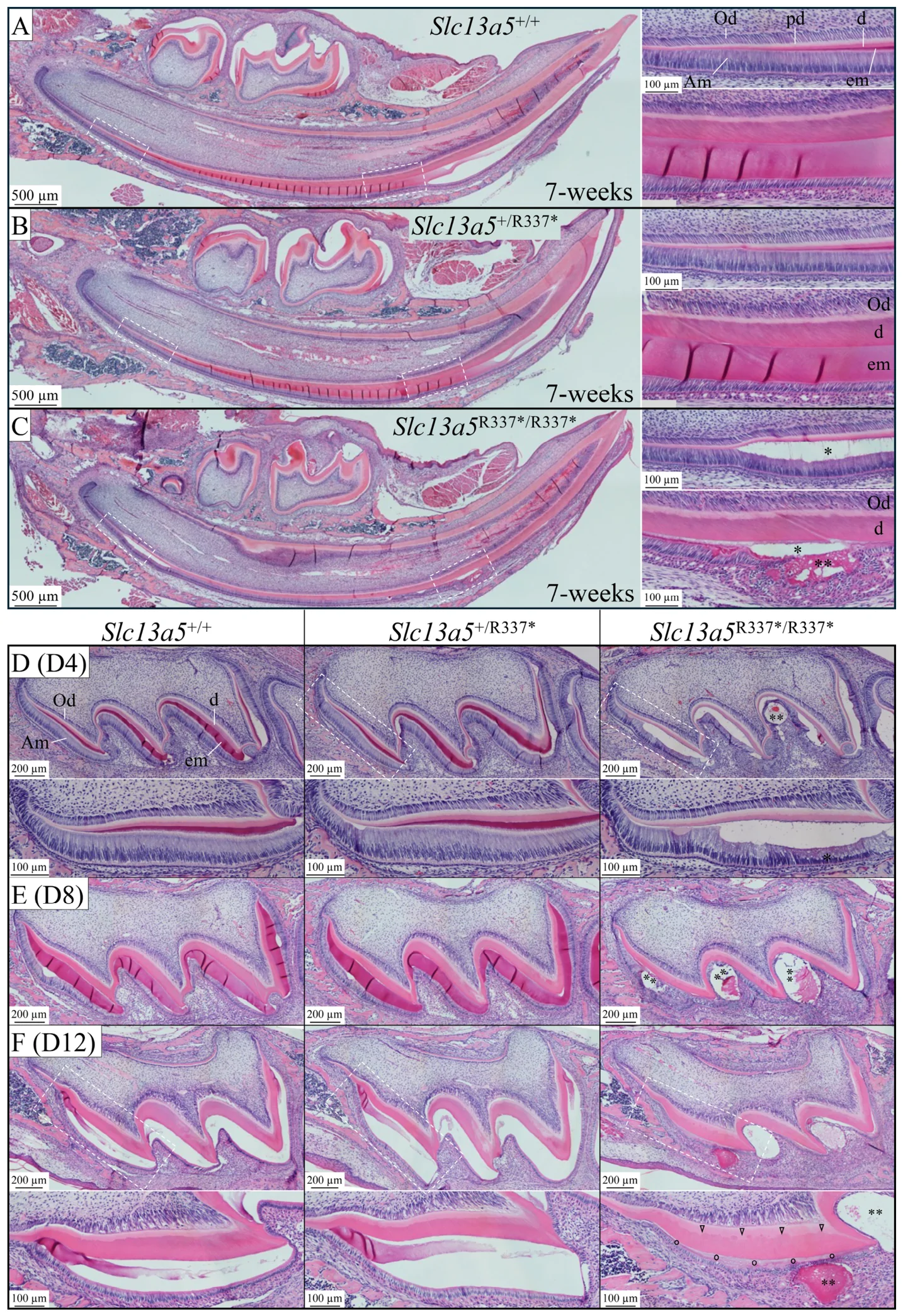
Light microscopy of *Slc13a5*^+/+^, *Slc13a5*^+/R337*^, and *Slc13a5*^R337*/R337*^ mouse mandibular incisors at 7 weeks (**A**–**C**) and maxillary molars at Day 4 (**D**), 8 (**E**), and 12 (**F**). No apparent differences between WT (*Slc13a5*^+/+^ *n* = 7 from 3 time points) and heterozygous (*Slc13a5*^+/R337*^ *n* = 9 from 3 time points) incisors or molars were observed at any stage of development, which is consistent with the human autosomal recessive pattern of inheritance. Due to the importance of the SLC13a5 protein to successful early enamel mineral formation, no true enamel forms in the null incisors or molars (*Slc13a5*^R337*/R337*^ *n* = 8 from 3 time points) at any stage of development. The “enamel” forming on the dentin surface is thin and irregular and continues to increase in volume during the maturation stage (◯), often ectopically in the soft tissue of the enamel organ, rather than simply hardening after reaching its final dimensions during the secretory stage. Dentin in the null mice appears to be normal thickness, but stains a lighter pink and exhibits an irregular predentin/dentin border (▽). Secretory and maturation ameloblasts in the incisor are more susceptible to artifactual separation from the underlying mineral during histological preparation (*) and cyst-like structures (**) form in the maturation stage, and mineral nodules form within the enamel organ epithelia. Key: Am, ameloblast; d, dentin; em, enamel matrix; Od, odontoblast; pd, pre-dentin. Dashed boxes outline the higher magnification images on the right.

**Figure 5:**
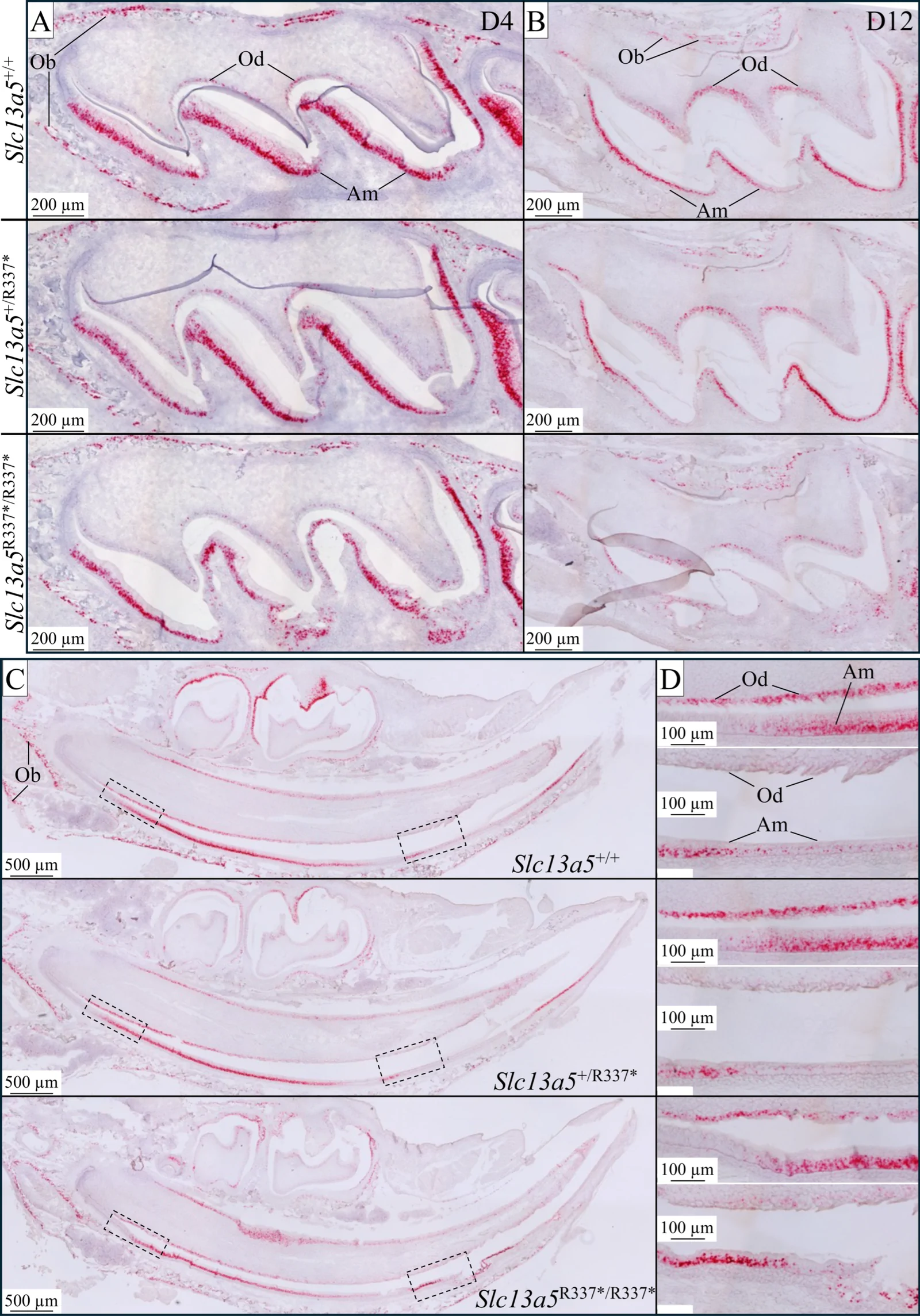
Evaluation of *Slc13a5* expression in *Slc13a5*^+/+^, *Slc13a5*^+/R337*^, and *Slc13a5*^R337*/R337*^ mouse teeth. *Slc13a5* expression demonstrated by in situ hybridization in D4 (**A**) and D12 (**B**) maxillary molars revealed a comparable expression pattern and intensity among samples of all three genotypes. A similar expression pattern can be observed in D12 (**C**,**D**) mandibular incisors. Positive signals concentrated in the osteoblast, odontoblast, and ameloblast. *Slc13a5* expression is stronger in secretory stage ameloblasts compared to maturation stage ameloblasts. Key: Ob, osteoblast; Od, odontoblast; Am, ameloblast. Riboprobes were tested with a negative control in a pilot experiment. At each time point, sample size was two from each genotype.

Histologically, the wild-type and *Slc13a5*^+/R337*^ hemimandibles were comparable in development (Figure 4). In *Slc13a5*^R337*/R337*^ mice, however, enamel matrix deposition was absent at D4. By day 8, ectopic matrix accumulation was evident along the cusp slopes, and by D12 nodules were observed on the cusps and slopes. Similar abnormalities were observed on the labial surface of D12 mandibular incisors. Dysplastic ameloblasts also detached from the dentin surface, and no appreciable enamel layer was present (Figure 4E). Although *Slc13a5*^R337*/R337*^ ameloblasts exhibited significant morphological changes in polarization and matrix deposition, they maintained *Slc13a5* transcript expression (Figures S2, S3 and Figure 5) with intensity and distribution pattern similar to those observed in wild-type and *Slc13a5*^+/R337*^ D4 mouse maxillary molars and D12 incisors (Figure 5 and Figure S5).

**Figure 6:**
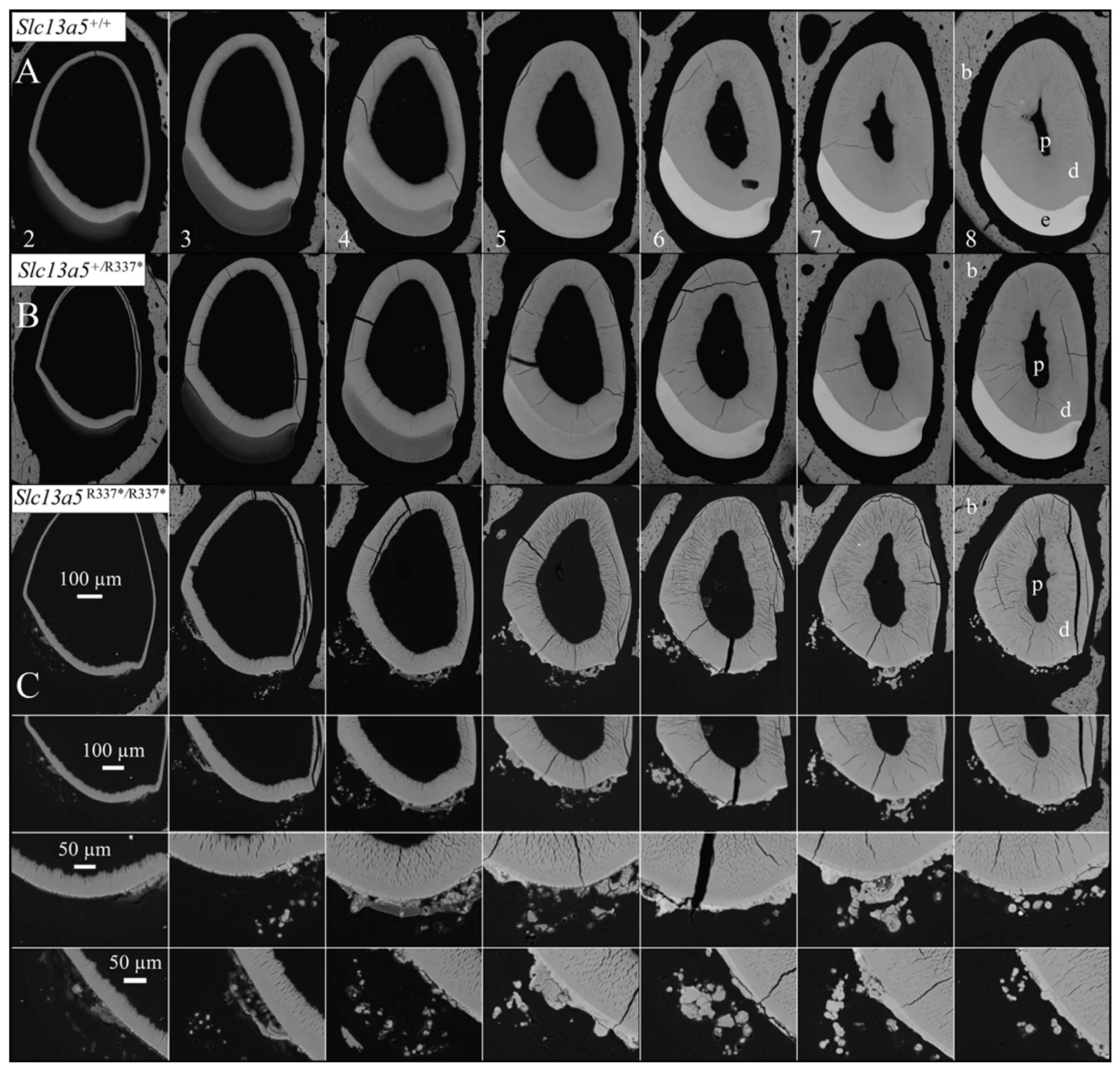
Characterization of *Slc13a5*^+/+^, *Slc13a5*^+/R337*^, and *Slc13a5*^R337*/R337*^ mouse mandibular incisors. Backscattered scanning electron (bSEM) micrographs of 7-week mandibular incisors cross-sectioned at 1 mm increments (sections are progressively later in development moving to the right). *Slc13a5*^+/+^ (**A**), *Slc13a5*^+/R337*^ (**B**), and *Slc13a5*^R337*/R337*^ (**C**). Panel (**C**) has 4 rows, each at higher magnification, to detail the enamel pathology in the *Slc13a5*^R337*/R337*^ mouse. Column 2 (on the left) is at mid-secretory stage. Column 3 is at the end of the secretory stage when the enamel has reached its final thickness. Columns 3–8 are in successively later stages of enamel maturation (hardening). Notice the unchanging thickness of enamel (e) but increasing density (whiteness) moving to the right. The dark centers of the cross-sections were occupied by dental pulp (p). Dentin (d) surrounds the pulp. Enamel (e) covers the labial surface of dentin. In panels (**A**, **B**), the enamel and dentin layers in *Slc13a5*^+/+^ and *Slc13a5*^+/R337*^ are comparable, but in (**C**), the *Slc13a5*^R337*/R337*^ incisors form only a thin crust of poorly mineralized material covering dentin, and numerous mineral nodules of various sizes in the overlying soft tissue. In addition, dentinal tubules (dark lines radiating from the pulp toward the dentin surface) are more prominent in the *Slc13a5*^R337*/R337*^ samples. Key: b, alveolar bone; p, pulp; d, dentin; e, enamel. Six samples from each genotype were analyzed in this experiment.

**Figure 7:**
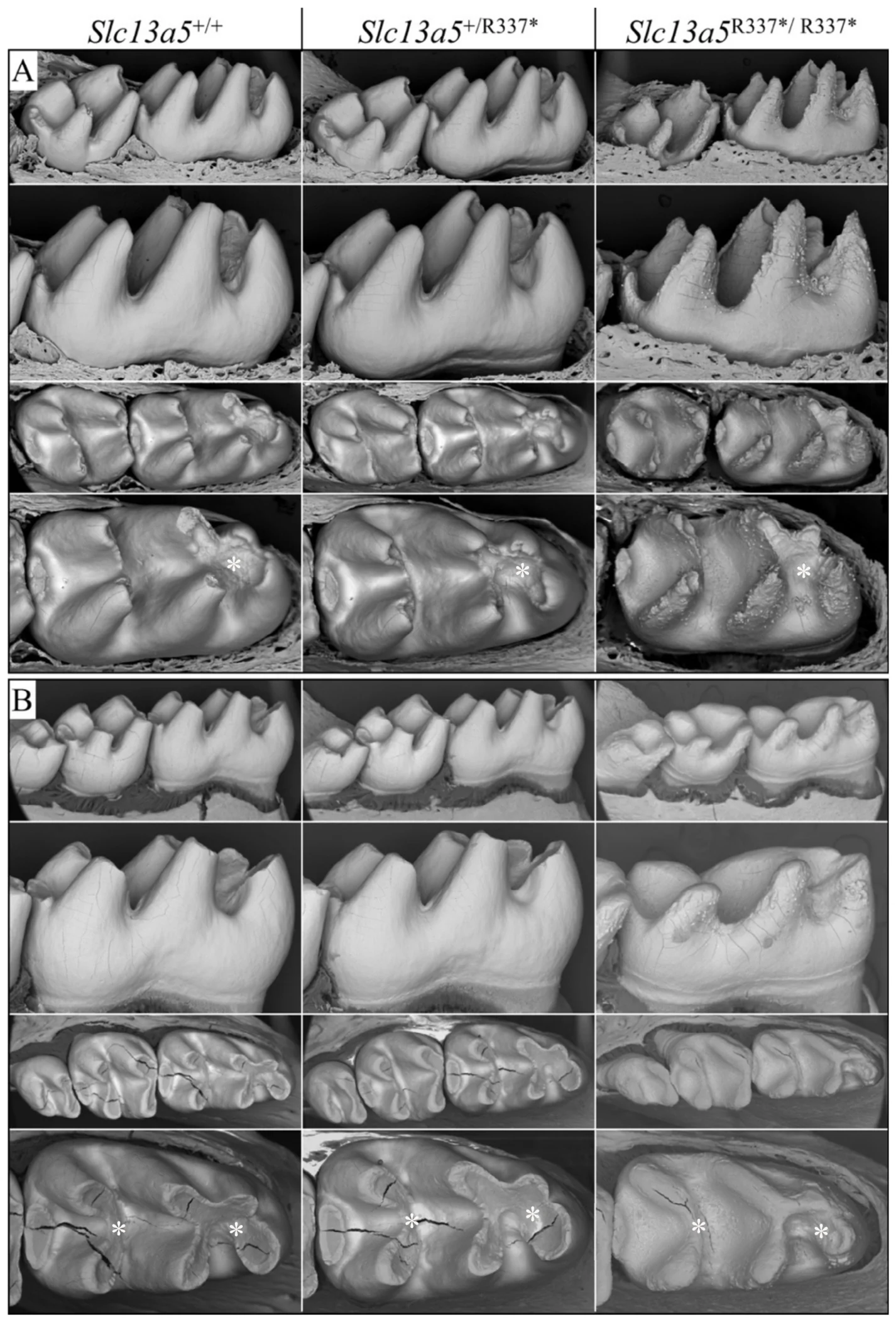
bSEM images of *Slc13a5*^+/+^, *Slc13a5*^+/R337*^, and *Slc13a5*^R337*/R337*^ mandibular molars. (**A**) Day 14 (just before eruption into function) lingual views (top 2 rows) of mandibular 1st and 2nd molars and 1st molar, respectively, and occlusal views (next 2 rows) of the same teeth. The *Slc13a5*^+/+^ and *Slc13a5*^+/R337*^ molar crowns show normal shape and contour. *Slc13a5*^R337*/R337*^ crowns have thin cusps lacking enamel and show mineral nodules instead of enamel. (**B**) Lingual views (top 2 rows) of 7-week-old (after 5 weeks in function) mandibular 1st, 2nd, and 3rd molars and 1st molar, respectively, and occlusal views (next 2 rows) of the same. The *Slc13a5*^+/+^ and *Slc13a5*^+/R337*^ molar crowns show normal shape and contour with similar patterns of wear. *Slc13a5*^R337*/R337*^ crowns have thin cusps lacking enamel and show significant attrition on their occlusal surface. Enamel-free areas (*) on the cusp tips can be observed in the occlusal view of day 14 and 7-week-old mandibular first molars from *Slc13a5*^+/+^, *Slc13a5*^+/R337*^, and *Slc13a5*^R337*/R337*^ mice [27]. Three samples from each genotype were analyzed.

Both 7-week-old wild-type and *Slc13a5*^+/R337*^ mice showed normal development and mineralization of dentin and enamel in mandibular incisors. In contrast, *Slc13a5*^R337*/R337*^ mouse incisors exhibited widened dentinal tubules and a complete absence of normal enamel formation (Figure 6A). The hypoplastic enamel phenotype observed grossly (Figure 3B,C) was consistent with the histological findings shown in Figure 4D,E and Figure 6A–C. Surface scanning of D14 and 7-week-old molars from *Slc13a5*^R337*/R337*^ mice revealed ectopic nodules of variable sizes on the surface of the first molars (Figure 7). These molars were smaller and showed severe occlusal attrition compared with wild-type and *Slc13a5*^+/R337*^ molars.

**Figure 8:**
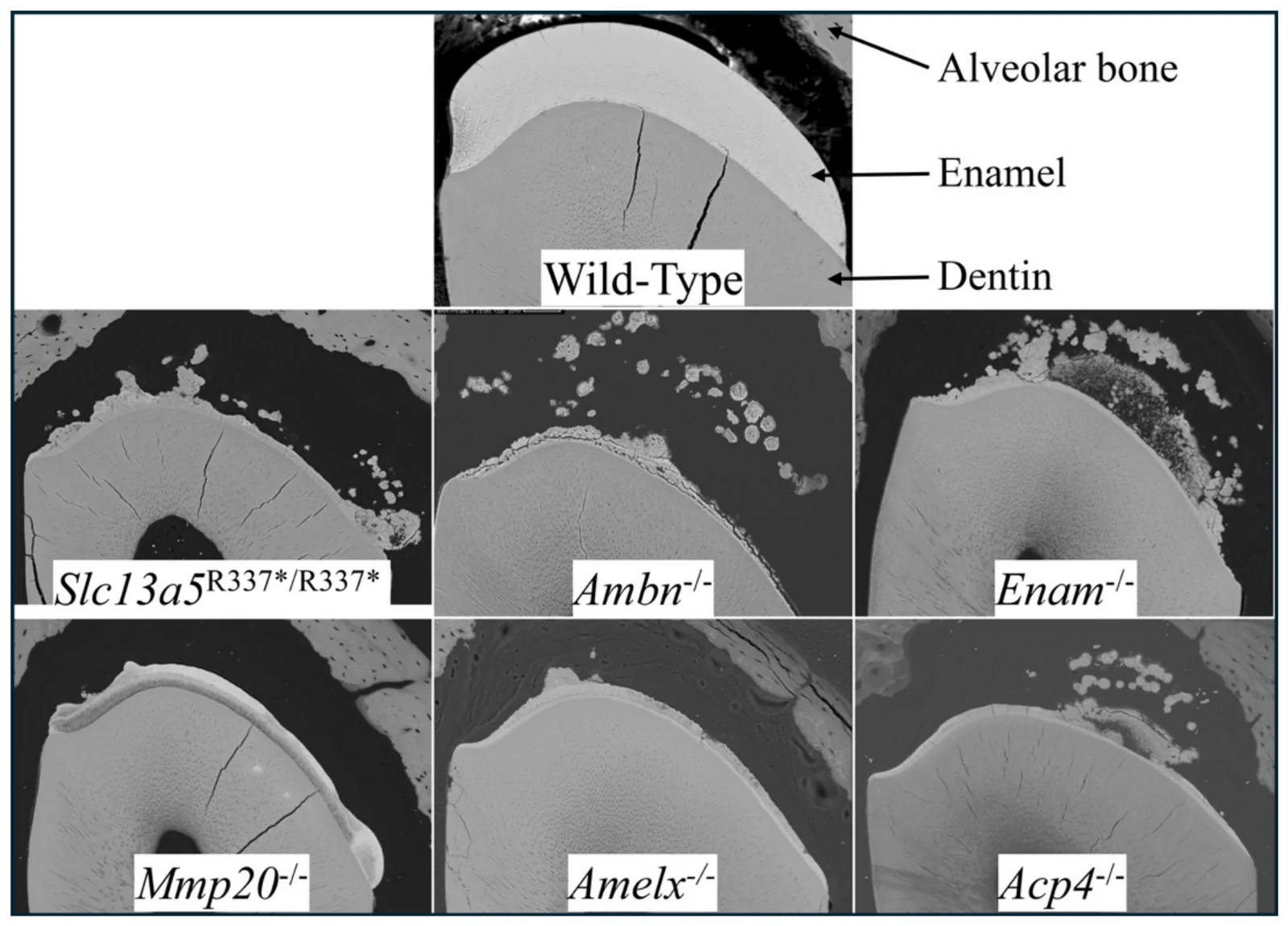
Backscattered SEM images of 7-week-old mouse mandibular incisors from WT, *Slc13a5*, *Ambn*, *Enam*, *Mmp20*, *Amelx*, and *Acp4* knockout mice at level 8 (near eruption, maturation stage complete). Images compare wild-type and knockout mice of genes critical for secretory stage function. As is generally true of genes critical for enamel ribbon deposition and elongation in the secretory stage, *Slc13a5*^R337*/R337*^ mice produced no true enamel. The ectopic calcification nodules are rapidly lost following tooth eruption. Images were adapted, with permission, from the top panel of Figure 3, Simmer et al. (2021) [11]. This experiment was conducted multiple times. Images from one sample of each genotype were presented here.

**Figure 9:**
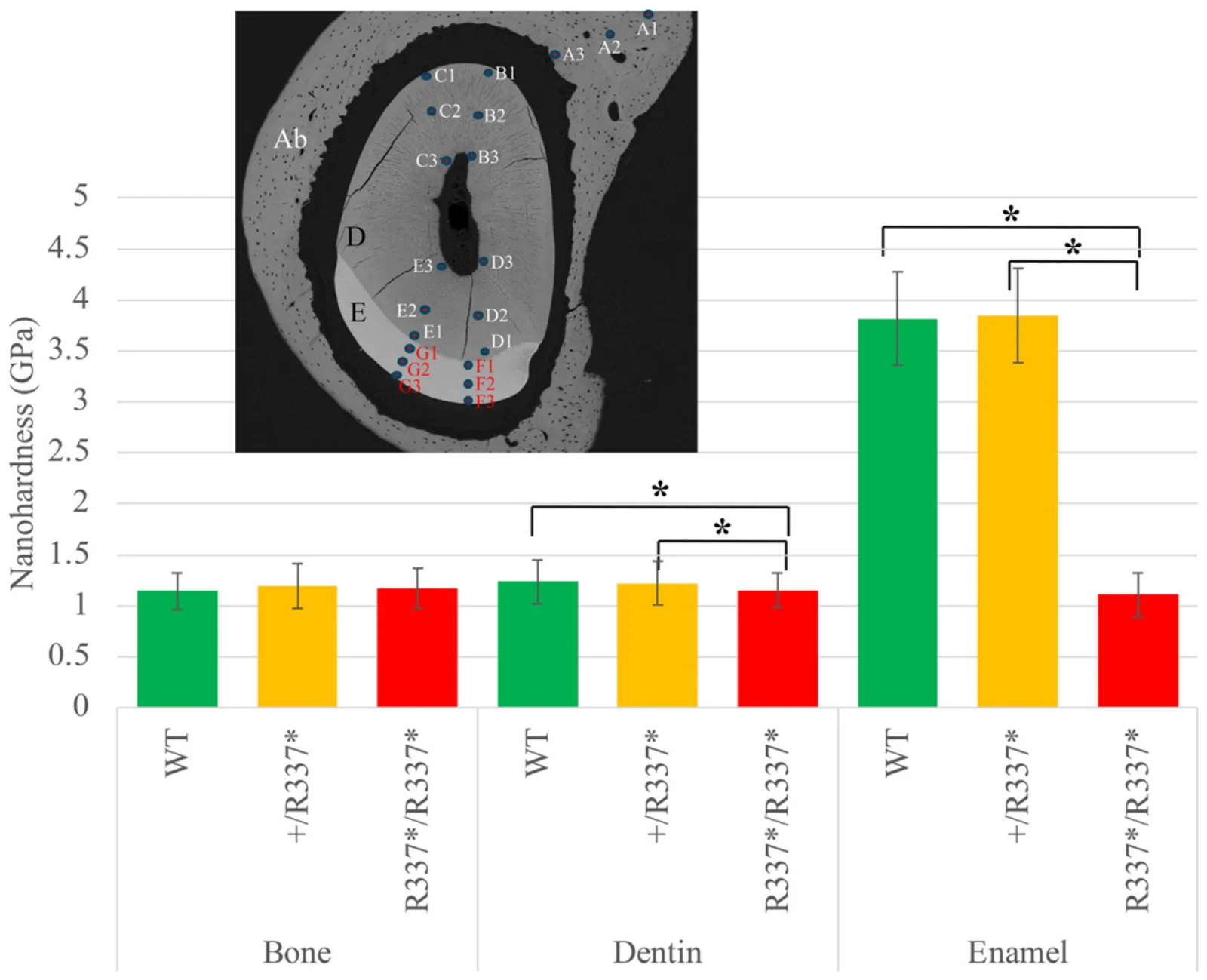
Knoop hardness testing of 7-week *Slc13a5*^+/+^, *Slc13a5*^+/R337*^, and *Slc13a5*^R337*/R337*^ mandibular incisors following completion of enamel maturation. The inserted panel shows positions of indentations made by a 10 gm force application in incisor cross-sections at the level of the alveolar crest (level 8) for the three genotypes. The hardness of the alveolar bone (A), dentin (B–E), and the inner (F1, G1), middle (F2, G2), and outer (F3, G3) enamel were evaluated. Three indentations were generated at each sample level and measured to calculate Knoop hardness values (HKs). The bar graphs show mean HKs over different areas of different genotypes. There were no statistically significant differences in bone HKs among the three genotypes. The HKs of dentin and enamel from *Slc13a5*^R337*/R337*^ were statistically lower than those of the other two genotypes. The *Slc13a5*^R337*/R337*^ abnormal enamel had significantly reduced HK values, which averaged only 35% of those for the wild-type and *Slc13a5*^+/R337*^ enamel. * Denotes *p* < 0.05. Key: Ab: alveolar bone; D: dentin; E: enamel. Sample sizes were *Slc13a5*^+/+^ *n* = 6, *Slc13a5*^+/R337*^ *n* = 6, and *Slc13a5*^R337*/R337*^ *n* = 5.

Analysis of the enamel layer formed on mandibular incisors at comparable maturation-stage locations across different genotypes (Figure 8 and Figures S6–S8) showed that *Amelx*^−/−^ and *Mmp*20^−/−^ incisors contained a thin layer of poorly organized aprismatic enamel. In contrast, *Enam*^−/−^, *Ambn*^−/−^, *Acp4*^−/−^, and *Slc13a5*^−/−^ incisors had only a thin layer of poorly mineralized material covering the dentin, with ectopic mineralized nodules of varying sizes deposited more peripherally. The hardness of these nodules was comparable to that of bone and dentin but was significantly lower than that of enamel in wild-type and *Slc13a5*^+/R337*^ mice (Figure 9). In addition, dentin hardness, but not bone hardness, was decreased in *Slc13a5*^R337*/R337*^ mice.

One of the most unexpected findings from the histological analyses was the absence of normal enamel matrix production and its replacement by ectopic mineral nodule formation on the incisors and molars of *Slc13a5*^R337*/R337*^ mice (Figure 4C,F). To investigate this defect further, we assessed the distribution of the enamel matrix proteins amelogenin and enamelin, as well as the lysosomal marker LAMP1, by immunohistochemistry in D12 mandibular incisors from wild-type and *Slc13a5*^R337*/R337*^ mice.

**Figure 10:**
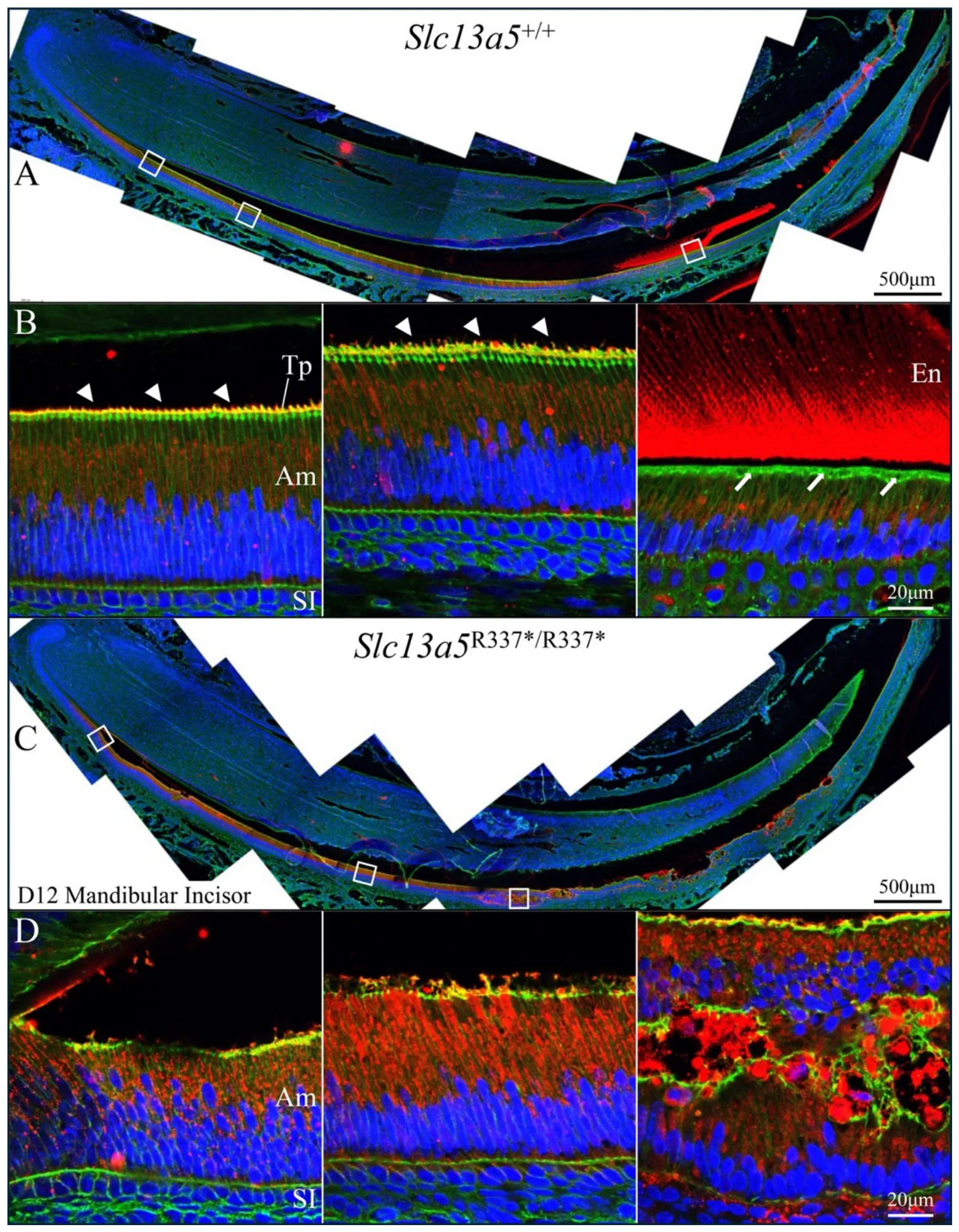
Amelogenin expression in *Slc13a5*^+/+^ and *Slc13a5*^R337*/R337*^ D12 mandibular mouse incisors. (**A**) *Slc13a5*^+/+^ mandibular incisor longitudinal section demonstrating positive expression of amelogenin in the early secretory stage, which persists into the maturation stage. White boxes mark positions of higher magnification images below. (**B**) Amelogenin localized to Tomes’ processes (arrowheads), inside secretory and maturation stage ameloblasts, and in the maturation stage enamel matrix (arrows). (**C**) *Slc13a5*^R337*/R337*^ mandibular incisor longitudinal section demonstrating irregularities in the ameloblast layer that increased in severity over time (toward the right); however, strong positive expression of amelogenin was still observed. (**D**) Tomes’ processes were disrupted in secretory stage ameloblasts. Amelogenin appeared to accumulate in the ameloblast cytoplasm and intercellular spaces. Key: Tp, Tomes’ processes; Am, ameloblasts; En, enamel; red fluorescence, amelogenin; green, β-actin; blue, DAPI-stained nuclei. Pilot experiments were conducted to optimize conditions; the final experiment was conducted on sections from one sample.

**Figure 11:**
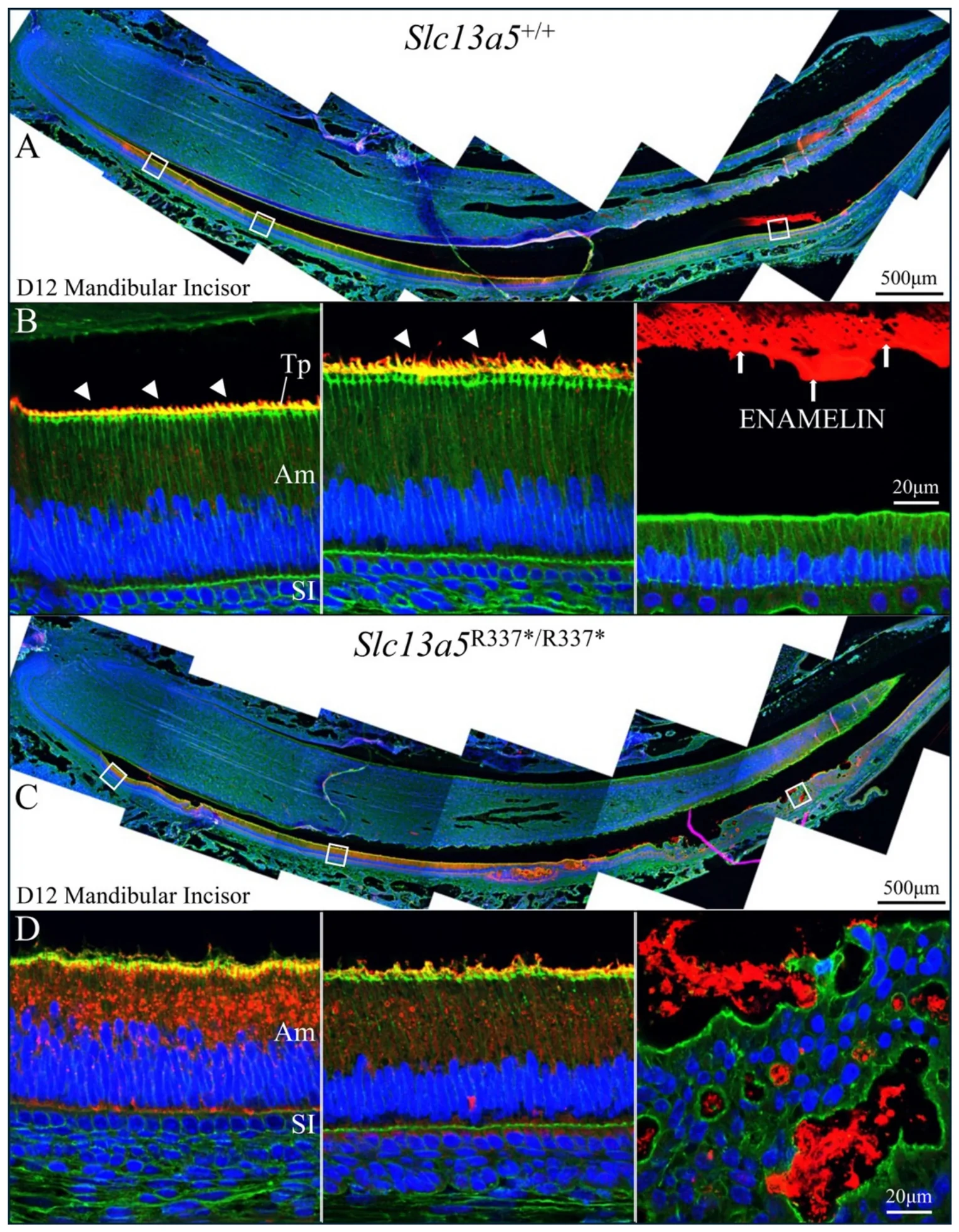
Enamelin expression in *Slc13a5*^+/+^ and *Slc13a5*^R337*/R337*^ D12 mandibular mouse incisors. (**A**) *Slc13a5*^+/+^ mandibular incisor demonstrating positive expression of enamelin in secretory stage ameloblasts and then terminating as the ameloblasts enter postsecretory transition into the maturation stage. (**B**) Enamelin localized to the Tome’s process (yellow, arrowheads), the cytoplasm of the ameloblasts, and enamel matrix (arrows) in the maturation stage. (**C**) *Slc13a5*^R337*/R337*^ mandibular incisor demonstrating positive expression of enamelin similar to that of the wild-type, but with ectopic accumulations extracellularly. (**D**) There were no Tomes’ processes evident in the polarized ameloblasts. Unlike wild-type mice, large amounts of enamelin accumulated within the cytoplasm and extracellular spaces as ameloblasts became dysplastic. Key: Tp, Tomes’ processes; Am, ameloblasts; En, enamel; red fluorescence, enamelin; green, β-actin; blue, DAPI-stained nuclei. Pilot experiments were conducted to optimize conditions; the final experiment was conducted on sections from one sample.

**Figure 12:**
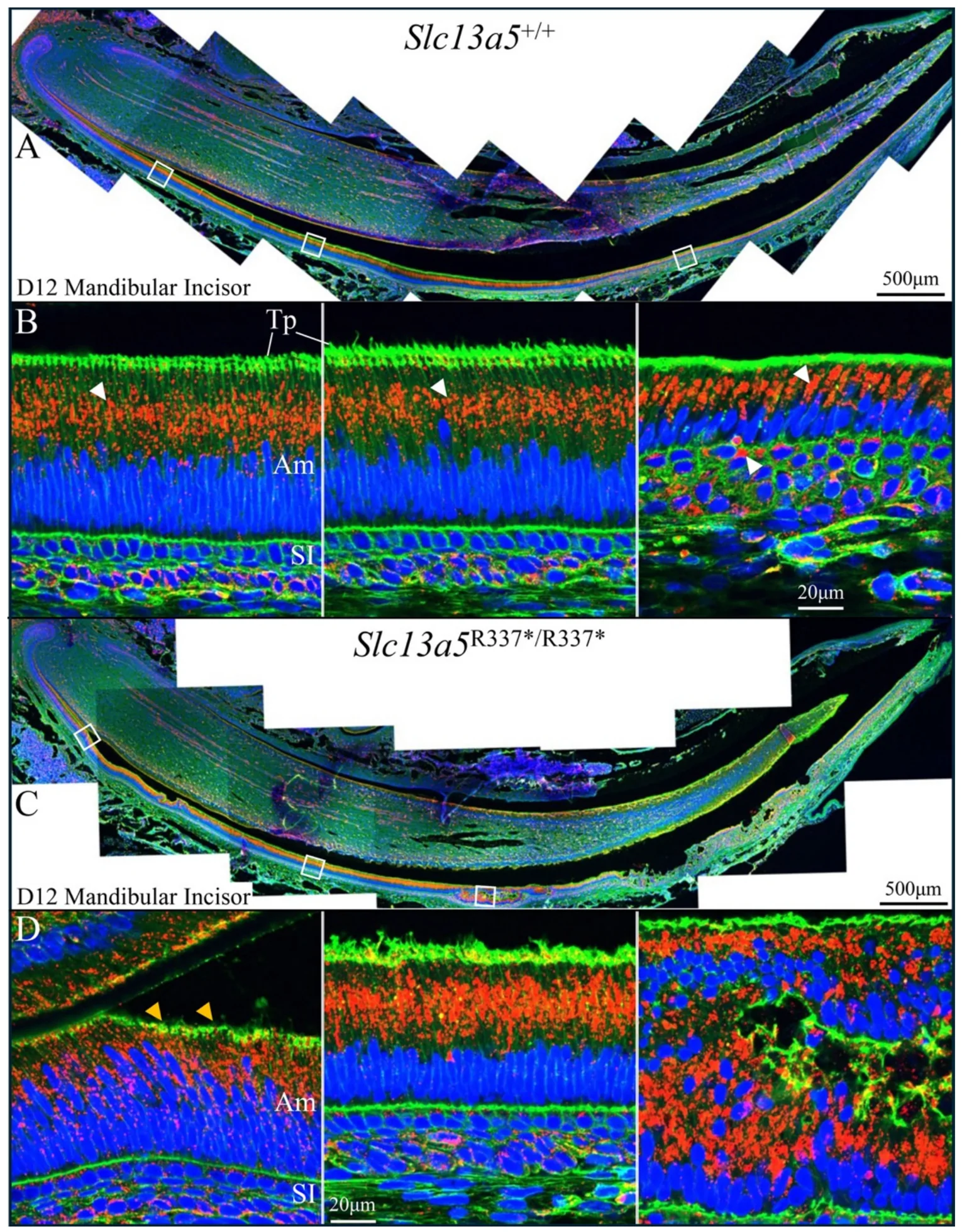
LAMP1 expression in D12 mandibular incisors from *Slc13a5*^+/+^ and *Slc13a5*^R337*/R337*^ mice. (**A**) *Slc13a5*^+/+^ mandibular incisor demonstrating positive expression of LAMP1 in differentiating presecretory stage ameloblasts and extending across the secretory stage and most of the maturation stage ameloblasts. (**B**) LAMP1 localized to the lysosomes (globular red stain) in the supranuclear cytoplasm of secretory and maturation ameloblasts (arrowheads) and many papillary layer cells during the maturation stage (upward arrowhead). (**C**) *Slc13a5*^R337*/R337*^ expression of LAMP1 was like that of the wild-type during the secretory stage. (**D**) Tomes’ processes (yellow arrowheads) were not evident in polarizing ameloblasts. LAMP1 signal was stronger than that of the WT in secretory ameloblasts and especially so in the dysplastic maturation stage ameloblasts. White arrowheads, LAMP1; yellow arrowheads, lack of Tomes’ processes. Key: Tp, Tomes’ processes; Am, ameloblasts; red fluorescence, LAMP1; green, β-actin; blue, DAPI-stained nuclei. Pilot experiments were conducted to optimize conditions; the final experiment was conducted on sections from one sample.

In wild-type incisors, amelogenin expression began during the early secretory stage and continued into the maturation stage (Figure 10A). During maturation, amelogenin was detectable within the enamel layer, presumably in association with enamel ribbons (Figure 10B). In contrast, in the absence of functional NaCT, secretory ameloblasts failed to form Tomes’ processes, detached from the matrix surface, and retained large amounts of amelogenin intracellularly. At the late secretory stage, as ameloblasts became dysplastic, amelogenin was aberrantly released intercellularly as well as both proximally and distally (Figure 10C,D). Similar abnormalities were observed for enamelin, including failure of Tomes’ process formation, intracellular retention of enamelin, and abnormal enamelin release by late secretory ameloblasts (Figure 11). Secretory ameloblasts normally exhibit high lysosomal activity; however, in the absence of functional NaCT, the lysosomal compartment was expanded, and this increase persisted into the maturation stage (Figure 12).

**Figure 13:**
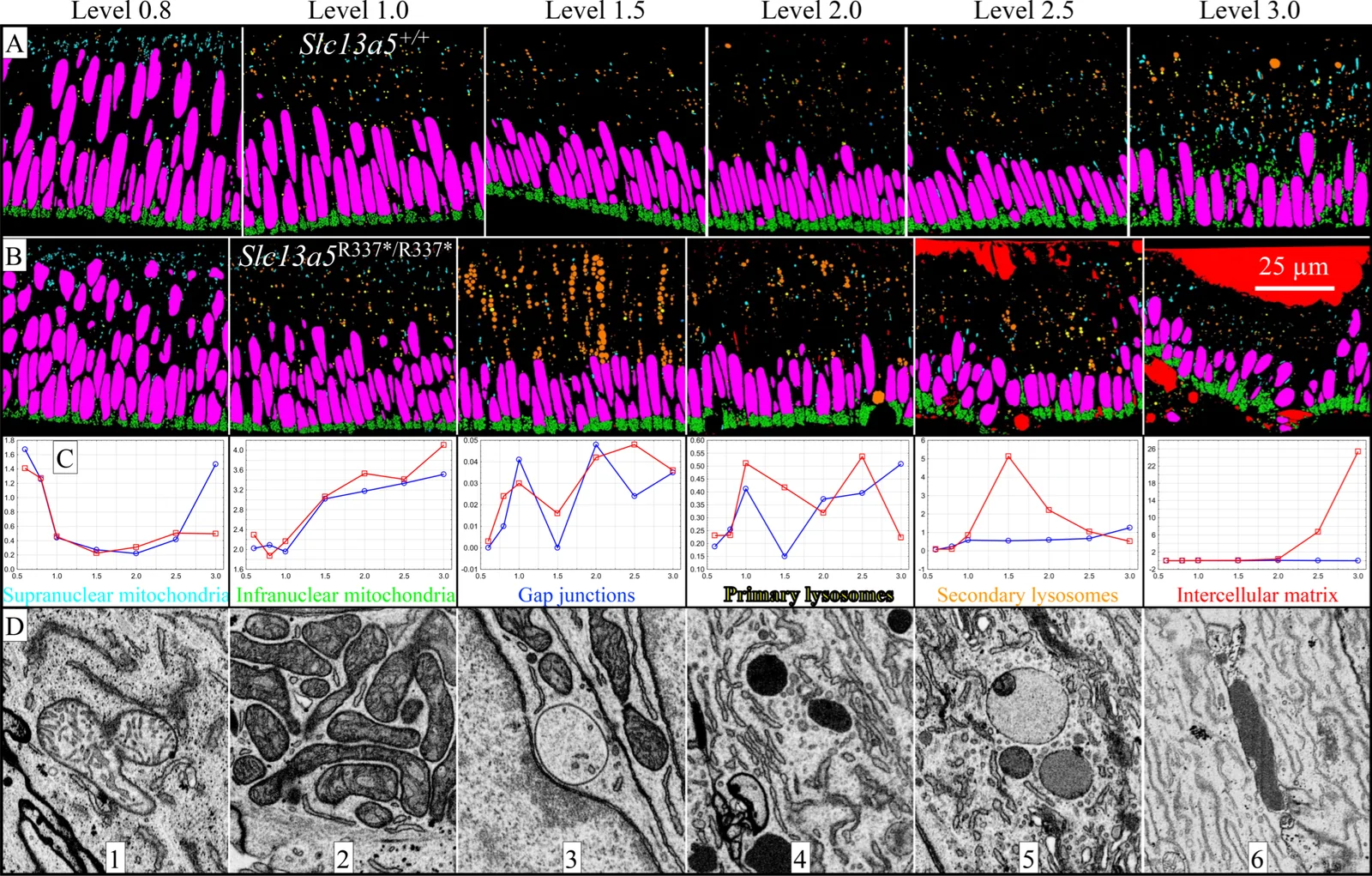
Organelle analyses of FIB-SEM images of a longitudinally sectioned mandibular incisor showing ameloblasts at successive intervals from polarizing ameloblasts (Level 0.8), the onset of enamel ribbon formation (Level 1.0), and through the end of appositional growth (Level 3.0). (**A**,**B**) SEM images of 6 organelles were colored [supranuclear mitochondria (cyan), infranuclear mitochondria (green), gap junctions (blue), primary lysosomes (yellow), secondary lysosomes (orange), and intercellular matrix (red)] to show 6 successive segments of secretory ameloblasts from *Slc13a5*^+/+^ (**A**) and *Slc13a5*^R337*/R337*^ (**B**) mouse incisors. (**C**) Plots of the volumes of each color at each interval (*Slc13a5*^+/+^, blue; *Slc13a5*^R337*/R337*^, red) as measured using ImageJ v1.53 (NIH). The numbers on the X-axes correspond to the “Level”; the Y-axes are % volume of the colored structures. (**D**) High-magnification SEMs of the organelles that were colored in each micrograph. **1:** supranuclear mitochondria (cyan), **2:** infranuclear mitochondria (green), **3:** gap junctions (blue), **4:** primary lysosomes (yellow), **5:** secondary lysosomes (orange), and **6:** intercellular matrix (red). Three samples of each genotype were processed and imaged. One set of images from each genotype was subjected to pseudo-coloring and organelle analyses.

FIB-SEM panels spanning Levels 0.8 to 3.0 mm along the mandibular incisors of wild-type and *Slc13a5*^R337*/R337*^ mice are shown in Figure 13A,B. The level numbers indicate approximate distances, in millimeters, from the tip of the apical loop. These levels also correspond to specific physical or cellular landmarks during enamel development: Level 0.8 marks the site where the basement membrane begins to disintegrate; Level 1.0 corresponds to the onset of appositional growth of the enamel layer; Level 1.5 represents the stage shortly after initiation of the inner enamel layer, when approximately the first quarter of the secretory stage has been completed; Level 2.0 corresponds to the midpoint of the secretory stage; Level 2.5 marks the transition from inner to outer enamel layer formation; and Level 3.0 represents the end of the secretory stage, when ameloblasts have retracted their Tomes’ processes while completing rod elongation.

Quantification of the percentage area occupied by selected organelles and cellular structures in FIB-SEM images showed comparable values for supranuclear and infranuclear mitochondria in wild-type and *Slc13a5*^R337*/R337*^ mice. The only exception was an increase in supranuclear mitochondria in wild-type ameloblasts at Level 3, which was not observed in *Slc13a5*^R337*/R337*^ mice. Instead, a large accumulation of intercellular protein was evident at this stage in *Slc13a5*^R337*/R337*^ mice. No notable differences were observed in nuclei, gap junctions, or primary lysosomes. However, secondary lysosomes were significantly increased at Level 1.5 in *Slc13a5*^R337*/R337*^ mice, *p* < 0.05, as indicated by the yellow boxes. Remarkably, no differences in the percentage area occupied by any analyzed subcellular organelles were detected at Levels 0.8 or 1.0, despite the complete failure of enamel mineral ribbon formation at these stages (Figure 13).

### 2.3. Dysregulation of Biological Activities in Slc13a5^R337*/R337*^ Mice

A comparative in situ hybridization approach was used to assess transcript expression of *Slc13a5* and four other potential citrate transporters in D4 maxillary molars and D12 mandibular incisors. SLC13A2, a dicarboxylate transporter, is capable of exchanging citrate for succinate; SLC13A3, a sulfate transporter, can also transport tricarboxylates; ANK is known to mediate citrate export in osteoblasts; and SLC25A1 exports citrate from mitochondria into the cytosol.

*Slc13a5* expression was consistently detected in secretory ameloblasts, odontoblasts, and osteoblasts of the alveolar bone. Its expression decreased during the transition stage but increased again during the maturation stage. In contrast, *Ank* expression was only detected in preameloblasts, preodontoblasts, and osteoblasts, and only overlapped with *Slc13a5* expression in osteoblasts. The strong *Ank* expression in osteoblasts likely explains the positive RT-PCR amplification observed in D5, D8, and D12 tooth organs, as these samples consistently included alveolar bone in the cervical and furcation regions. No *Ank* expression was detected in secretory- or maturation-stage ameloblasts or odontoblasts.

**Figure 14:**
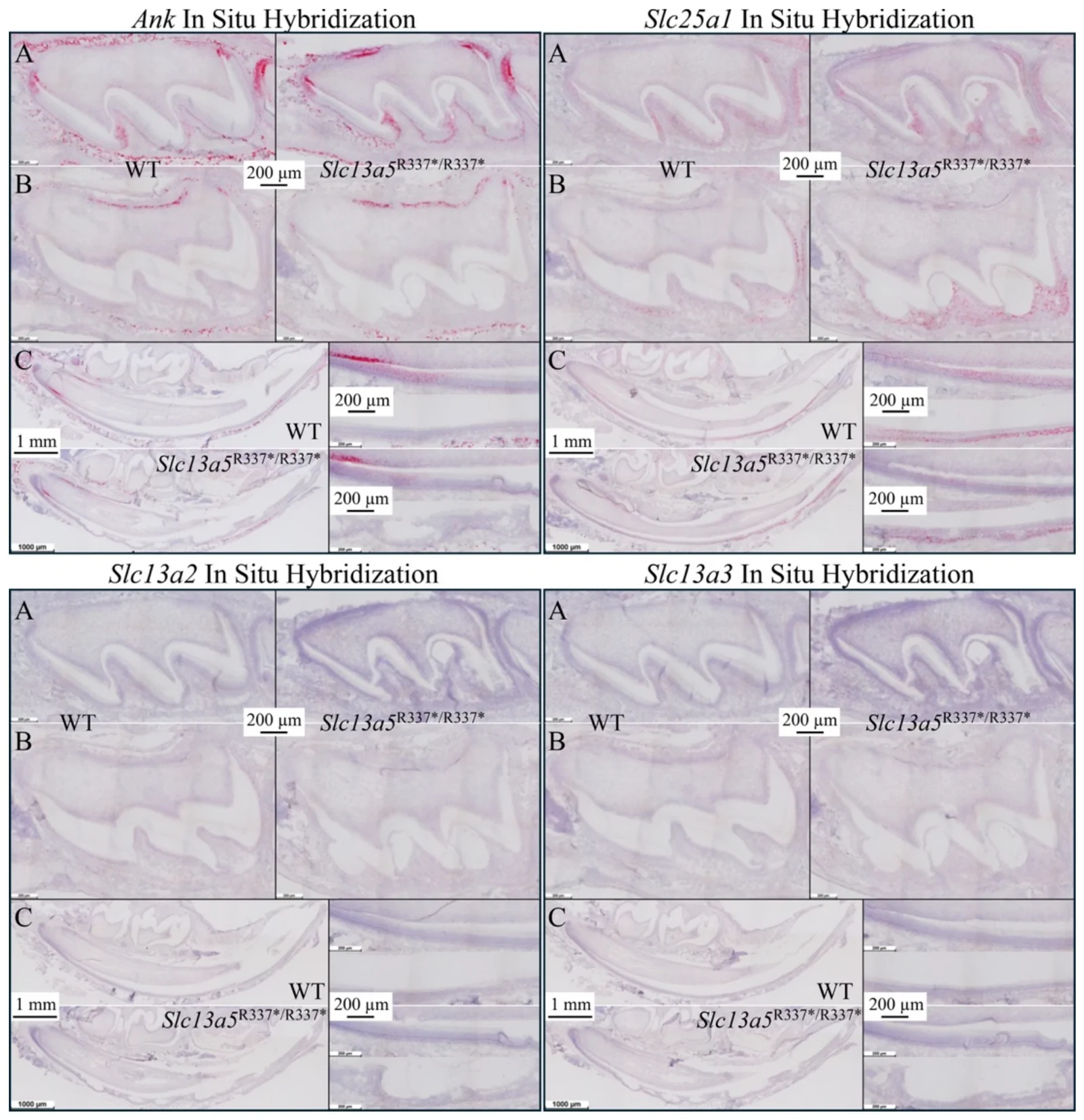
Citrate transporter expression in days 4 and 12 *Slc13a5*^+/+^ and *Slc13a5*^R337*/R337*^ mouse teeth. Sections (5 µm) of mouse maxillary molars at D4 (**A**,**B**) and mandibular incisors at D12 (**C**) were stained with oligo probes for *Ank* (**top left**), *Slc25a1* (**top right**), *Slc13a2* (**lower left**), and *Slc13a3* (**lower right**). While *Slc13a5* (encoding NaCT) expression increased as the ameloblasts and odontoblasts became differentiated, *Ank* expression was limited to pre-ameloblasts and pre-odontoblasts. The expression pattern of *Slc25a1* (encoding a citrate exporter on the mitochondrial membrane) was similar to the expression pattern of *Ank*. Expression of *Slc13a2* (NaDC1) and *Slc13a3* (NaDC3), two dicarboxylic transporters, was not detectable. The absence of functional NaCT did not alter the expression levels of other citrate transporters in the developing mouse teeth. Key: Ob, osteoblasts of the alveolar bone; Od, odontoblasts; Am, ameloblasts. Scale bars in (**A**,**B**) panels are 200 µm, and scale bars in panel (**C**) lower-magnification photos are 1000 µm, and higher-magnification photos are 200 µm. There were two molar samples and three incisor samples from each genotype in these experiments.

*Ank* expression did not appear to be altered in *Slc13a5*^R337*/R337*^ molars or incisors, suggesting that loss of functional NaCT does not affect its expression. *Slc25a1* expression in developing tooth organs was most apparent in the enamel-free zone and stratum intermedium, with increased expression in the stratum intermedium observed in *Slc13a5*^R337*/R337*^ samples. In comparison, *Slc13a2* and *Slc13a3* expression was not detected in D4 developing molars or D12 mandibular incisors, and the absence of NaCT function did not alter their expression patterns in developing teeth (Figure 14, Figures S9 and S10).

Serum citrate concentrations were significantly increased at D0, D3, D5, D12, 7 weeks, and 35 weeks in the absence of functional NaCT transporter (Figure S11). Notably, D0 *Slc13a5*^R337*/R337*^ mice exhibited markedly elevated serum citrate concentrations, suggesting a potential maternal effect, as these mice were offspring of homozygous null breeding pairs. After D0, serum citrate concentrations decreased at D3 but subsequently continued to rise in both wild-type and *Slc13a5*^R337*/R337*^ mice at later time points. This trend is consistent with a previous report by Dirckx et al., 2022 [28]. In addition, citrate content in the femur and tibia was significantly higher in 7-week- and 35-week-old *Slc13a5*^R337*/R337*^ mice than in wild-type controls (Figure S12A). No significant sex-based differences in long-bone citrate content were observed in either wild-type or *Slc13a5*^R337*/R337*^ mice at either time point (Figure S12B,C).

Comparing the developing teeth of wild-type and *Slc13a5*^R337*/R337*^ D5 and D12 mice using µCT analyses revealed decreased enamel/dentin matrix deposition in *Slc13a5*^R337*/R337*^ samples (Figure S13). Although citrate content was increased in the D5 and D12 molar matrix of *Slc13a5*^R337*/R337*^ mice compared with wild-type samples, calcium content was significantly decreased (Figure S14A,B). 

**Figure 15:**
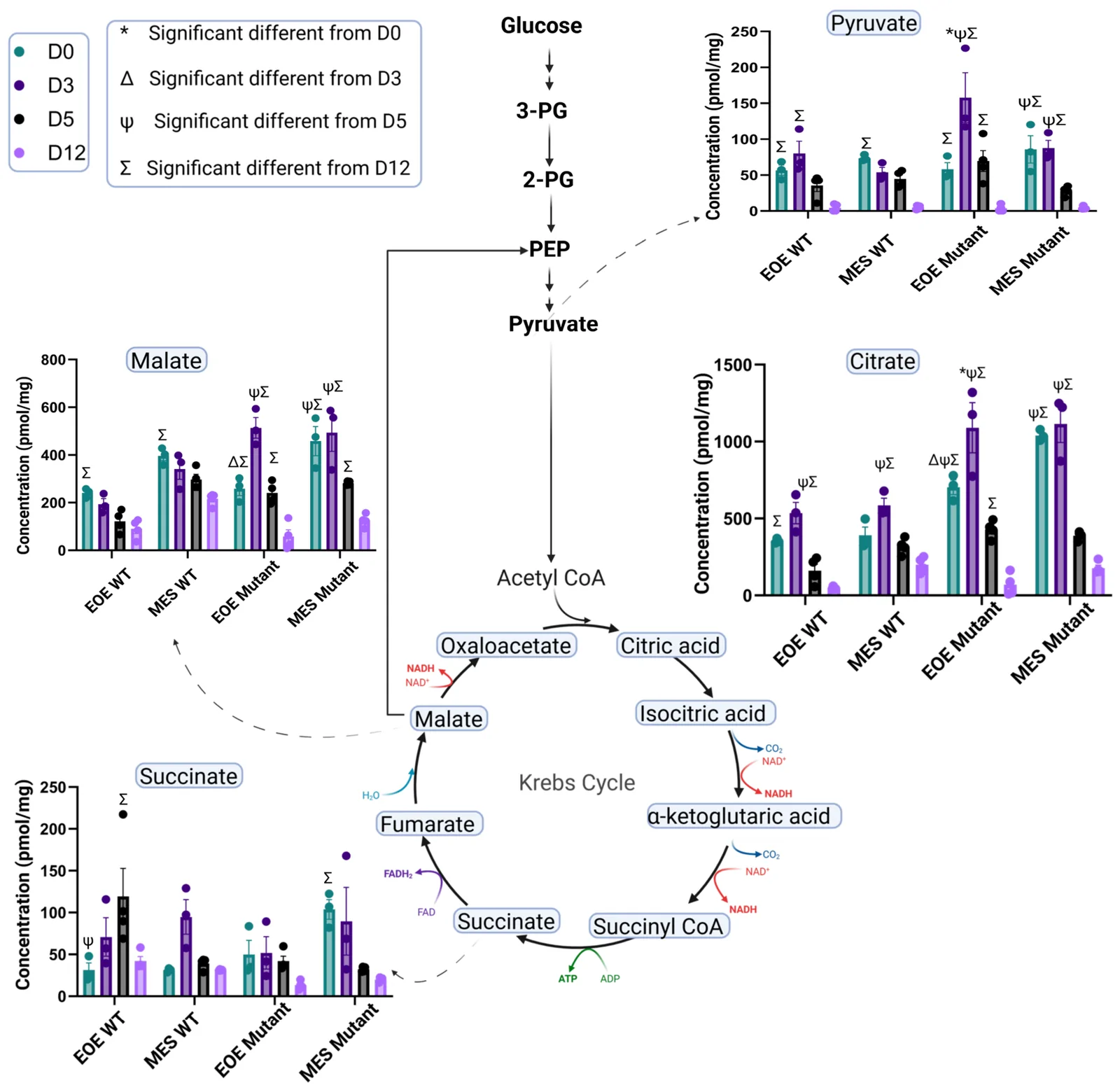
Intracellular concentrations of TCA cycle metabolites vary with *days* in development. Enamel organ epithelium and pulpal mesenchyme from wild-type and *Slc13a5*^R337*/R337*^ mouse molars showed increased accumulation of intracellular citrate and its export to the cytosol via the citrate–malate shuttle, which peaked at day 3. No significant alterations in the TCA cycle activities were observed between the wild-type and *Slc13a5*^R337*/R337*^ mice at these time points. Statistical significance was determined using two-way ANOVA, with *p* < 0.05 considered significant. At each time point, there were three samples from each genotype; each sample contained tissues from 12 molars of 3 mice.

**Figure 16:**
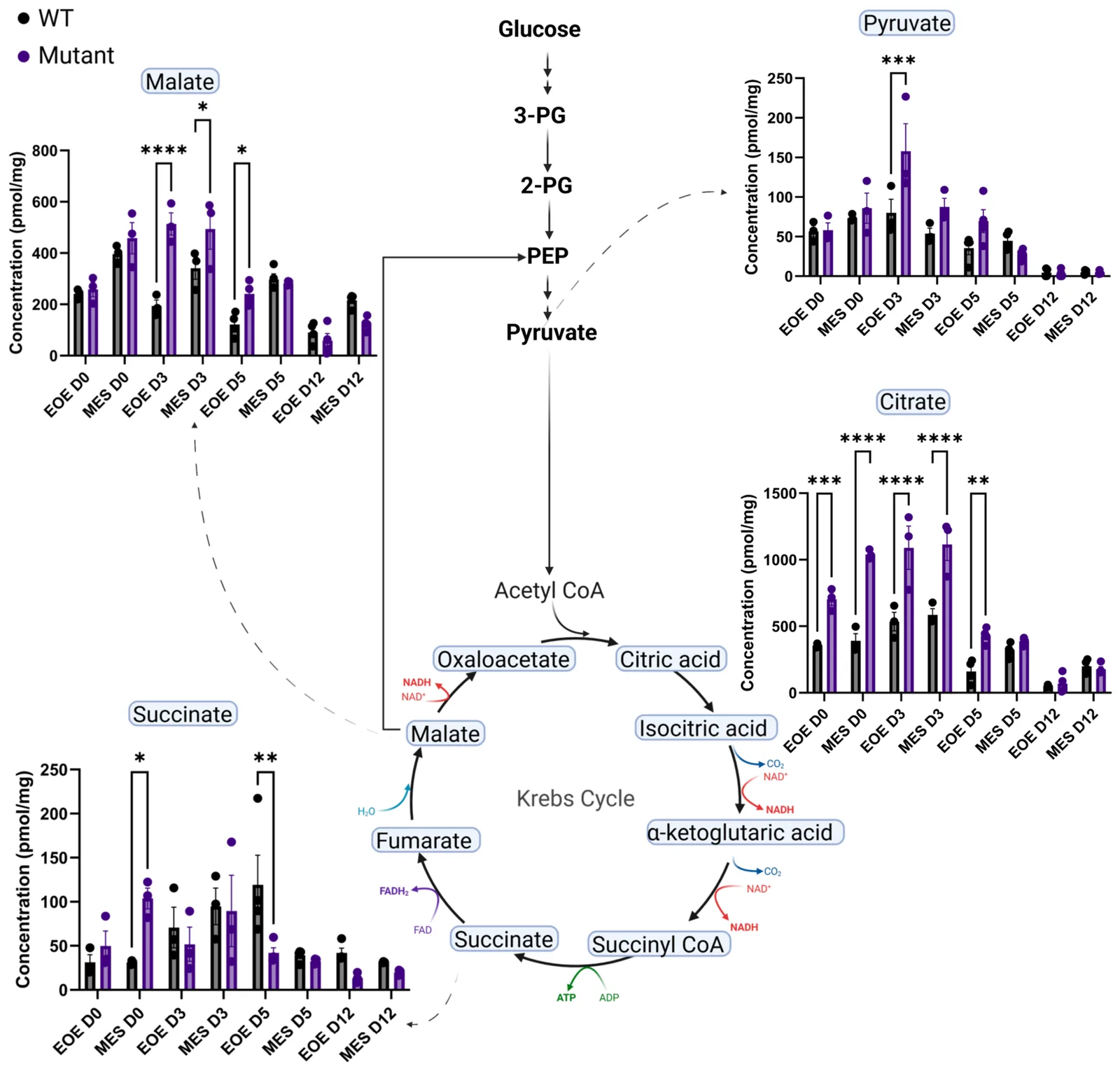
Intracellular concentrations of TCA cycle metabolites vary with *genotypes*. Compared to the wild-type, *Slc13a5*^R337*/R337*^ mouse molars showed accumulation of intracellular citrate and its export to the cytosol via the citrate–malate shuttle in both enamel organ epithelium and pulpal mesenchyme. However, no significant changes in the TCA cycle activities were observed between the wild-type and the *Slc13a5*^R337*/R337*^ mouse molars. Statistical significance was determined using two-way ANOVA, with *p* < 0.05 considered significant. Key: increasing significance (* *p* < 0.05, ** *p* < 0.01, *** *p* < 0.001, **** p < 0.0001). At each time point, there were three samples from each genotype; each sample contained tissues from 12 molars of 3 mice.

**Figure 17:**
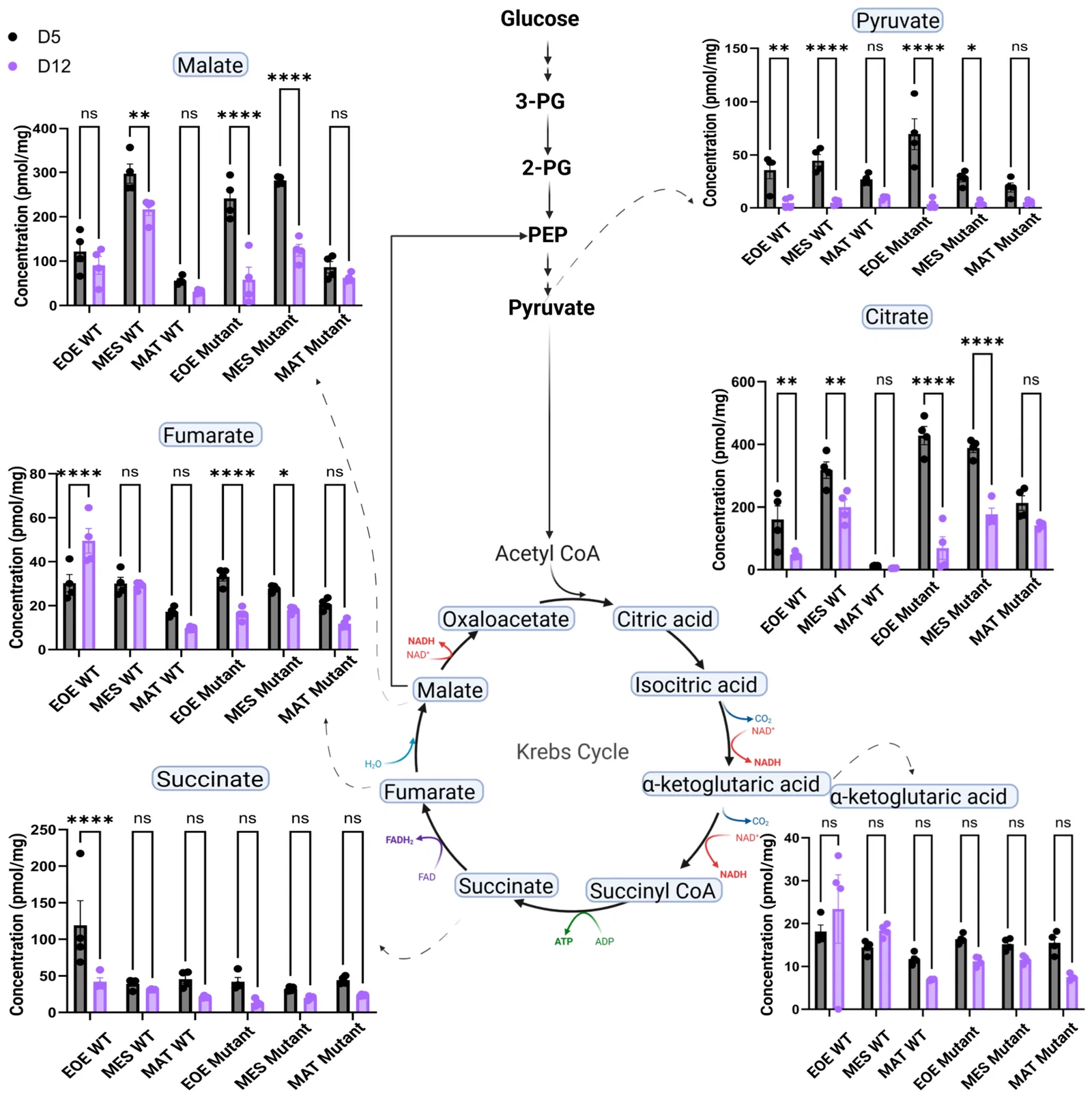
Intracellular concentrations of TCA cycle metabolites vary with *days* in development. Enamel organ epithelium and pulpal mesenchyme from wild-type and the *Slc13a5*^R337*/R337*^ mouse molars showed decreased accumulation of intracellular citrate and its export to the cytosol via the citrate–malate shuttle as they progressed from day 5 to day 12 of development. The matrix sample showed no significant differences between day 5 and day 12. No significant alterations in the TCA cycle activities were observed. Statistical significance was determined using two-way ANOVA, with *p* < 0.05 considered significant. Key: not significant (ns), increasing significance (* *p* < 0.05, ** *p* < 0.01, **** *p* < 0.0001). At each time point, there were three samples from each genotype; each sample contained tissues from 12 molars of 3 mice.

**Figure 18:**
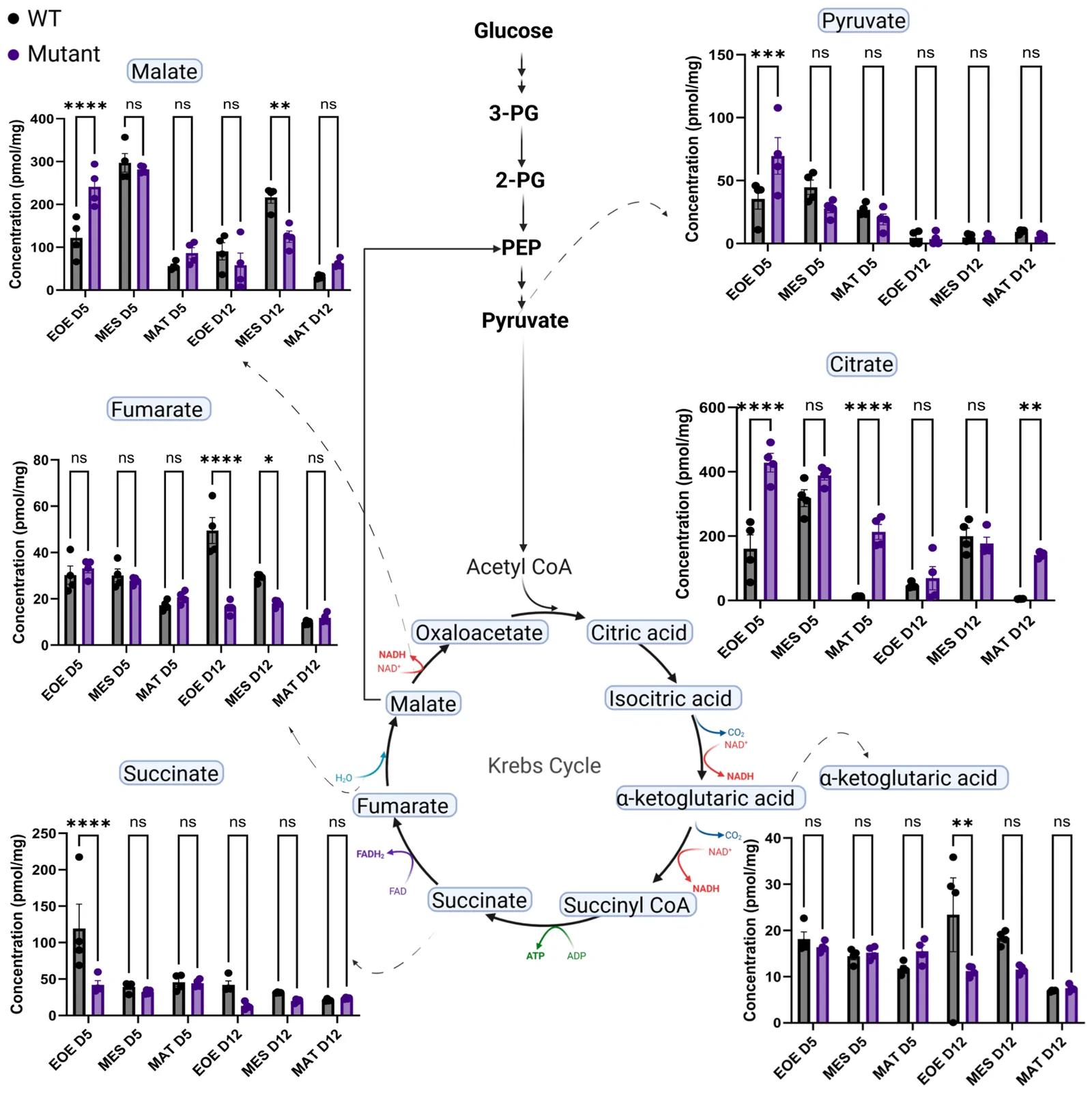
Intracellular concentrations of TCA cycle metabolites vary with *genotypes*. Compared to wild-type, the *Slc13a5*^R337*/R337*^ mouse molars showed accumulation of intracellular citrate and its export into the cytosol via the citrate–malate shuttle in both enamel organ epithelium, pulpal mesenchyme, and the matrix. However, no significant changes in the TCA cycle activities were observed. Statistical significance was determined using two-way ANOVA, with *p* < 0.05 considered significant. Key: not significant (ns), increasing significance (* *p* < 0.05, ** *p* < 0.01, *** *p* < 0.001, **** *p* < 0.0001). At each time point, there were three samples from each genotype; each sample contained tissues from 12 molars of 3 mice.

TCA cycle metabolite analyses of EOE showed that both wild-type (NaCT-intact) and *Slc13a5*^R337*/R337*^ cells exhibited increased intracellular citrate and malate levels from day 0 to day 3 of development (Figure 15 and Figure 16). This increase was more pronounced in *Slc13a5*^R337*/R337*^ cells than in wild-type cells. Pulpal mesenchyme (MES) cells showed a similar pattern. Notably, intracellular citrate and malate concentrations decreased as development progressed from day 3 to day 5, with the lowest concentrations detected at day 12 in both EOE and MES cells. Although trends in other TCA cycle metabolites suggest active fumarate–malate interconversion (Figure 17 and Figure 18), the overall metabolite profile suggests that EOE and MES cells upregulate citrate–malate shuttle activity to buffer the increased demand for citrate during early development, from day 0 to day 3. Moreover, loss of NaCT function appears to further enhance citrate–malate shuttle activity in EOE and MES cells, increasing the cytosolic citrate pool.

No significant changes were observed in the levels of other TCA cycle metabolites when comparisons were made across developmental stages or between wild-type and *Slc13a5*^R337*/R337*^ samples. Thus, although citrate and malate levels increased, the overall TCA cycle metabolite profile did not indicate a major change in TCA cycle activity. Accordingly, we did not expect a substantial shift in cellular bioenergetics attributable to TCA cycle-associated ATP production. In D5 EOE cells, branched-chain amino acid (BCAA) levels were increased in *Slc13a5*^R337*/R337*^ samples compared with wild-type controls, although these differences became less pronounced by D12 (Figures S15 and S16). Similarly, with the exception of valine, leucine and isoleucine levels were increased in D5 *Slc13a5*^R337*/R337*^ MES cells compared with wild-type MES cells, with the differences again becoming less evident by D12 (Figures S15 and S16). Matrix samples showed no clear pattern of BCAA dependence across developmental stage or genotype (Figures S15 and S16).

Intracellular levels of proline, a metabolite previously implicated in tooth and bone development [29,30], were increased in *Slc13a5*^R337*/R337*^ EOE and MES cells compared with wild-type cells (Figure S17). However, proline levels decreased as tooth development progressed from D5 to D12, particularly in the mutant group (Figures S17 and S18).

Postnatal D12 and D14 wild-type, *Slc13a5*^R337*/R337*^, and *Odaph*^C41*/C41*^ mice were used to assess potential pH variation during the post-secretory, transition, and early maturation stages of amelogenesis. ODAPH is required for the successful transition to the maturation stage of amelogenesis [31], and loss of ODAPH function causes hypomaturation amelogenesis imperfecta (OMIM #614832). The *Odaph*^C41*/C41*^ mouse model exhibits defects during post-secretory transition (PST) and maturation stages of amelogenesis, whereas the *Slc13a5*^R337*/R337*^ model presents with a secretory-stage defect. Methyl red staining revealed genotype-dependent differences in enamel surface acidification at both D12 and D14, with consistent patterns observed in both male and female mice.

At D12 (Figure S19A), wild-type incisors from both sexes exhibited strong, continuous orange–red staining, indicating proton release likely associated with enamel mineral deposition. In contrast, *Slc13a5*^R337*/R337*^ incisors showed little to no detectable methyl red staining in either sex, suggesting an absence of enamel surface acidification and a severe defect in both mineral deposition and pH regulation. *Odaph*^C41*/C41*^ incisors displayed irregular, patchy, and discontinuous staining in both sexes, consistent with disrupted surface acidification associated with a maturation-stage defect.

At D14 (Figure S19B), when enamel maturation is more advanced, these genotype-specific patterns persisted. Wild-type incisors continued to show well-defined acidic staining, a by-product of successful enamel deposition. *Slc13a5*^R337*/R337*^ incisors again lacked detectable staining, consistent with failure to advance mineralization. *Odaph*^C41*/C41*^ incisors maintained a patchy, non-uniform staining pattern, further indicating impaired maturation-stage mineralization.

## 3. Discussion

The CRISPR/Cas9 gene-editing strategy enabled targeted knock-in of a single nucleotide substitution, c.1009C>T, in *Slc13a5* exon 7, with the predicted cut site located three nucleotides downstream of the arginine 337 codon, CGA. The resulting *Slc13a5*^+/R337*^ mice exhibited normal tooth development and mineralization. In contrast, *Slc13a5*^R337*/R337*^ mouse incisors displayed enlarged dentinal tubules and lacked normal enamel formation, a phenotype homologous to hypoplastic amelogenesis imperfecta in humans with DEE-25. Notably, these mice showed no apparent physical, neurological, or reproductive abnormalities, although litter size was reduced compared with wild-type mice. Genotype distributions from heterozygous breeding pairs were consistent with expected Mendelian ratios.

### 3.1. Secretory Stage Defect Underlying Amelogenesis Imperfecta in Slc13a5^R337*/R337*^ Mouse

Hypoplastic enamel in *Slc13a5*^R337*/R337*^ mice was evident immediately upon incisor eruption and was characterized by a thinner, chalky white tooth structure with blunted wear facets. The contours of both incisors and molars reflected the absence of enamel, resulting in rapid attrition of occlusal surfaces following eruption. The severity of this enamel defect was unexpected. Unlike enamelin, *Enam*, and ameloblastin, *Ambn*, null mice, in which ameloblast pathology occurs rapidly after dentin mineralization and coincides with failure of enamel ribbon formation, *Slc13a5*^R337*/R337*^ mice exhibited an immediate failure of enamel mineralization at the onset of the secretory stage, while the ameloblasts showed increasing pathology only at the late secretory stage. Histological analyses revealed that the ameloblast layer was polarized during the secretory stage but became disorganized and depolarized during the late secretory stage, ultimately losing its sheet-like architecture. 

As expected, dentin and enamel hardness were significantly decreased in *Slc13a5*^R337*/R337*^ mouse teeth. However, the comparable hardness of alveolar bone among wild-type, *Slc13a5*^+/R337*^, and *Slc13a5*^R337*/R337*^ mice was unexpected. This finding is consistent with the report by Dirckx et al., 2022, in which NaCT knockout mice showed no overt bone phenotype until osteoporosis developed in late adulthood [28].

The most significant pathological changes leading to impaired enamel formation were observed in secretory-stage ameloblasts. The abnormalities seen during the maturation stage likely represent secondary consequences of the primary defects that occurred during the secretory stage. Immunohistochemistry for amelogenin, enamelin, and the lysosomal marker LAMP1 revealed that secretory-stage ameloblasts in *Slc13a5*^R337*/R337*^ mice failed to develop Tomes’ processes, and the ameloblast layer detached from the developing matrix surface. Although amelogenin and enamelin were produced at levels comparable to those in wild-type samples, these matrix proteins accumulated within the supranuclear cytoplasm rather than being properly secreted. During the late secretory stage, disorganized ameloblasts lost cell–cell contacts, leading to diffusion of matrix proteins into the surrounding tissue. Increased lysosomal activity was evident by LAMP1 immunostaining throughout secretory-stage development. Ultimately, impaired secretion of enamel matrix proteins disrupted mineral formation, and their intracellular accumulation likely compromised ameloblast viability.

The ameloblast abnormalities observed by immunohistochemistry were consistent with previously reported FIB-SEM analyses [11]. At the interface between mineralizing dentin and the distal membrane of early secretory ameloblasts, *Slc13a5*^R337*/R337*^ samples showed no deposition of enamel matrix, no formation of Tomes’ processes, and no successful enamel mineral ribbon formation. Occasionally, sparse and thin mineral ribbons were initially observed, but these disappeared shortly thereafter (see Figure 4 in Simmer et al., 2021) [11]. In contrast, wild-type samples at comparable developmental stages consistently showed Tomes’ processes, matrix accumulation, and enamel mineral ribbons organized into rod and interrod bundles. Although the organization of mitochondria, Golgi complexes, and rough endoplasmic reticulum within ameloblasts was comparable between wild-type and *Slc13a5*^R337*/R337*^ samples, *Slc13a5*^R337*/R337*^ ameloblasts contained markedly increased numbers of lysosomes and vacuoles. Using artificial coloring to distinguish and quantify major cellular organelles in ImageJ v1.53, we detected a significant increase in primary and secondary lysosomes at Level 1.5, corresponding to an early phase of the secretory stage of amelogenesis. Disorganization of the ameloblast layer became evident around Level 2.5 and persisted through Level 3.0, the end of the secretory stage. These findings suggest that loss of NaCT function directly affects enamel mineral formation or stability. The failure to initiate enamel formation may subsequently contribute to the observed changes in organelle area fractions.

We asked how ameloblasts respond when *Slc13a5* is mutated and NaCT is rendered nonfunctional. Transcript analyses of relevant citrate transporters suggested that mutant *Slc13a5* expression diminished as amelogenesis progressed into the maturation stage. No apparent changes were observed in *Ank* expression. However, expression of the mitochondrial citrate transporter encoded by *Slc25a1* became evident in the stratum intermedium, but was less abundant in ameloblasts (Figure 14). No detectable changes were observed in *Slc13a2* or *Slc13a3* expression. Based on these findings, loss of NaCT function does not appear to induce a compensatory feedback response that activates alternative citrate transporters in ameloblasts.

The RT-PCR detection of *Ank* expression in D5, D8, and D12 developing first molars requires careful interpretation in light of the in situ hybridization data. During odontogenesis, *Ank* expression did not overlap with *Slc13a5* expression. Instead, the positive RT-PCR results likely reflect amplification of transcripts from tissues included in the dissected tooth organs, such as the cervical loop, the developing epithelial diaphragm at the floor of the pulp chamber, preameloblasts, preodontoblasts, and osteoblasts from the surrounding developing alveolar bone. Thus, the absence of *ANK* expression during the peak secretory stage of amelogenesis makes it unlikely that ANK substitutes for NaCT or mediates citrate transport during the secretory stage ameloblasts.

Much is known about genes encoding the major secreted enamel matrix proteins, including enamelin (*ENAM*), ameloblastin (*AMBN*), amelogenin (*AMELX*), and matrix metalloproteinase 20 (*MMP20*), as well as bicarbonate, HCO_3_^−^, secretion mediated by *SLC4A4*. In contrast, relatively little is known about the equally critical gene *SLC13A5*, which encodes NaCT, a Na^+^/citrate cotransporter. Based on the literature, NaCT mediates citrate influx, and this is likely the case in ameloblasts. In addition, citrate may move along the lateral membranes of ameloblasts through pathways associated with intercellular spaces [30]. The absence of a transporter in ameloblasts to export citrate into the developing enamel, where citrate is known to be present, together with the abundance of NaCT on the lateral membranes and Tomes’ processes of ameloblasts, suggests that citrate normally passes through the semipermeable proximal and distal tight junctions [26]. In the absence of functional NaCT, excessive citrate enters the developing enamel and interferes with the initiation of the enamel ribbon deposition and elongation. NaCT-mediated uptake of citrate into ameloblasts regulates citrate levels entering the forming enamel and provides citrate for metabolic functions.

### 3.2. Citrate Levels and Impacts During Amelogenesis in the Absence of Functional NaCT

Loss of functional NaCT prevents cellular uptake of circulating citrate, which normally can be used for energy production or biosynthesis. Reduced intracellular citrate levels may be compensated, in part, by increased glucose uptake. However, loss of functional NaCT also increases serum citrate concentrations, thereby elevating citrate levels in the interstitial fluid near the proximal ends of odontoblasts and ameloblasts. From this region, citrate can diffuse between odontoblasts into the predentin, which communicates with sites of initial dentin and enamel formation. Both odontoblasts and ameloblasts express SLC13A5 protein, which may normally help reduce citrate concentrations in the fluid surrounding mineralizing dentin and enamel. Loss of this function could therefore compound the effects of elevated systemic citrate. Elevated extracellular citrate at sites of enamel ribbon initiation and elongation may have inhibited critical subsequent processes, such as restricting ion deposition to the tips of incipient mineral ribbons to promote growth along the C-axis and maintaining their association with the ameloblast distal membrane. This disruption may have contributed to the failure of Tomes’ process formation and, consequently, the absence of normal rod and interrod enamel architecture.

In *Slc13a5*^R337*/R337*^ mice, excessive citrate, rather than citrate deficiency, appears to interfere with proper enamel ribbon formation at its onset. The process is disturbed early enough that the Tomes’ process fails to form on the ameloblast distal membrane, preventing the initiation of normal rod and interrod architecture. An intriguing possibility is that the loss of functional NaCT on the distal membrane of *Slc13a5*^R337*/R337*^ ameloblasts prevents the uptake or clearance of excess extracellular citrate. As a result, excessive citrate may bind to incipient enamel ribbons, thereby inhibiting their continued elongation. To investigate this possibility, we assessed TCA cycle metabolites in both the enamel organ epithelium and dental mesenchyme to better understand the metabolic status of these cells.

SLC13A5 and SLC25A1 are two essential citrate transporters that function together to maintain the cytosolic citrate pool, a key component of TCA cycle metabolism [20,32,33]. SLC25A1, also known as the citrate carrier (CIC), facilitates citrate export from the mitochondrial matrix to the cytosol while concurrently returning malate to the mitochondria through the citrate–malate shuttle [33]. In contrast, SLC13A5 imports citrate from the extracellular space into the cytosol [32]. Mutations in SLC13A5 may disrupt this balance and lead to metabolic disturbances.

In EOE and MES samples, citrate levels were significantly increased in the *Slc13a5*^R337*/R337*^ group compared with wild-type controls; however, citrate and malate levels did not change significantly between day 5 and day 12 (Figure 17 and Figure 18). These findings suggest that demand for citrate in the EOE and MES remains relatively stable across these developmental time points; the loss of functional NaCT did not increase reliance on citrate–malate shuttle activity.

Although trends in other TCA cycle metabolites across developmental stages and NaCT status suggest active fumarate–malate interconversion, overall TCA cycle activity appeared largely unchanged. In addition, the absence of broad changes in TCA cycle intermediates suggests that increased citrate and malate levels had minimal impact on cellular bioenergetics derived from TCA cycle-associated ATP production. This finding is consistent with observations that extracellular citrate makes appreciable contributions to biosynthesis primarily under specific stress conditions, such as hypoxia or glutamine restriction [34]. In other words, citrate may function less as a major biosynthetic substrate under normal conditions and more as a metabolic resource during distinct cellular stress [34]. Nevertheless, cytosolic citrate remains a critical precursor for de novo lipogenesis because it provides the acetyl-CoA required for fatty acid and cholesterol synthesis [35]. Consequently, impaired citrate availability can limit the cytosolic acetyl-CoA pool required for membrane biosynthesis, lipid production, and cell growth [33].

Isoleucine, leucine, and valine are essential branched-chain amino acids (BCAAs) that can generate intermediates feeding into the TCA cycle and other biosynthetic pathways to support cellular development and bioenergetic needs [20]. Leucine can generate acetyl-CoA, valine can generate succinyl-CoA, and isoleucine can generate both acetyl-CoA and succinyl-CoA. Overall, these findings suggest that EOE and MES cells may indirectly supplement their cytosolic citrate pool by increasing intracellular BCAAs availability at day 5 of development, with reduced reliance on BCAAs as development progresses to day 12. Impaired cytosolic citrate flux may substantially disrupt the biosynthesis of critical molecules, including fatty acids, nucleotides, and amino acids, which are essential for cellular growth and differentiation [14]. We therefore speculate that defective Tomes’ process formation, detachment of ameloblasts from the matrix surface, and intracellular accumulation of matrix proteins that would normally be secreted may reflect insufficient cytosolic citrate availability.

The absence of functional NaCT significantly alters the microenvironment in which calcium phosphate phases are deposited. Methyl red staining demonstrated that wild-type enamel maintains pH cycling from the secretory through maturation stages. In contrast, the *Slc13a5*^R337*/R337*^ mutation resulted in defective acid regulation, whereas the *Odaph*^C41*/C41*^ mutation produced a distinct disruption characterized by patchy acidic microenvironments.

It is certain from a strong in situ hybridization signal in *Slc13a5*^R337*/R337*^ incisors, which only express mRNA translating truncated SLC13A5 protein, so that many *Slc13a5*^R337*/R337*^ transcripts escape nonsense-mediated decay (NMD). If NMD of these transcripts was efficient, no positive signal for *Slc13a5*^R337*/R337*^ incisors and molars would be observed. It is reasonable to assume that a significant amount of mutant *Slc13a5*^R337*^ transcripts are synthesized and transcribed into protein in the heterozygous *Slc13a5*^+/R337*^ ameloblasts. Despite this, the heterozygous *Slc13a5*^+/R337*^ molars and incisors show no enamel malformations. This strongly suggests that the truncated *Slc13a5*^R337*^ protein is either not toxic or is efficiently degraded. These findings make it highly likely that the pathology observed in the ameloblast layer of *Slc13a5*^R337*/R337*^ molars and incisors is the result of ameloblasts being unable to deposit and extend enamel mineral ribbons to generate an enamel layer without a functional SLC13A5 protein to transport citrate.

## 4. Materials and Methods

### 4.1. Animal Model and Genotyping

Mice were maintained in a pathogen-free facility under a controlled light–dark cycle and provided standard rodent chow. When single housing was required, appropriate enrichment materials were provided. Sample sizes were determined based on prior studies [28,36,37] and the relevant literature [10,31,38,39]. The specific sample size of each experiment is described in the figure legend, except for the Methyl Red study. The sample size of the Methyl Red study is described in the experimental procedures section.

Mouse solute carrier family 13 member 5 [*Mus musculus*] (sodium-dependent citrate transporter) is designated as *Slc13a5* or NaC2/NaCT, GRCm39: ENSMUSG00000020805, GCF_000001635.27, NC_000077.7, Ch11: 72,132,816.72,158,092, reverse strand. The coding sequence of *Slc13a5*, sgRNA, and DNA oligo donor sequences were analyzed and designed. The specific strategy was to use Cas9 to induce a double-strand chromosome break in exon 7 near codon 337, followed by a singlestranded oligonucleotide donor to change codon 337 from Arg (CGA) to Opal (TGA). Codon 337 was 59 bp upstream of an exon–intron boundary and was expected to cause nonsense-mediated decay of mRNA from transcript *Slc13a5* [40]. The specific sequence of sgRNA CTACTTGTC ATCCTGTGGTTCTCC**CGA**GACCCCGGCTTCATGCCTGGCTGGCTGTCATTCGC targeted exon 7 with a predicted cut site located three nucleotides downstream of the Arginine^337^ codon **CGA** (Figure 1). The DNA oligo donor was designed to introduce silent sequence changes and c.1009C>T, producing a stop gain TGGGGTCCTTGAGCTACCCTGAATGCAACGTGCTCTTTTGCTTCACCCTACTGTCATCCTGTGGTTCagC**t**GAGAtCCtGGaTTtATGC CTGGCTGGCTGGCTGTCATTCGCCTGGGTCGAGGGAAACACCGTGTAAGTCGATGCAAAG. Genotyping primers included wild-type primer set *Slc13a5* FOR1: 5′-CCACTTCTCCCACAAGTCG-3′ and *Slc13a5* REV1: 5′-CCCTCGACCCAGGCGAATG-3′ generating an amplicon of 270 bp and mutant primer set of *Slc13a5*FOR1: 5′-CCACTTCTCCCACAAGTCG-3′ and *Mut*Rev1: 5′-GGCATAAATCCAGGAGGATCTCAGC-3′, producing an amplicon of 240 bp. The genotyping strategy was further optimized. Primer set *Slc13a5*-Ex7-WT-F: 5′-CCGAGACCCCGGCTTCA-3′ and *SLC13A5*-Ex7-WT-R: 5′-CCCCAGCCTACACTGCTCCA-3′ were used to generate a 634 bp amplicon from the wild-type allele. Primer set *Slc13a5*-Ex7-MU-F: 5′-GGTTCAGCTGAGATCCTGGATTTAT-3′ and *Slc13a5*-Ex7-MU-R: 5′-CATACACGCCCTTCCTCCAA-3′ were used to generate a 420 bp amplicon from the mutant allele (Figure 2). PCR conditions included initial denaturation at 94 °C for 2 min, then [30 cycles of 94 °C for 30 s (template denaturation), then 62 °C for 30 s (primer annealing), followed by 72 °C for 40 s (primer extension)], 72 °C for 2 min and then hold at 4 °C. Each PCR reaction contained 10 µL of Platinum Hot Start PCR Master Mix (2x) (Invitrogen, Carlsbad, CA, USA), 1 µL of 10 µM primer mix, 100 ng DNA template in 3 µL, and was raised to 20 µL with distilled water. The reactions were run using a GeneAmp PCR System 9700 (Applied Biosystems, Foster City, CA, USA) Thermocycler. Once correctly targeted alleles were identified in G2, all coding exons and intron/exon borders of Slc13a5 were validated using Sanger sequencing using tail gDNA from the G2 breeders.

### 4.2. Transcript Analyses

Developing molars of three mice from time points, postnatal D5, D8, and D12, of both *Slc13a5*^R337*/R337*^ and wild-type mice were dissected for transcript analyses. Specifically, D5 wild-type molars, incisors, and tail tissues were subjected to RT-PCR for the detection of *Ank* and *Gapdh*. Submandibular salivary gland, seminal vesicle and testis were collected from 7-week-old wild-type and *Slc13a5*^R337*/R337*^ mice, while wild-type D5 mandibular first molars were used as a positive control. To detect the transcripts of *Slc13a5,* primer sets *Slc13a5*-1F: 5′-CTGATACCTGACAAGTTTGCCA-3′ and *Slc13a5*-6R: 5′-CTCATGTACAAGCACTGG AGC-3′ covering exons 1 to 6 of transcript variant 1 (TV1) producing an amplicon of 755 bp as well as a primer set *Slc13a5*-8F: 5′-CCAGACTGAGGAAGAAAGGAAA-3′ and *Slc13a5*-11R: 5′-TCCAAGTTAAACATAGCCCGA-3′ covering exons 8 to 11 of TV1 producing an amplicon of 522 bp were used. To detect *Ank* expression, primer set *Ank*-2F: 5′-CCACACCCTGATAGCCTACA-3′ and *Ank*-9R: 5′-CAAAGGCAAAGTCCACTCCA-3′ were used to produce an amplicon of 791 bp in size. A primer set to amplify *Gapdh* F: 5′-AGGCCGGTGCTGAGTATGTC-3′ and *Gapdh* R: 5′-TGCCTGCTTCACCACCTTCT-3′, producing an amplicon of 530 bp, was used as an experimental control. Reaction conditions were denaturation at 94 °C for 2 min, then [25 cycles of 94 °C for 30 s (template denaturation), then 58 °C for 30 s (primer annealing), followed by 72 °C for 50 s (primer extension)], 72 °C for 1 min, and then held at 4 °C. The amplification products were separated on a 2% agarose gel and sequence validated using Sanger sequencing.

### 4.3. Morphological Assessment

To compare developing tooth morphology, 7-week-old wild-type, *Slc13a5*^+/R337*^, and *Slc13a5*^R337*/R337*^ mice, including at least two males and two females per genotype, were anesthetized and perfused with 4% paraformaldehyde (PFA). Hemimandibles were dissected, soft tissues were removed, and the teeth and surrounding bone were cleaned with 1% sodium hypochlorite. Samples were then rinsed, air dried, examined under a Nikon SMZ1000 dissecting microscope (Melville, New York, USA), and photographed using a Nikon DXM1200 digital camera (Melville, New York, USA).

### 4.4. Histology and Immunohistochemistry

Six maxillae from postnatal D0, D4, and D8 mice, and hemimandibles from D12 wild-type, *Slc13a5*^+/R337*^, and *Slc13a5*^R337*/R337*^ mice were dissected and fixed overnight in 4% PFA in PBS at 4 °C. Samples were decalcified at 4 °C in 16.52% disodium ethylenediaminetetraacetic acid (EDTA) pH 7.4, with rotation for 2–5 days, depending on sample age. Decalcified tissues were dehydrated through a graded ethanol series, cleared in xylene, embedded in paraffin, and sectioned at 5 μm thickness using a HistoCore AUTOCUT R microtome (Leica, Deer Park, IL, USA). Sections were mounted on Fisher brand Tissue Path Superfrost Plus Gold microscope slides (Thermo Fisher Scientific, Waltham, MA, USA). Hematoxylin and eosin (H&E) staining was performed as previously described [41]. Images were acquired using a Nikon Eclipse TE300 microscope and photographed with a Nikon DXM1200 digital camera.

Immunohistochemistry was performed on postnatal D12 wild-type and *Slc13a5*^R337*/R337*^ hemimandibles following established protocols [42]. Affinity-purified rabbit anti-mouse amelogenin antibody raised against recombinant mouse amelogenin, rm179, was used at a 1:2000 dilution, and rabbit polyclonal antibodies raised against the synthetic enamelin peptide mEnam223–236 were used at a 1:1000 dilution. Both antibodies were generated by YenZym Antibodies LLC (Brisbane, CA, USA) [43]. Rabbit polyclonal anti-LAMP1 antibody (ab24170, Abcam, Waltham, MA< USA ) was used at a 1:1000 dilution. Alexa Fluor Plus 594 goat anti-rabbit IgG, H + L, secondary antibody, 1:1500 (A32740, Invitrogen, Carlsbad, CA, USA) was used for detection. A FITC-conjugated mouse monoclonal antibody against β-actin (ab6277, Abcam) was used at a 1:2000 dilution. Sections were counterstained with DAPI (P-36931, Invitrogen) and mounted with Aqueous Mounting Solution (Invitrogen, Carlsbad, CA, USA). Images were acquired using a Leica STELLARIS 8 confocal microscope (Buffalo Grove, IL, USA) equipped with HyD detectors at the Imaging Laboratory of the University of Michigan Diabetes Research Center.

### 4.5. In Situ Hybridization

Postnatal maxillary molars from D0, D4, D8, and D11 mice and mandibular incisors from D12 wild-type, *Slc13a5*^+/R337*^, and *Slc13a5*^R337*/R337*^ mice were collected, with two samples obtained from each genotype at each time point. Positive and negative controls were included in all experiments, which were performed according to an established protocol [41]. Experiments were repeated using multiple samples to assess reproducibility. RNAscope^®^ in situ hybridization (ISH) probes were designed and used to target the following transcripts: *Slc13a5* mRNA region 41–1023 bp (Cat# 533391, NM_001004148.4, Advanced Cell Diagnostics, Newark, CA, USA); *Ank* mRNA region 285–1329 bp (Cat# 441181, NM_020332.4); *Slc25a1* (Cat# 545261, NM_153150.2); *Slc13a2* (Cat# 452991, NM_022411.3); and *Slc13a3* (Cat# 461041, NM_054055.2). Probe-*dapB* (Cat# 310043, EF191515) was used as the negative control. These riboprobes were designed to amplify target-specific signals while minimizing background from nonspecific hybridization [44].

### 4.6. Backscattered Scanning Electron Microscopy (bSEM)

Two- and seven-week-old wild-type, *Slc13a5*^+/R337*^, and *Slc13a5*^R337*/R337*^ mice, with three mice per genotype at each time point, were anesthetized and perfused with 4% PFA. Hemimandibles were carefully dissected, cleared of soft tissue, and prepared for surface scanning. Additional hemimandibles from six 7-week-old mice of each genotype were dehydrated through an acetone series, embedded in epoxy, cross-sectioned at 1 mm increments along the anterior–posterior axis, and examined by backscattered scanning electron microscopy (bSEM) at each increment. Samples were imaged using an Amray EF 1910 scanning electron microscope operating at an accelerating voltage of 5 kV at the Michigan Center for Materials Characterization, University of Michigan. At least three samples, including both males and females, were included in each experiment.

### 4.7. Micro-Indentation Hardness Testing

Mandibular incisors from 7-week-old littermates of each genotype, wild-type, *Slc13a5*^+/R337*^, and *Slc13a5*^R337*/R337*^, were subjected to microhardness testing. Hemimandibles were dissected free of soft tissue and dehydrated through a graded acetone series, 30%, 50%, 70%, 80%, 90%, 95%, and 100% twice, for 30 min each. Samples were then embedded in Epon resin, EMbed 812 (cat#14120; Electron Microscopy Sciences, Hatfield, PA, USA). After resin polymerization at 60 °C, the incisors were cross-sectioned using a Model 650 low-speed diamond wheel saw (South Bay Technology, Inc., San Clemente, CA, USA) at the level near the alveolar bone crest where the incisor erupts into the oral cavity, approximately 8 mm from the incisor apex. The sectioned hemimandibles were re-embedded in Castolite AC (Eager Polymers, Chicago, IL, USA), using 25 mm SteriForm molds (Struers Inc., Westlake, OH, USA), with the cut surface facing downward, and allowed to harden overnight. The exposed cross-sectional surfaces of the mandibular incisors were polished sequentially using a Syntron vibratory polisher with 400-, 800-, and 1200-grit waterproof silicon carbide papers, followed by polishing with 1 μm diamond polishing paste (South Bay Technology, Inc., San Clemente, CA, USA). Nanohardness testing was conducted using a Hysitron 950 Triboindenter with a nanoDMA transducer and Berkovich probe at the University of Michigan Center for Materials Characterization. The nanoindentations were analyzed using Triboscan 9 software.

### 4.8. Focused Ion Beam–Scanning Electron Microscopy

FIB-SEM protocols have been established and described previously [10]. Briefly, three seven-week-old wild-type and three *Slc13a5*^R337*/R337*^ mice were anesthetized and perfused with 2.5% glutaraldehyde in 0.08 M sodium cacodylate buffer (pH 7.3) with 0.05% calcium chloride. Samples were post-fixed in the same fixative for 4–6 h and then changed to 0.1 M sodium cacodylate buffer (pH 7.3). Hemi-mandibles were washed several times with 0.1 M sodium cacodylate buffer, lipid-stained with 1% reduced osmium tetroxide for 2 h, dehydrated using an acetone gradient, and cured in pure epoxy. Each incisor was cut into eight 1 mm thick cross-sectional slices. The cross-sectional slices were rotated 90° and glued onto epoxy stubs for ultramicrotome sectioning to expose the longitudinal planes. The longitudinal planes were prepared with glass knives and smoothed using multiple etchings with the gallium ion beam. Imaging was done by SEM at 5000× to 35,000× magnifications using a FEI Helios Nanolab 660 DualBeam Focused Ion Beam–Scanning Electron Microscope as described previously (Facility for Electron Microscopy Research, McGill University, Montreal, QC, Canada). Normal enamel formation initiates at level 1.0 and continues to level 3.0, where the maturation stage begins. During the secretory stage, dentin has higher mineral density than enamel. Pseudo-coloring of the FIB-SEM images was carried out using NIH ImageJ v1.53 software for the purpose of identifying and quantifying the relative volume of various intracellular organelles in ameloblasts from seven-week-old mandibular incisors at levels 0.8, 1.0, 1.5, 2.0, 2.5, and 3.0 of wild-type and *Slc13a5*^R337*/R337*^ mice. Seven organelles/structures were colored on FIB-SEM images. After being colored manually, the % volume of the ameloblast layer represented by a particular subcellular organelle was measured and plotted.

### 4.9. Micro-Computed Tomography (CT) Scanning

Postnatal D5 and D12 wild-type and *Slc13a5*^R337*/R337*^ mice, three per genotype at each time point, were collected for this analysis. Hemimandibles were dissected, cleaned, washed in 1× PBS, placed in vials, and immersed in 70% ethanol. At each time point, four hemimandibles were scanned using a SkyScan 1172 system (Bruker SkyScan, Aartselaar, Belgium). Each specimen was positioned in the scanning tube with the ramus oriented inferiorly and the incisal tip oriented superiorly, then stabilized with agarose. The tube was sealed with Parafilm (American National Can Company, Greenwich, CT, USA). Samples were scanned at 60 kV, 167 µA beam intensity, 0.7° rotation step, 4-frame averaging, 2000 × 1336 CCD resolution, 700 ms exposure time, and 5 µm voxel size. The scan time was approximately 35 min per hemimandible. DICOM files were generated from the raw data, exported, and analyzed according to a previously established protocol [45].

### 4.10. Citrate and Calcium Assays

Adult wild-type and *Slc13a5*^R337*/R337*^ mice at 7 and 35 weeks of age were used to assess citrate levels in serum and long bones, specifically femurs and tibiae. Both male and female mice were collected and analyzed at each time point. Following euthanasia under isoflurane anesthesia, blood samples were collected first. Tibiae and femurs were then carefully dissected free of surrounding soft tissues. Both ends of each long bone were excised, and bone marrow was flushed with cold PBS before the bones were freeze-dried overnight. Each bone sample was subsequently weighed, ground into powder, and suspended in 500 µL of 1 N HCl. After brief centrifugation, the supernatant was collected, and 20 µL of PBS-diluted supernatant was used for citrate analysis.

For adult mice at 7 and 35 weeks, deep anesthesia was induced with isoflurane, and blood was collected by cardiac puncture into Microtainer tubes (Becton, Dickinson and Company, Franklin Lakes, NJ, USA). For D0–D5 pups, blood was collected immediately after decapitation. D12 pups were deeply anesthetized with isoflurane before decapitation and blood collection. Because of the limited blood volume obtainable from neonatal and juvenile mice, blood samples from three mice were pooled. At least three pooled samples, representing nine mice per genotype at each time point, were collected. Samples were allowed to clot at room temperature for 30 min and then centrifuged at 14,500 rpm for 3 min. The serum fraction was collected by pipette and stored at −80 °C until analysis. A 40 µL aliquot from each serum sample was used for citrate assay.

Serum samples were deproteinated using Amicon Ultra-0.5 10 kDa spin filters (Millipore Sigma, Burlington, MA, USA). Each sample was diluted 1.5- to 2-fold to reach the minimum required volume and appropriate concentration range for the citrate assay. Serum citrate concentrations were measured using the EnzyChrom Citrate Assay Kit (BioAssay Systems, Corporate Place, Hayward, CA, USA), according to the colorimetric protocol. Briefly, citrate standards were prepared at 400, 240, 120, and 0 µM in ddH_2_O. Each standard, 20 µL, was mixed with 80 µL of working reagent containing citrate lyase (CL) enzyme in a clear 96-well plate. The working reagent consisted of 85 µL developer, 1 µL CL enzyme, 1 µL ornithine decarboxylase enzyme, and 1 µL dye reagent. For each serum sample, two 20 µL aliquots were loaded into separate wells. One aliquot was mixed with 80 µL of working reagent containing CL enzyme, whereas the second aliquot was mixed with 80 µL of working reagent without CL enzyme and served as the sample blank. The blank reading represented potential background signal generated by non-citrate serum components. The corrected sample value was calculated by subtracting the blank OD value from the sample OD value at 570 nm. Plates were incubated for 15 min at room temperature protected from light, and absorbance at OD570 nm was measured using a BioTek Synergy NEO microplate reader (Agilent, Santa Clara, CA, USA). A standard curve was generated from the citrate standards, with a linear detection range of 1–400 µM. Citrate concentration was calculated by dividing the corrected OD value by the slope of the standard curve and multiplying by the dilution factor.

### 4.11. Citrate Analysis of Developing Tooth Tissues

Enamel organ epithelium (EOE), enamel/dentin matrix (MAT), and pulpal mesenchyme (MES) were collected separately from postnatal D0, D3, D5, and D12 wild-type and *Slc13a5*^R337*/R337*^ mice. Three pooled samples, representing a total of nine mice, were collected for each genotype and time point. Blood samples were collected before tissue dissection. Mouse maxillae and mandibles were dissected and kept chilled on an ice pack while first molars were removed under a dissecting microscope. The EOE, enamel/dentin matrix (from D3, D5, and D12 samples), and pulpal mesenchyme were carefully separated and collected into individual Eppendorf tubes. Fresh-frozen molar EOE, matrix, and mesenchyme samples were placed in clean, pre-weighed Eppendorf tubes, weighed, and stored at −80 °C until use. Samples were thoroughly homogenized and centrifuged to collect supernatants for free citrate assays. The precipitate from the matrix homogenate was dissolved in 1 N HCl and centrifuged, and the resulting supernatant was used for bound citrate and calcium assays.

Citrate contents of EOE, MAT, and MES samples were determined using the EnzyChrom Citrate Assay Kit, similar to that described above, according to the manufacturer’s protocol. A standard curve was generated with a linear detection range of 1–40 µM. Fluorescence intensity at λ_ex/em = 530/585 nm was recorded for each sample. Citrate concentration was calculated by direct comparison with the standard curve and normalized to sample dry weight. Data were analyzed using an unpaired Student’s *t*-test.

Calcium content in D5 and D12 enamel/dentin matrix samples was measured using the QuantiChrom Calcium Assay Kit (BioAssay Systems DICA-500, Thermo Fisher Scientific, Waltham, MA, USA), according to the manufacturer’s protocol. Each sample was homogenized and centrifuged to collect the supernatant. A 10 µL aliquot of supernatant from each sample was neutralized with 90 µL of 30 mM Tris-HCl buffer (pH 8.0). Calcium concentration was measured in a 96-well plate using 15 µL of each diluted sample. Optical density at 612 nm was measured using a BioTek Synergy NEO2 microplate reader (Agilent, Santa Clara, CA, USA), and calcium concentration was determined by comparison with a standard curve. Data were analyzed using an unpaired Student’s *t*-test.

#### 4.11.1. TCA Metabolite Analyses

Tissue homogenization for metabolite retrieval. Tissue samples were collected on dry ice, weighed and then frozen at −80 °C until used. The tissues were then thawed on ice and resuspended in 600 µL of a 50:50 mixture of HPLC-grade methanol (Thermo Fisher Scientific, A456) and HPLC-grade water (Thermo Fisher Scientific, W64) containing 1 µg of norvaline (Thermo Fisher Scientific, AAA1590036) as internal standard. A blank sample containing only the methanol/water mixture and norvaline was included as a method control. The samples were transferred to Precellys tubes containing homogenization beads (P000945-LYSK0-A, Bertin Technologies, Rockville, MA, USA). The tissues were homogenized at 3 °C in a Precellys Evolution Tissue homogenizer (P002511-PEVT0-A.0, Bertin Technologies), followed by centrifugation at 6000 RPM for 20 s and then a pause for 120 s. This procedure was repeated three more times for complete homogenization. The homogenates were transferred to new 1.5 mL polypropylene tubes and mixed with 600 µL of HPLC-grade chloroform (C2974, Thermo Fisher Scientific). The samples were vortexed at 4 °C for 30 min and subsequently centrifuged at 17,000× *g* at 4 °C for 30 min. The top layer containing polar metabolites was transferred to a new 1.5 mL tube and dried in a SpeedVac (SRF110, Thermo Fisher Scientific) overnight at 4 °C.

#### 4.11.2. Sample Derivatization for GC-MS

After drying, samples were derivatized by adding 30 µL of a 2% *w*/*v* solution of methoxyamine hydrochloride (210490050, Thermo Fisher Scientific) in HPLC-grade pyridine (270407, Millipore Sigma, Burlington, MA, USA). The samples were placed in an incubated rotating shaker at 175 RPM at 45 °C for 60 min. Subsequently, 30 µL of N-tert-butyldimethylsilyl-N-methyltrifluoroacetamide with 1% tert-butyldimethylchlorosilane (M-108, Millipore Sigma) was added to the samples. The samples were then incubated in a rotator shaker at 175 RPM at 45 °C for 45 min. Afterward, the samples were transferred to glass inserts in glass vials for GC-MS analysis.

#### 4.11.3. GC-MS Analysis of Polar TCA Metabolites

Samples were analyzed using an Agilent 7890 GC equipped with a 30 m HP-5MSUI capillary column (19091S-433, Agilent, Santa Clara, CA, USA) connected to an Agilent 5977B MS in scan mode. One microliter of sample was injected. Electron ionization was set to 70 eV with helium carrier gas flow at 1 mL/min. The source was set to 230 °C, the front inlet to 270 °C, and the quadrupole at 150 °C. The following temperature protocol was utilized: (1) initial temperature—100 °C for 1 min, (2) increase to 255 °C at 3.5 °C/min, (3) increase to 320 °C at 15 °C/min, and (4) hold at 320 °C for 3 min. Samples were analyzed using Agilent Mass Hunter quantitative software. The moles of each metabolite in the sample were determined using an 8-point calibration curve with norvaline as an internal standard. A calibration blank containing only norvaline was also included as a method control. Final molar values were normalized to tissue mass.

### 4.12. Methyl Red Staining

A methyl red solution containing 22 mg Methyl red (CSA No. 493-52-7, Sigma, Burlington, MA, USA) in 10 mL 95% methanol was prepared and stored in a labeled, light-protected container. Developing mandibular incisors from WT, *Slc13a5*^R337*/R337*^, and *Odaph*^C41*/C41*^ D12 and D14 mice, at least 3 males and 3 females of each genotype, were carefully dissected from surrounding tissues and then cleaned with a 20% protease enzyme solution (12 min at 56 °C) to digest and remove soft tissue covering. The enamel surface was cleaned gently using Kimwipe and then immersed in the methyl red solution for 2 min to visualize pH-dependent coloration [46,47]. After excess solution was drained, the samples were air-dried and imaged using a Nikon SMZ 1000 dissecting microscope (Melville, New York, USA) and photographed with a Nikon DXM1200 digital camera (Melville, New York, USA).

The sample size of this experiment is listed below.
GenotypeTotalD12D14WTD12 *n* = 3; D14 *n* = 6*n* = 2M1F*n* = 2M4F*Slc13a5*^R337*/R337*^D12 *n* = 7; D14 *n* = 4*n* = 3M4F*n* = 2M2F*Odaph*^C41*/C41*^D12 *n* = 4; D14 *n* = 5*n* = 2M2F*n* = 3M2F

## 5. Conclusions

In conclusion, our findings demonstrate that loss of functional citrate transporter results in systemic citrate accumulation and exposes developing enamel and dentin to elevated local citrate concentrations. Citrate binds to hydroxyapatite; however, high concentrations of citrate can inhibit hydroxyapatite formation. Dentin is affected only mildly, but the growth of the early enamel ribbons on the surface of dentin appears to arrest almost immediately—before ameloblast pathology is evident. The simple failure to remove excessive citrate from the developing dentin and enamel matrices may be the fundamental cause of the observed effects on enamel and dentin formation.

## Data Availability

The mouse strain, MMRRC_067433-UCD (C57BL/6N-*Slc13a5^em1Jcch^*/Mmucd), is available at the Mutant Mouse Resources & Research Centers-UC Davis facility. The study data of wild-type, *Slc13a5*^+/R337*^, and *Slc13a5*^R337*/R337*^ are available in the FaceBase dataset 1-SBPG entitled “Effects of *Slc13a5* Gene Knock-out on Mouse Tooth Development”.

## Supplementary Materials

The following supporting information can be downloaded at: https://www.mdpi.com/article/10.3390/ijms27167129/s1.

## Abbreviations

The following abbreviations are used in this manuscript:

ANKH	Inorganic Pyrophosphate Transport Regulator
BCAA	Branched-chain amino acid
bSEM	Backscattered scanning electron microscopy
CIC	Mitochondrial citrate carrier
DEE25	Developmental and epileptic encephalopathy 25 with amelogenesis imperfecta
EDTA	Disodium ethylenediaminetetraacetic acid
EOE	Enamel organ epithelium
FIB-SEM	Focused ion beam–scanning electron microscopy
GC-MS	Gas Chromatography–Mass Spectrometry
LAMP1	Lysosomal-Associated Membrane Protein 1
MAT	Enamel/dentin matrix
MES	Pulpal mesenchyme
MIM	Mendelian Inheritance in Man
MMRRC	Mutant Mouse Resource and Research Centers
NaCT	Sodium-dependent citrate transporter
PCR	Polymerase chain reaction
PFA	Paraformaldehyde
RPM	Revolutions per minute
*Slc4a4*	Solute Carrier Family 4 Member 4
*Slc13a2*	Solute Carrier Family 13 Member 2
*Slc13a3*	Solute Carrier Family 13 Member 3
*Slc13a5*	Solute Carrier Family 13 Member 5
*Slc25a1*	Solute Carrier Family 25 Member 1
TCA	Tricarboxylic acid
WT	Wild-type
