# Supplementary material for: Citrate Transporter NaCT and Enamel Mineralization: The Slc13a5R337* Mouse Model
Source: Int J Mol Sci. 2026 Aug 9;27(16):7129. doi: 10.3390/ijms27167129 (PMC13513652; doi:10.3390/ijms27167129)
Supplement: Supplementary file 1 [file ijms-27-07129-s001.zip › ijms-4423222-supplementary.pdf]

List of Figures:

Figure S1: Photographs of 7 weeks old male and female *Slc13a5*$^{+/+}$, *Slc13a5*$^{+/R337*}$, and *Slc13a5*$^{R337*/R337*}$ mice

Figure S2: RT-PCR amplification of *Slc13a5*$^{+/+}$ (lanes 1) and *Slc13a5*$^{R337*/R337*}$ (lanes 2) mRNA transcripts in day 5 mouse first molars and in 7-week testis, seminal vesicles, and submandibular salivary gland.

Figure S3: *Slc13a5* and *Ank* gene expression in *Slc13a5*$^{+/+}$ *and Slc13a5* $^{R337*/R337*}$ mouse molars using RT-PCR.

Figure S4: *Ank* gene expression in *Slc13a5*$^{+/+}$ mouse tissues detected by RT-PCR.

Figure S5: RNAscope™ *in situ* hybridization characterization of WT *Slc13a5* mRNA expression in a mouse day 4 maxillary incisor (A-C) and a day 14 mandibular incisor (D-F).

Figure S6. bSEM of 7-week *Slc13a5*$^{R337*/R337*}$ mouse mandibular incisor cross-sections (sample 681).

Figure S7. bSEM of mandibular incisor cross-sections from a 7-week-old *Slc13a5*$^{R337*/R337*}$ mouse (sample 685).

Figure S8. bSEM of mandibular incisor cross-sections from a 7-week-old *Slc13a5*$^{R337*/R337*}$ mouse (sample 691).

Figure S9: Localization of *Slc13a5* and *Ank* mRNA in WT maxillary first molars at D0, D4, and D11. Images D–F were adapted, with permission, from Figure 12C of Hu *et al.* (2025), an open-access publication.

Figure S10. Expression of *Ank* in developing maxillary first molars of *Slc13a5*$^{+/+}$ and *Slc13a5*$^{R337*/R337*}$ mice.

Figure S11. Citrate concentrations in serum samples from D0^{+}, D3, D5, and D12 *Slc13a5*$^{+/+}$ (WT) and *Slc13a5*$^{R337*/R337*}$ (Mutant) mice.

Figure S12. Citrate contents of femur and tibia from 7- and 35-week-old *Slc13a5*$^{+/+}$ and *Slc13a5*$^{R337*/R337*}$ (Mutant) mice.

Figure S13. Micro-CT images of hemimandibles from postnatal D5 and D12 *Slc13a5*$^{+/+}$ and *Slc13a5*$^{R337*/R337*}$ mice.

Figure S14. Citrate and calcium contents in postnatal D5, and D12 enamel and dentin matrices in *Slc13a5*$^{+/+}$ (WT) and *Slc13a5*$^{R337*/R337*}$ (Mutant) first molars.

Figure S15. Intracellular concentration of branched chain amino acids (BCAAs) in *Slc13a5*$^{+/+}$ (WT) and *Slc13a5*$^{R337*/R337*}$ (Mutant) samples vary by genotype.

Figure S16. Intracellular concentration of branched-chain amino acids (BCAAs) in *Slc13a5*$^{+/+}$ (WT) and *Slc13a5*$^{R337*/R337*}$ (Mutant) vary by developmental day.

Figure S17. Alterations in non-TCA cycle metabolites between D5 and D12 molar enamel organ epithelium cells (EOE), pulpal mesenchyme cells (MES), and matrix (MAT) from *Slc13a5*$^{+/+}$ (WT) and *Slc13a5*$^{R337*/R337*}$ (Mutant) mice.

Figure S18. Alterations in non-TCA cycle metabolites between *Slc13a5*$^{+/+}$ (WT) and *Slc13a5*$^{R337*/R337*}$ (Mutant) enamel organ epithelium cells (EOE), pulpal mesenchyme cells (MES), and matrix (MAT) in D5 and D12 molar samples.

Figure S19. Comparison of pH variation on the labial surface of mandibular incisors from WT, *Slc13a5*$^{R337*/R337*}$, and *Odaph*$^{C41*/C41*}$ male and female mice using methyl red staining.

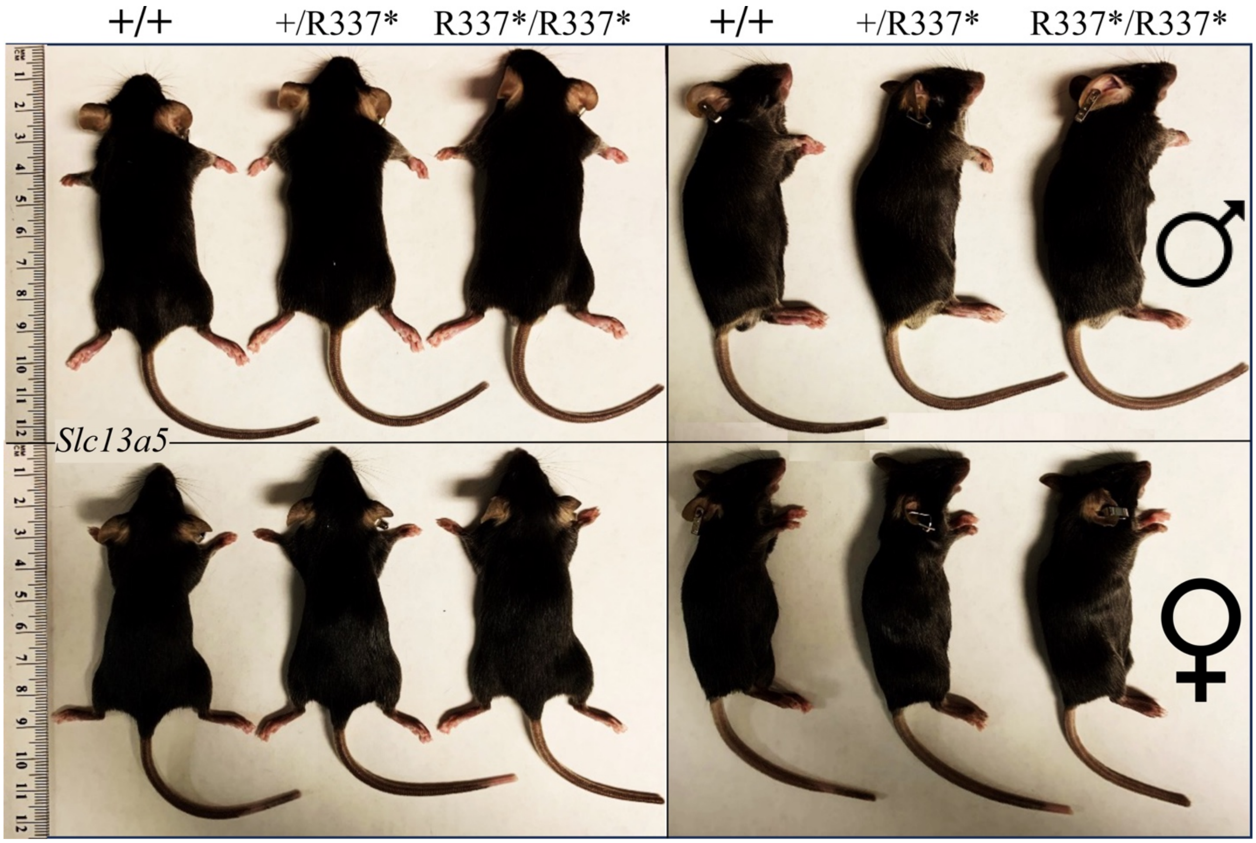

Figure S1. Photographs of 7 weeks old male and female *Slc13a5*$^{+/+}$, *Slc13a5*$^{+/R337*}$, and *Slc13a5*$^{R337*/R337*}$ mice. Representative male mice (top) and female mice (bottom) from a pool of 6 WT (3 male and 3 female), 6 *Slc13a5*$^{+/R337*}$ (3 male and 3 female), and 10 *Slc13a5*$^{R337*/R337*}$ (5 male and 5 female) mice. There were no apparent differences in body size or proportions. The average body weights (in grams) of the male mice were 21.95 (*Slc13a5*$^{+/+}$), 21.41 (*Slc13a5*$^{+/R337*}$), 21.87 (*Slc13a5*$^{R337*/R337*}$) and female mice were 17.52 (*Slc13a5*$^{+/+}$), 17.73 (*Slc13a5*$^{+/R337*}$), 15.96 (*Slc13a5*$^{R337*/R337*}$).

A

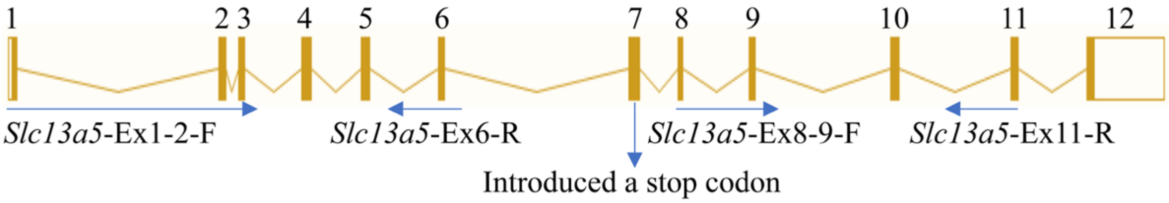

B

Gene	Primer F	Primer R	Region covered	Amplicon size (bp)
Slc13a5	CTGATACCTGACAAGTTTGCCA	CTCATGTACAAGCACTGGAGC	Ex1-6	755
Slc13a5	CCAGACTGAGGAAGAAAGGAAA	TCCAAGTTAAACATAGCCCGA	Ex8-11	522
Gapdh	AGGCCGGTGCTGAGTATGTC	TGCCTGCTTCACCACCTTCT		530

C

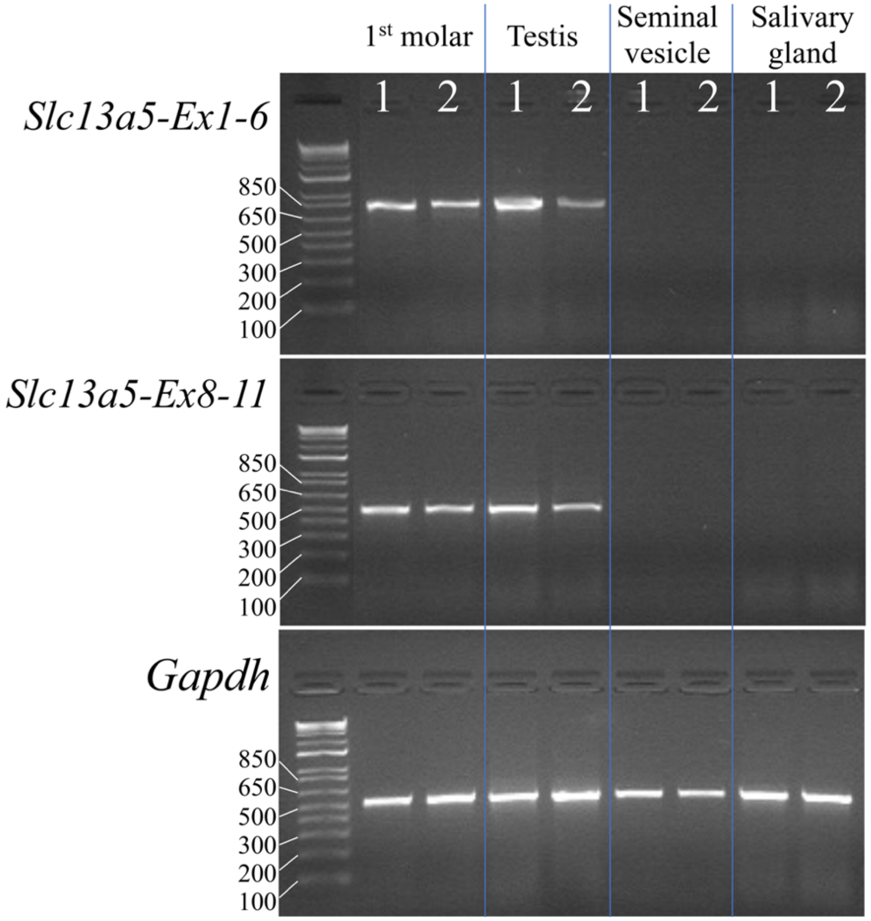

Figure S2. RT-PCR amplification of *Slc13a5*$^{+/+}$ (lanes 1) and *Slc13a5*$^{R337*/R337*}$ (lanes 2) mRNA transcripts in day 5 mouse first molars and in 7-week testis, seminal vesicles, and submandibular salivary gland. (A) *Slc13a5* gene diagram displaying PCR primer locations (blue arrows) for *Slc13a5*$^{+/+}$ and *Slc13a5*$^{R337*/R337*}$ amplifications. (B) Primer pair sequences and amplicon sizes in this experiment. (C) Agarose gels showing the *Slc13a5* gene is expressed in the EOE of D5 molars and 7-week-old testis, but is not expressed in 7-week-old seminal vesicle or salivary gland. The sample size was one. Experiment was conducted twice using different samples.

A

Gene	Primer F	Primer R	Region covered	Amplicon size (bp)
Slc13a5	CTGATACCTGACAAGTTTGCCA	CTCATGTACAAGCACTGGAGC	Ex1-6	755
Slc13a5	CCAGACTGAGGAAGAAAGGAAA	TCCAAGTTAAACATAGCCCGA	Ex8-11	522
Ank	CCACACCCTGATAGCCTACA	CAAAGGCAAAGTCCACTCCA	Ex2-9	791
Gapdh	AGGCCGGTGCTGAGTATGTC	TGCCTGCTTCACCACCTTCT		530

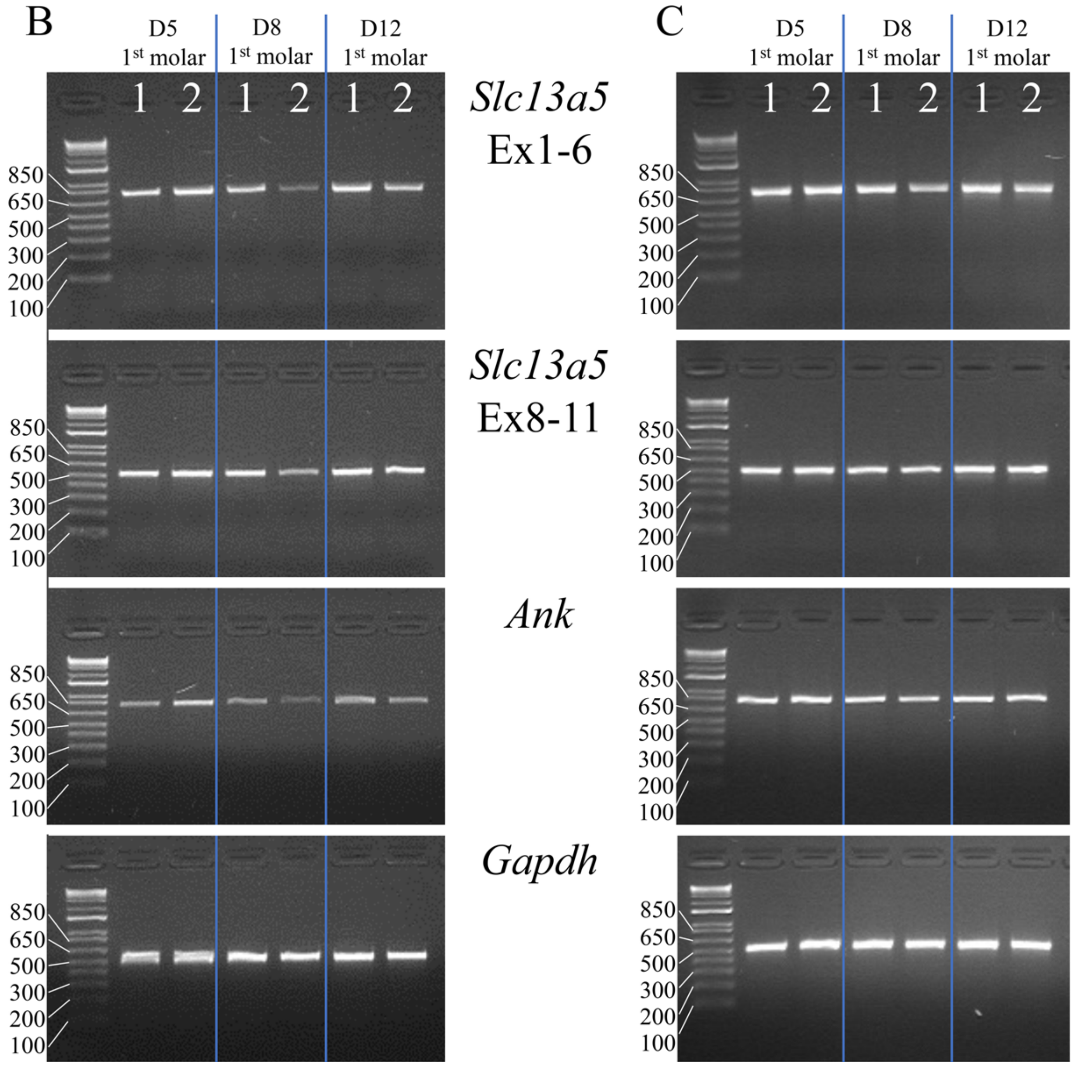

Figure S3. *Slc13a5* and *Ank* gene expression in *Slc13a5*$^{+/+}$ *and Slc13a5* $^{R337*/R337*}$ mouse molars using RT-PCR. (A) Expression was examined in D5 first molars and in salivary glands, testes, and seminal vesicles from 7-week-old mice. (A) *Slc13a5* primer sequences, targeted regions, and amplicon sizes. (B, C) *Slc13a5* expression was detected in D5, D8, and D12 molars. *Ank* expression was also detected in D5, D8, and D12 molars. The dissected molars used in this experiment were enclosed in follicular tissues. cDNA synthesis was performed using oligo(dT) primers (B) and random hexamers (C). Lane 1: WT; Lane 2: *Slc13a5*$^{R337*/R337*}$. DNA ladder: 1 kb Plus DNA Ladder, Invitrogen. At each time point, sample size was one. Experiment was conducted twice using different samples.

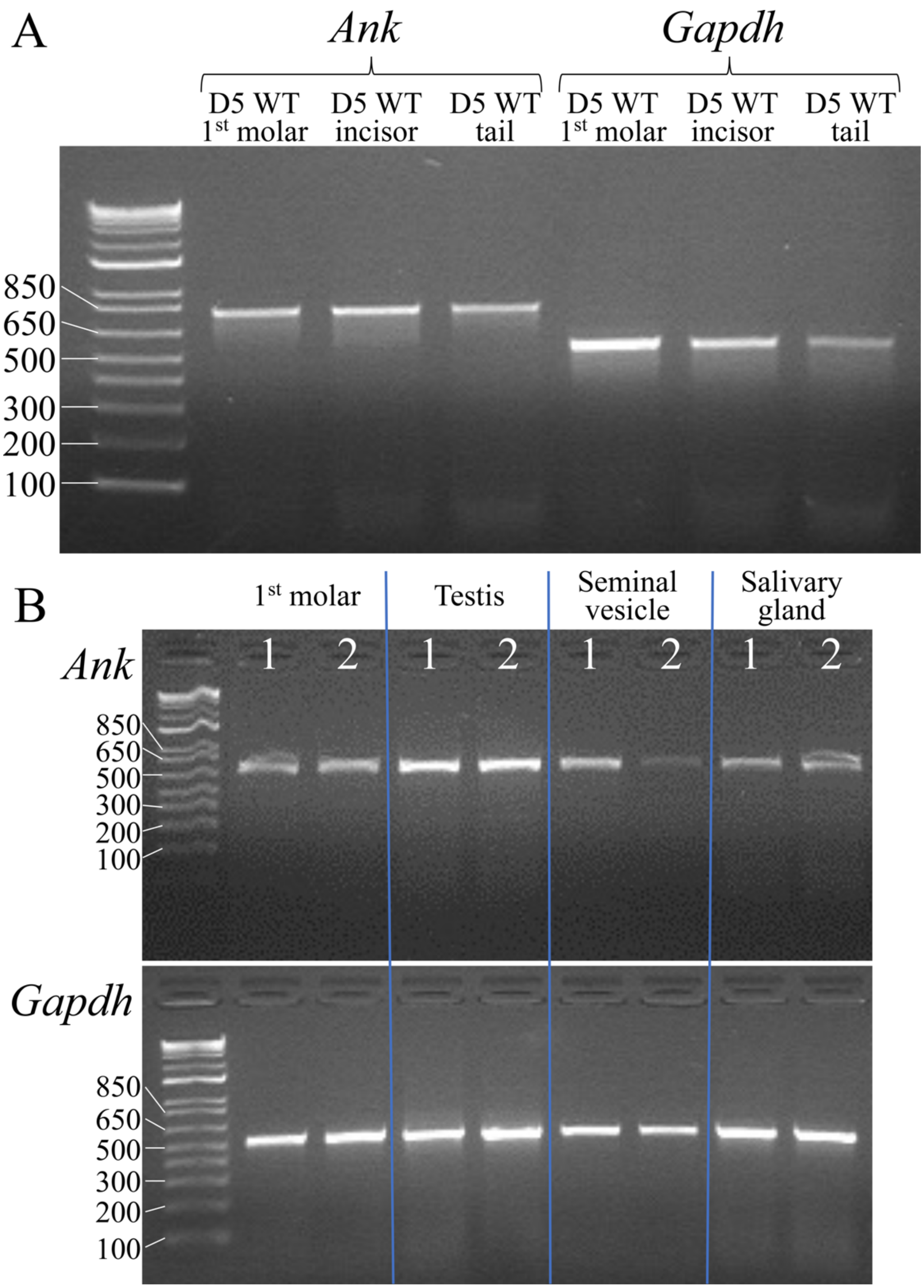

Figure S4. *Ank* gene expression in *Slc13a5*$^{+/+}$ mouse tissues detected by RT-PCR. (A) Amplicon of *Ank*, 791 bp, and *Gapdh*, 530 bp were detected in first molar, incisor, and tail from D5 *Slc13a5*$^{+/+}$ mice. (B) *Ank* is expressed in D5 first molars, and 7-week testis, seminal vesicles, and submandibular salivary glands, from *Slc13a5*$^{+/+}$ mice. By comparing *Ank* the *in situ* hybridization results (Figure 5), the positive amplifications are derived from the mRNA in osteoblast, preodontoblasts and preameloblasts in these developing tooth organs. *cDNA was synthesized using random hexamer and the amount of input for RT-PCR was equalized for each reaction. Lane 1: wild-type; Lane 2: *Slc13a5*$^{R337*/R337*}$. DNA ladder, 1kb plus from Invitrogen. Sample number from each time point was one. This experiment was not duplicated.

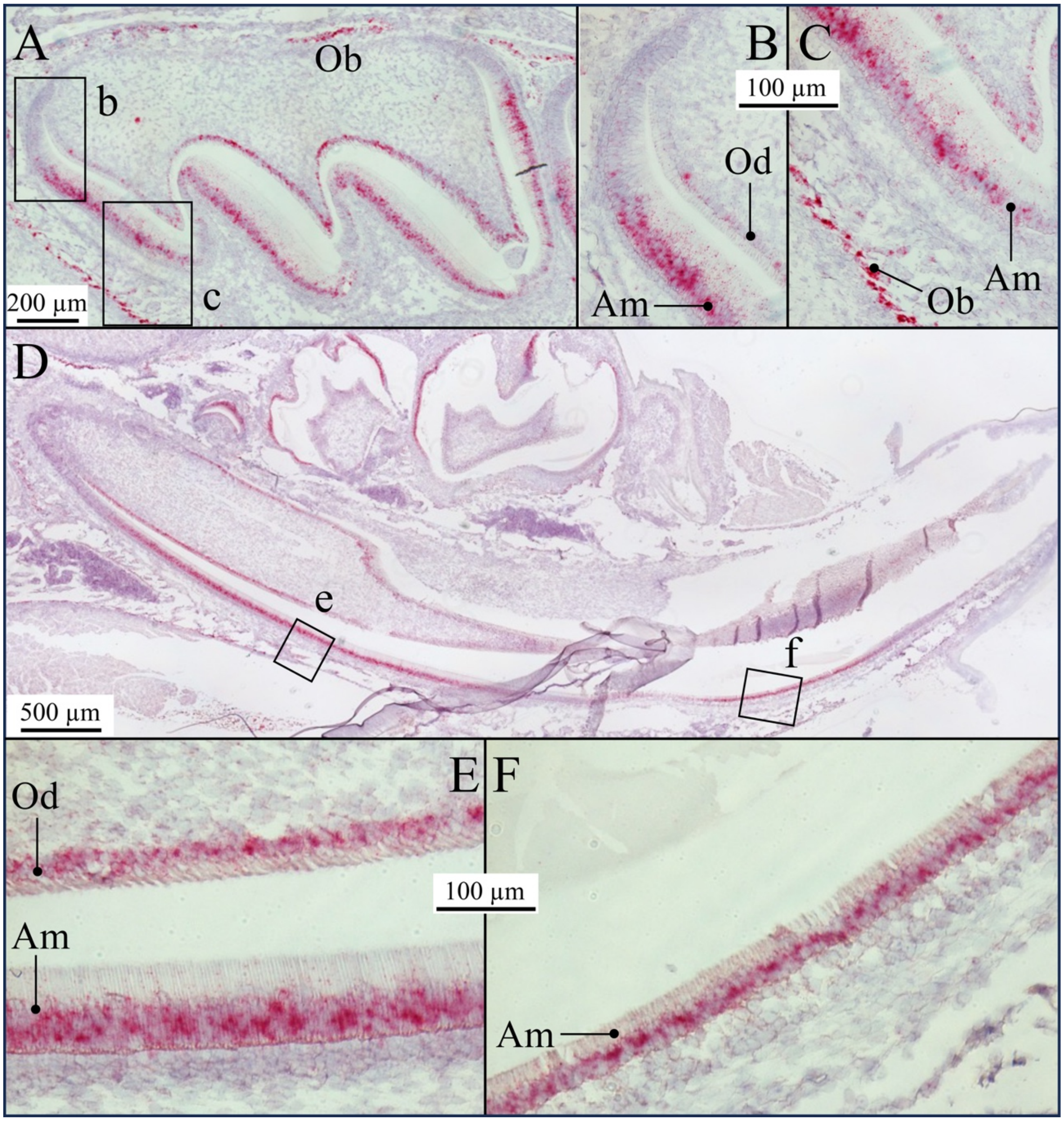

Figure S5. RNAscope[TM] *in situ* hybridization characterization of WT *Slc13a5* mRNA expression in a mouse day 4 maxillary incisor (A-C) and a day 14 mandibular incisor (D-F). (A) The day 4 developing maxillary molar shows positive *Slc13a5* mRNA expression in secretory ameloblasts (Am), odontoblasts (Od), as well as in osteoblasts in surrounding developing bone. (B) Presecretory developing ameloblasts in the cervical loops are trace to negative. (C) Osteoblasts (Ob) and secretory ameloblasts generally show stronger *Slc13a5* mRNA expression than odontoblasts, (D) The day 14 mandibular incisor shows early development on the left and progressively later development to the right. Odontoblasts are positive for *Slc13a5* mRNA expression before the overlying ameloblasts, both preceding the onsets of dentin and enamel biomineralization, but expression by odontoblasts diminishes beneath secretory ameloblasts in the latter half of the secretory stage. (E) *Slc13a5* mRNA expression is strong in secretory stage ameloblasts and underlying odontoblasts. (F) *Slc13a5* mRNA expression is strong in maturation stage ameloblasts but is no longer expressed by odontoblasts. Key: Am, secretory-stage ameloblasts; Od, odontoblasts; Ob, osteoblasts; mAm, maturation-stage ameloblasts. This was a pilot study; sample size was one from each time point.

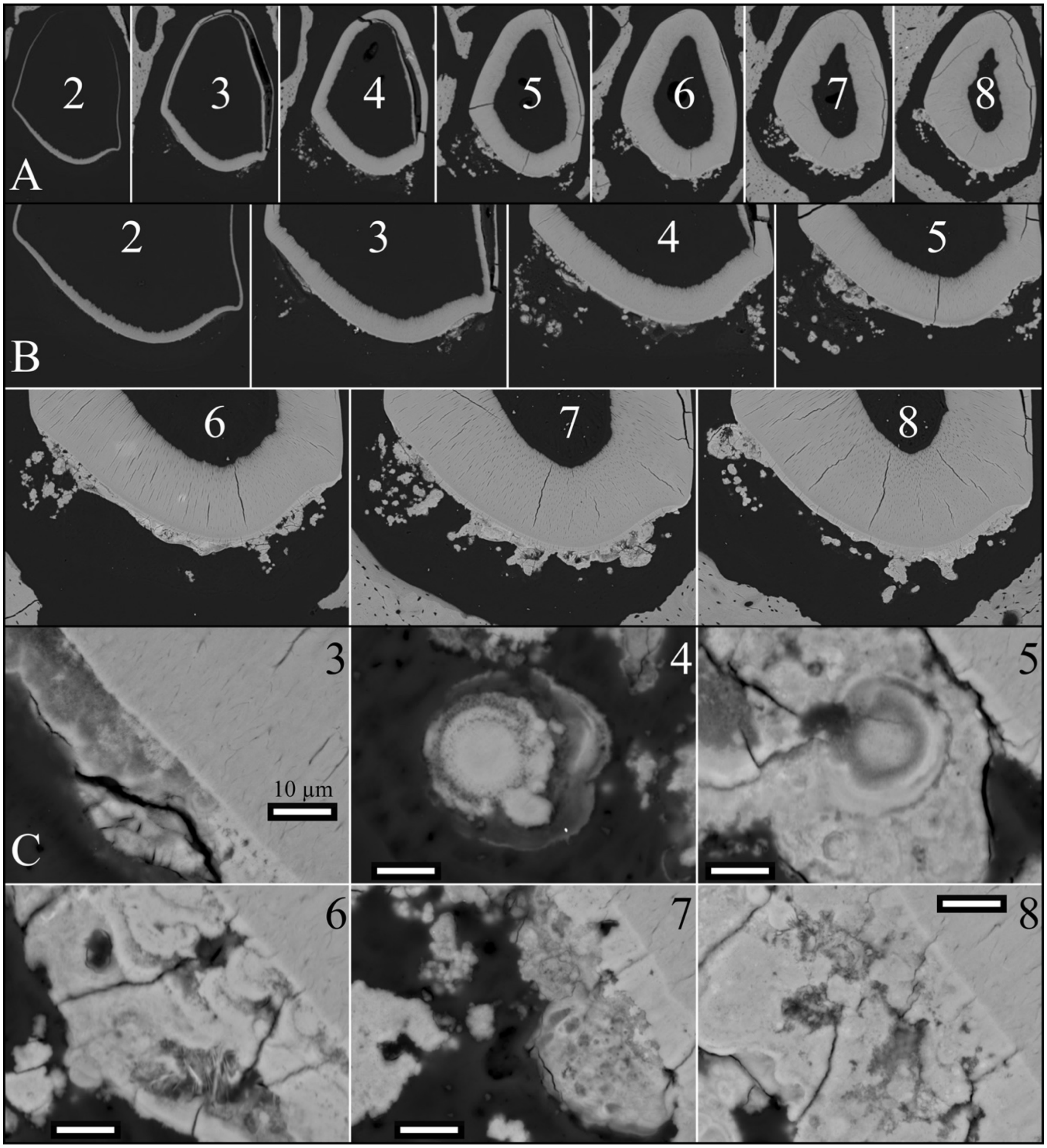

Figure S6. bSEM of 7-week *Slc13a5*$^{R337*/R337*}$ mouse mandibular incisor cross-sections (sample 681). (A) Cross-sections taken at 1 mm increments, with increment 3 corresponding to the completion of secretory stage when the enamel has normally reached full thickness (~120 µm at its maximum that diminishes laterally), but is still hypomineralized. Dentin continues to expand, which diminishes the size of the pulp space. (B) Higher magnification images of buccal region where enamel forms. In the *Slc13a5*$^{R337*/R337*}$ mandibular incisor a very thin "enamel" mineral forms (~10 µm thick) on the surface of dentin followed by the deposition of an irregular mineral crust on the buccal surface and ectopic mineral nodules within the enamel organ epithelia. (C) Higher-magnification images detailing pathological mineralization nodules deposited during the maturation stage. Concentric rings were sometimes observed in the mineral nodules. Six samples were analyzed in this experiment.

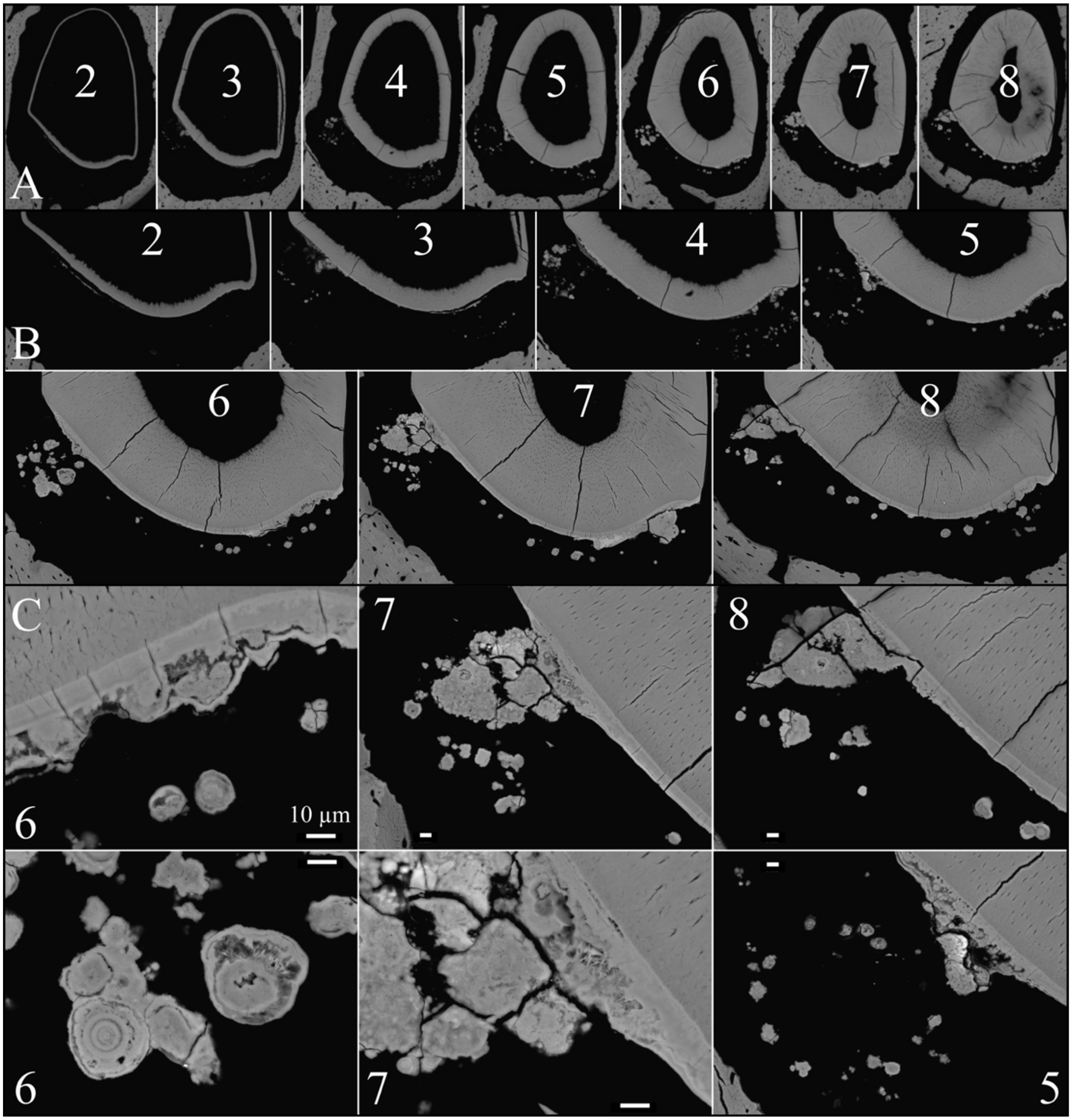

Figure S7. bSEM of mandibular incisor cross-sections from a 7-week-old *Slc13a5*$^{R337*/R337*}$ mouse (sample 685). (A) Cross-sections taken at 1 mm increments, with increment 3 corresponding to the completion of secretory stage when the enamel has normally reached full thickness (~120 µm at its maximum that diminishes laterally), but is still hypomineralized. Dentin continues to expand, progressively diminishing the pulp space. (B) Higher magnification images of buccal region where enamel forms. In the *Slc13a5*$^{R337*/R337*}$ mandibular incisor a very thin "enamel" mineral forms (~10 µm thick) on the surface of dentin followed by the deposition of an irregular mineral crust on the buccal surface and ectopic mineral nodules within the enamel organ epithelia. (C) Higher-magnification images detailing pathological mineralization nodules deposited during the maturation stage. Note that the overall pattern of the pathological mineralization in *Slc13a5*$^{R337*/R337*}$ incisors varies in each incisor. Six samples were analyzed in this experiment.

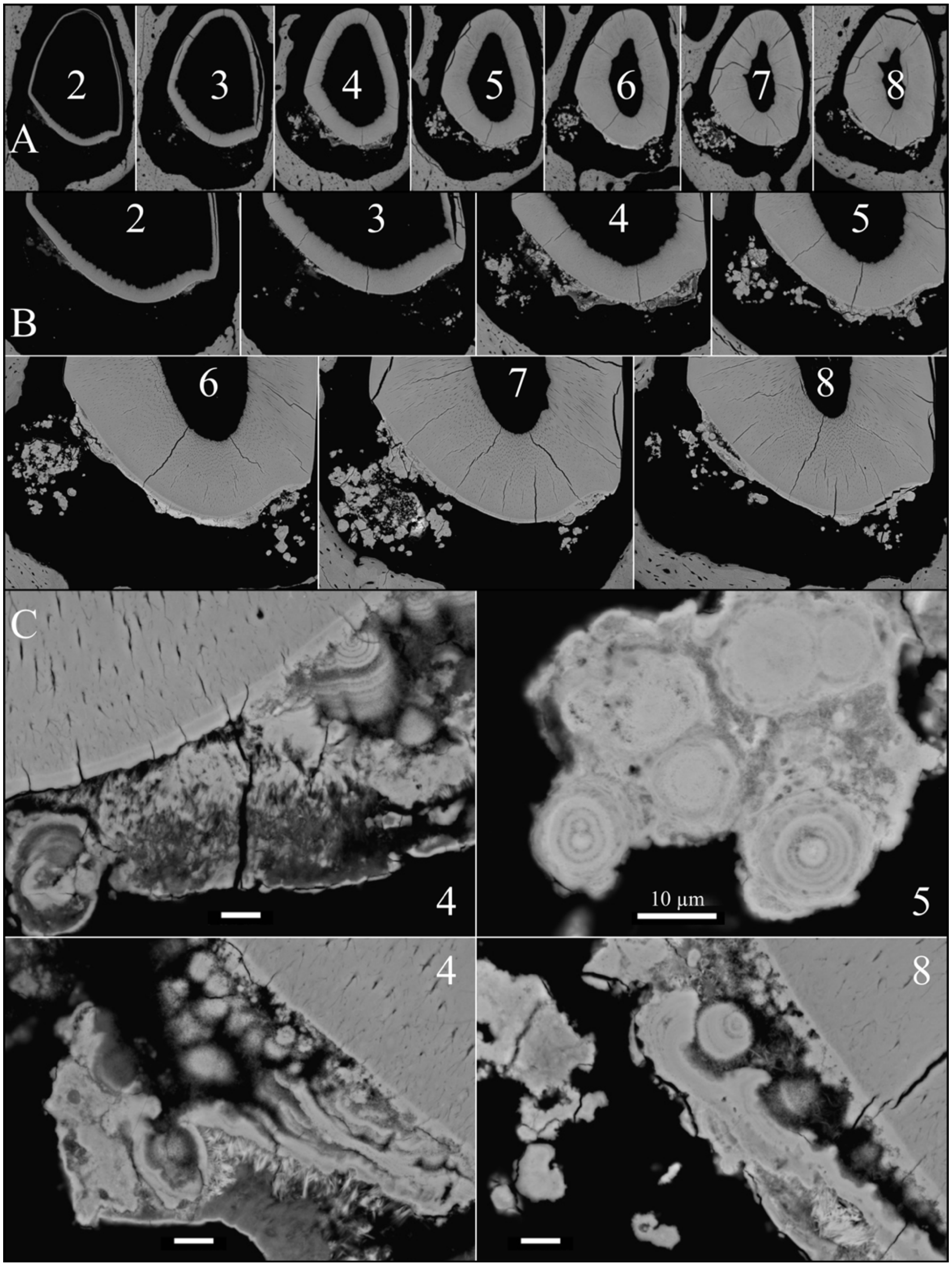

Figure S8. bSEM of mandibular incisor cross-sections from a 7-week-old *Slc13a5*$^{R337*/R337*}$ mouse (sample 691). (A) Incisor cross-sections taken at 1 mm increments. (B) Higher magnification images of buccal region where "enamel" forms. (C) Higher-magnification images detailing pathological mineralization nodules deposited during the maturation stage. Note that the overall pattern of the pathological mineralization in *Slc13a5*$^{R337*/R337*}$ incisors varies in each incisor. Here the thin mineral layer in contact with the dentin surface is <10 µm in thickness. There is no normal enamel formation and pathological mineralization nodules form in random patterns as in samples 681 and 685. Six samples were analyzed in this experiment.

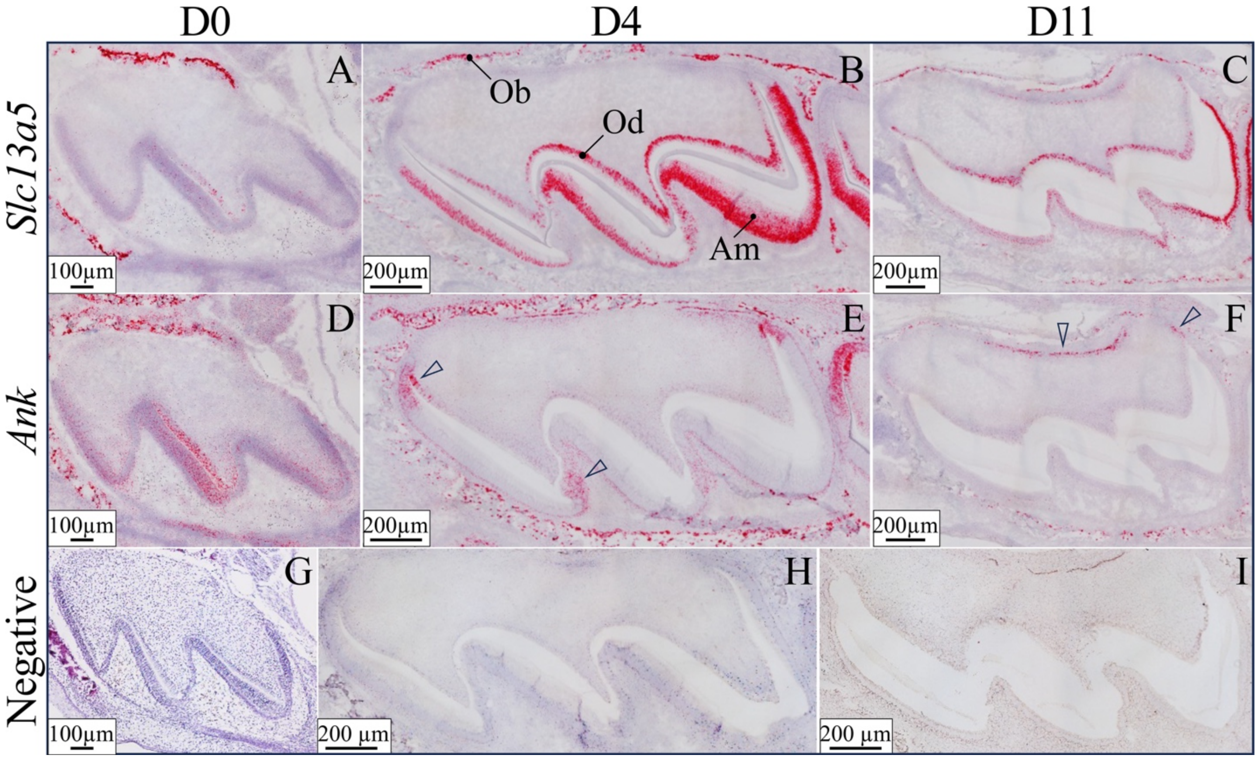

Figure S9. Localization of *Slc13a5* and *Ank* mRNA in WT maxillary first molars at D0, D4, and D11. Sections (5 µm) of developing mouse maxillary molars at D0 (newborn; A, D, G), postnatal D4 (secretory stage; B, E, H), and D11 (maturation stage; C, F, I) were stained red by in situ hybridization using oligonucleotide probes for *Slc13a5* (A-C), *Ank* (D-F), or Negative controls without probes (G-I). *Slc13a5* expression increased in ameloblasts (Am) and odontoblasts as they differentiated (A) and expanded the enamel and dentin layers (B) and persisted at lower levels during the maturation stage (C). *Ank* mRNA was restricted to developing ameloblasts and odontoblasts (D), the cervical loops and enamel free zones (E arrowheads)—but not by odontoblasts (E, F) secretory (E), or maturation (F) ameloblasts making dentin and enamel, but was osteoblasts (D, E) and diaphragm and cervical loop (F, arrowheads), which explains the positive RT-PCR results in Figures S3 and S4. Key: Am, secretory-stage ameloblasts; Od, odontoblasts; Ob, osteoblasts. Images D–F were adapted, with permission, from Figure 12C of Hu *et al.* (2025), an open-access publication. Two samples from each genotype and each time point were used.

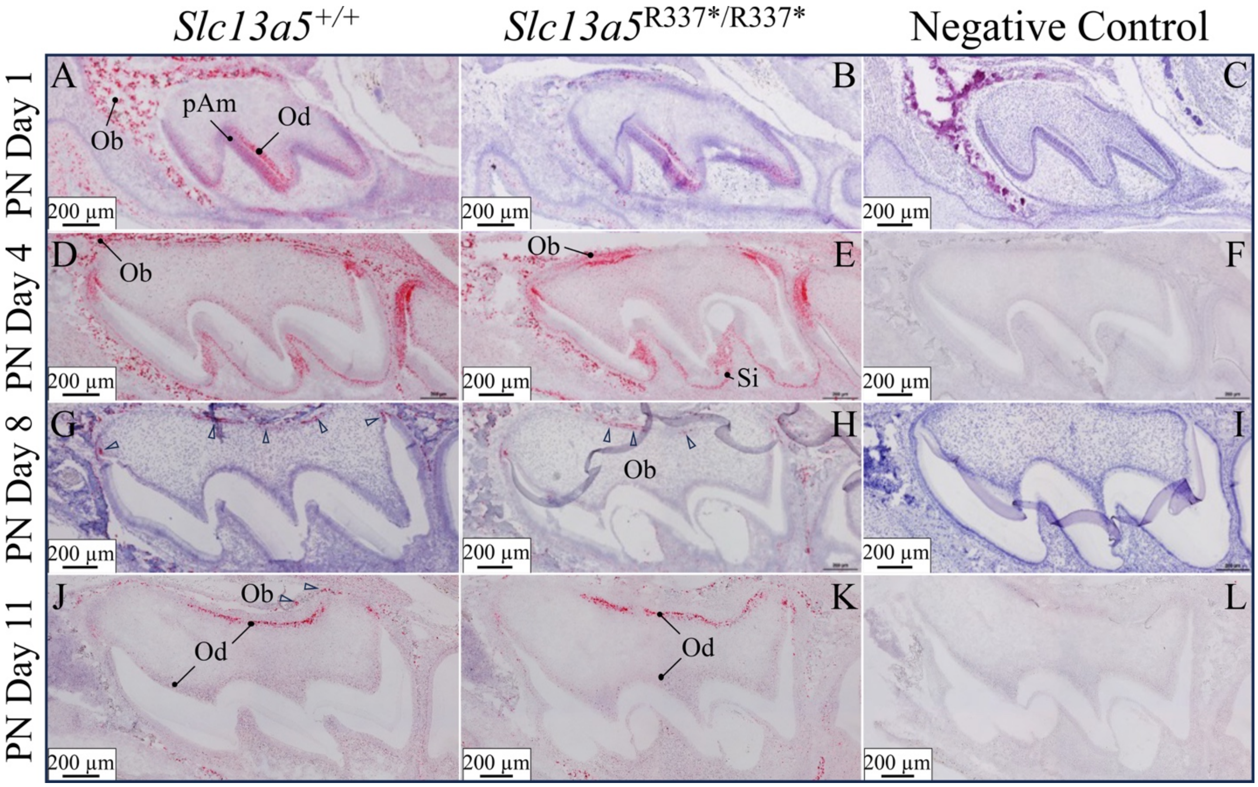

Figure S10. Expression of *Ank* in developing maxillary first molars of *Slc13a5*$^{+/+}$ and *Slc13a5*$^{R337*/R337*}$ mice. (A-C) WT and *Slc13a5*$^{R337*/R337*}$ Day 0 molars show *Ank* expression in early odontoblasts (Od), pre-ameloblasts (pAm), and osteoblasts (Ob) in alveolar bone. In WT and *Slc13a5*$^{R337*/R337*}$ day 4 molars (D-E) *Ank* is expressed by osteoblasts (and associated diaphragm) during early root bifurcation, in pre-odontoblast and pre-ameloblasts, and the stratum intermedium (Si), but is not expressed by odontoblasts and ameloblasts during dentin and enamel formation. In WT and *Slc13a5*$^{R337*/R337*}$ day 8 molars (G-H) *Ank* is expression is limited to pre-odontoblasts and pre-ameloblast near the cervical loop (arrowheads). In WT and *Slc13a5*$^{R337*/R337*}$ day 11 molars (J-K) *Ank* expression is limited to osteoblasts at the furcation area and pre-odontoblast on the pulpal side of the developing diaphragm (arrowheads). No background signal was observed in negative experimental controls (C, F, I, L) Key: Ob, osteoblast; Od, odontoblast; pAm, pre-ameloblast; Si, stratum intermedium. Two samples from each genotype and each time point were used.

A

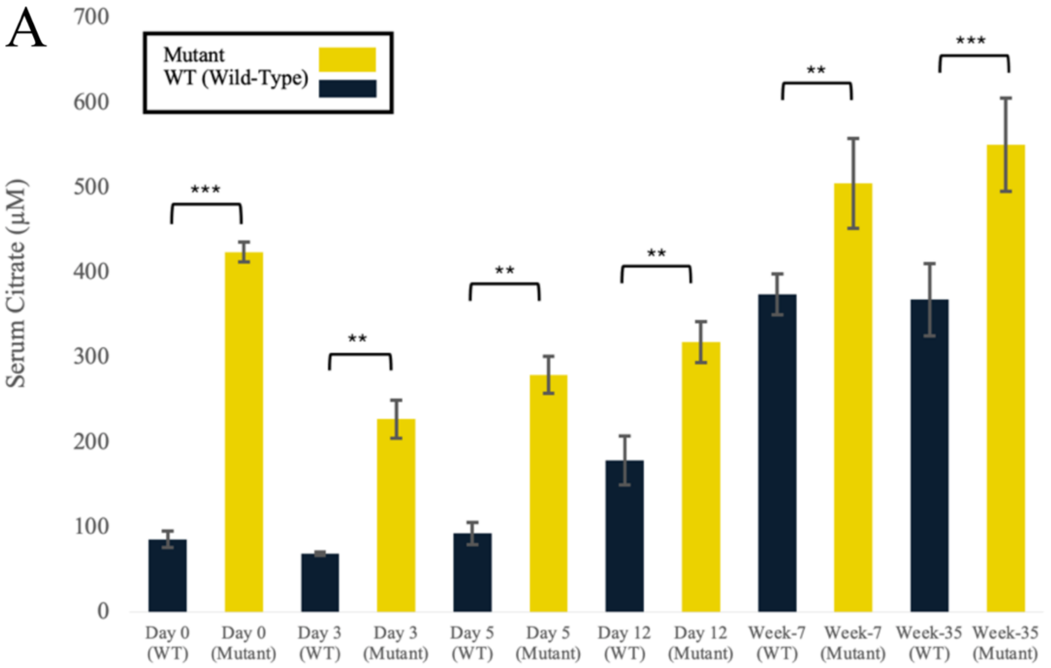

B

Sample number	Sample read	Sample blank read	Calibrated Sample read	Citrate conc.(μM) based on read1	Citrate conc.(μM) based on read2	Citrate conc.(μM)	Mean ± SD (μM)
WT D0 2	0.332	0.22	0.112	74.06	72.35	73.20	
WT D0 3	0.343	0.223	0.12	79.35	77.51	78.43	85.30±9.79
WT D0 4	0.4	0.259	0.141	93.24	91.08	92.16	
Slc-Mutant D0 4	0.844	0.226	0.618	408.67	399.19	403.93	
Slc-Mutant D0 6	0.865	0.222	0.643	425.20	415.34	420.27	423.54±11.78
Slc-Mutant D0 7	0.838	0.185	0.653	431.82	421.80	426.81	
WT D3 4	0.348	0.238	0.11	72.74	71.05	71.90	
WT D3 6	0.32	0.214	0.106	70.10	68.47	69.28	68.63±2.00
WT D3 7	0.309	0.205	0.104	68.77	67.18	67.98	
Slc-Mutant D3 5	0.663	0.276	0.387	255.92	249.98	252.95	
Slc-Mutant D3 8	0.504	0.182	0.322	212.93	207.99	210.46	226.80±22.25
Slc-Mutant D3 9	0.614	0.242	0.372	246.00	240.29	243.14	
WT D5 2	0.327	0.138	0.189	70.30617	73.4013123	71.85	
WT D5 3	0.461	0.204	0.257	95.601512	99.81025	97.71	92.19±12.98
WT D5 5	0.448	0.22	0.228	84.813793	88.5476148	86.68	
Slc-Mutant D5 1	0.812	0.173	0.639	237.70181	248.166342	242.93	
Slc-Mutant D5 2	0.912	0.197	0.715	265.97308	277.682213	271.83	279.05±22.07
Slc-Mutant D5 4	0.959	0.206	0.753	280.10871	292.440149	286.27	
WT D12 2	0.478	0.215	0.263	146.75018	153.210676	149.98	
WT D12 3	0.465	0.194	0.271	151.21406	157.871076	154.54	178.21±28.73
WT D12 4	0.62	0.266	0.354	197.52686	206.222735	201.87	
Slc-Mutant D12 1	0.754	0.208	0.546	304.66007	318.072353	311.37	
Slc-Mutant D12 2	0.708	0.193	0.515	287.36252	300.0133	293.69	317.64±24.22
Slc-Mutant D12 3	0.831	0.232	0.599	334.2333	348.947508	341.59	

Figure S11. Citrate concentrations in serum samples from D0^{+}, D3, D5, and D12 *Slc13a5*$^{+/+}$ (WT) and *Slc13a5*$^{R337*/R337*}$ (Mutant) mice. (A) Serum citrate concentrations varied with time but were always significantly higher in *Slc13a5*$^{R337*/R337*}$ mice than in WT mice (p < 0.05). In both genotypes, Day 0+ pups had higher citrate concentrations than Day 3 pups of the same genotype. In *Slc13a5*$^{R337*/R337*}$ mice, Day 0+ pups had higher citrate concentrations than Day 5 or Day 12 pups of the same genotype. (B) Table of serum citrate measurements. At each time point, sample size was three from each genotype.

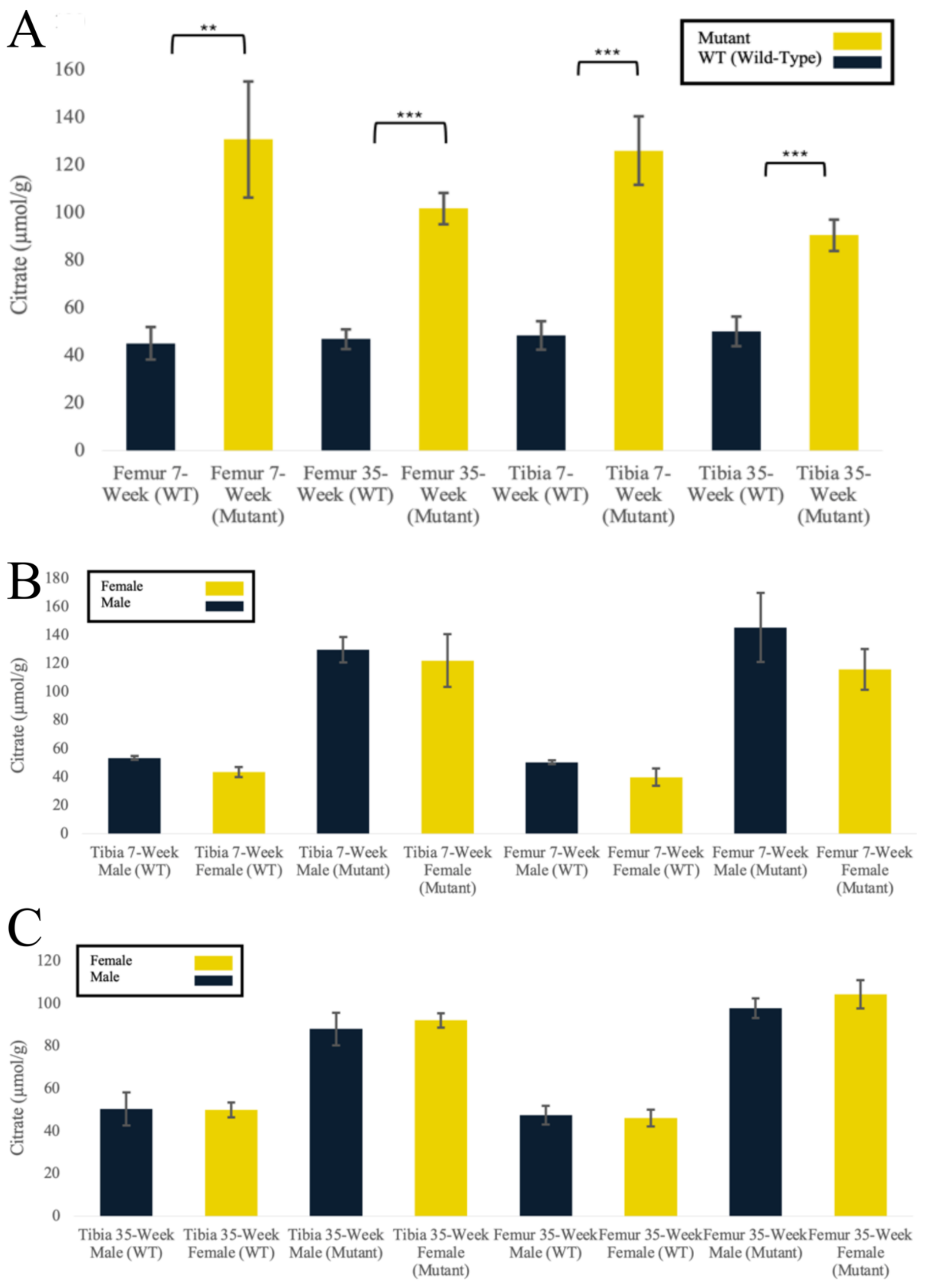

Figure S12. Citrate contents of femur and tibia from 7- and 35-week-old *Slc13a5*$^{+/+}$ (WT) and *Slc13a5*$^{R337*/R337*}$ (Mutant) mice. (A) Femurs and tibias from *Slc13a5*$^{R337*/R337*}$ mice contained significantly higher citrate levels than those from WT mice ($p < 0.05$). (B, C) No statistically significant differences in citrate content were observed between male and female samples of the same genotype at either 7 or 35 weeks of age. Sample sizes were as follows: for 35-week-old mice, WT: 7 males and 7 females; mutant: 4 males and 4 females. For 7-week-old mice, WT: 3 males and 3 females; mutant: 5 males and 5 females.

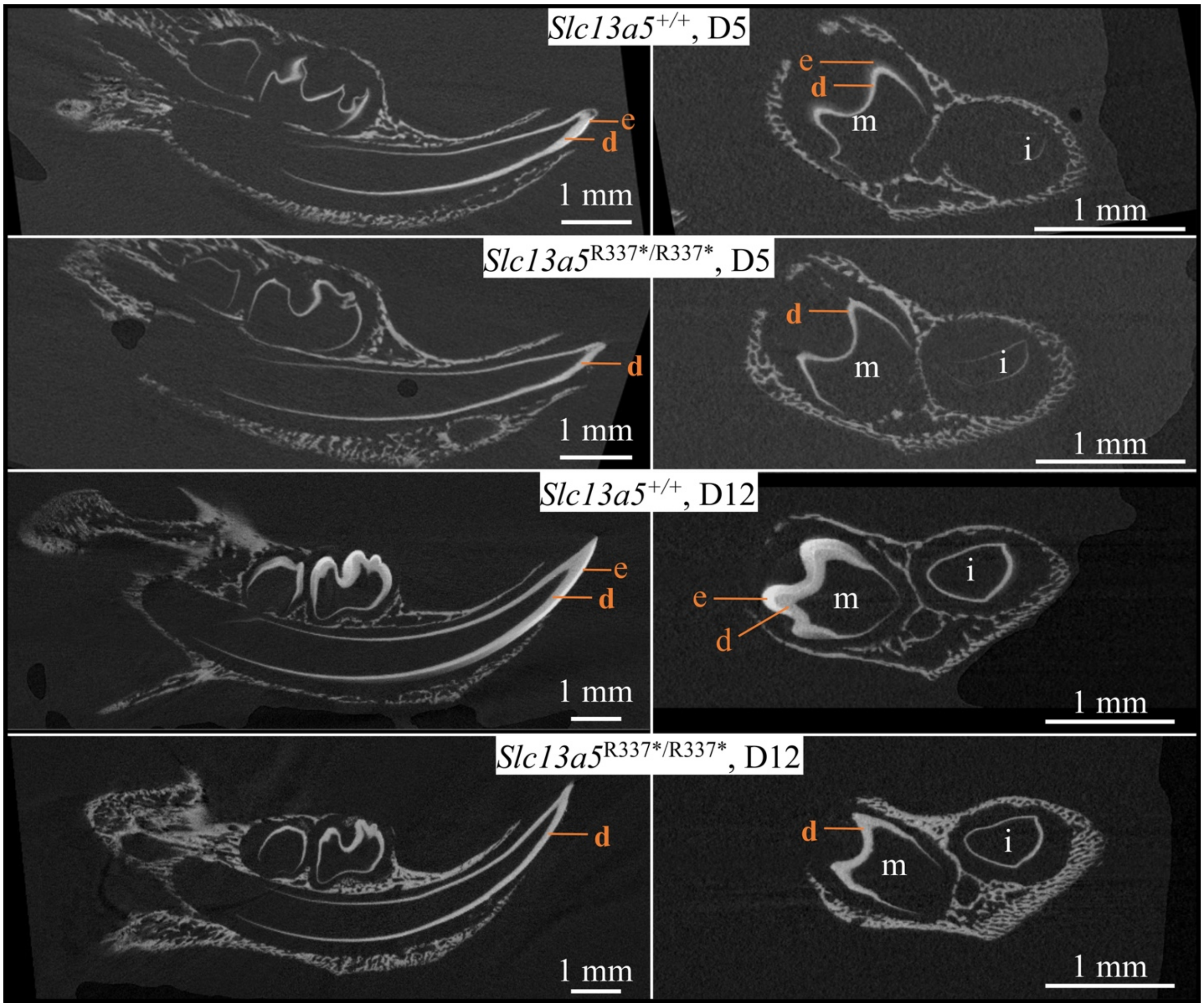

Figure S13. Micro-CT images of hemimandibles from postnatal D5 and D12 *Slc13a5*$^{+/+}$ and *Slc13a5*$^{R337*/R337*}$ mice. Sagittal sections of the hemimandibles are shown on the left, and cross-sections are shown on the right. The size and shape of the developing molars and incisors were comparable between groups. However, the mineralized matrix, particularly enamel, was consistently absent in the D5 and D12 *Slc13a5*$^{R337*/R337*}$ samples. Key: e, enamel; d, dentin; e+d, enamel and dentin; m, molar; i, incisor. At each time point, four hemimandibles from two mice of each genotype were imaged.

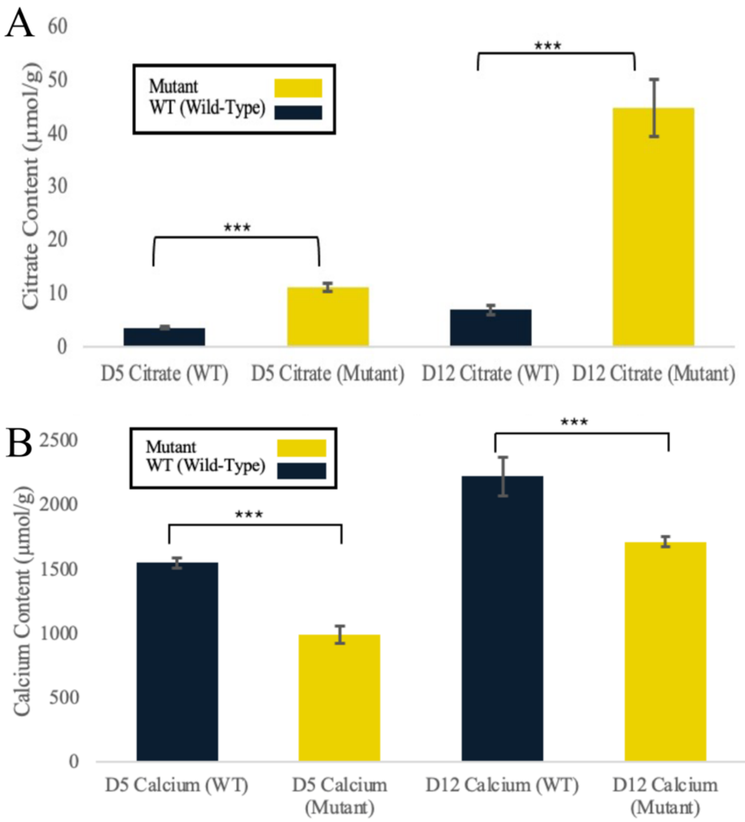

Figure S14. Citrate and calcium contents in postnatal D5, and D12 enamel and dentin matrices in *Slc13a5*$^{+/+}$ (WT) and *Slc13a5*$^{R337*/R337*}$ (Mutant) first molars. Bound citrate (A) and calcium (B) contents were determined and compared. At D5, and D12, bound citrate from the developing enamel and dentin matrix of *Slc13a5*$^{R337*/R337*}$ mice contained significantly higher citrate levels ($p < 0.05$) and lower calcium levels ($p < 0.05$) compared with WT mice. At each time point, three mice from each genotype were used.

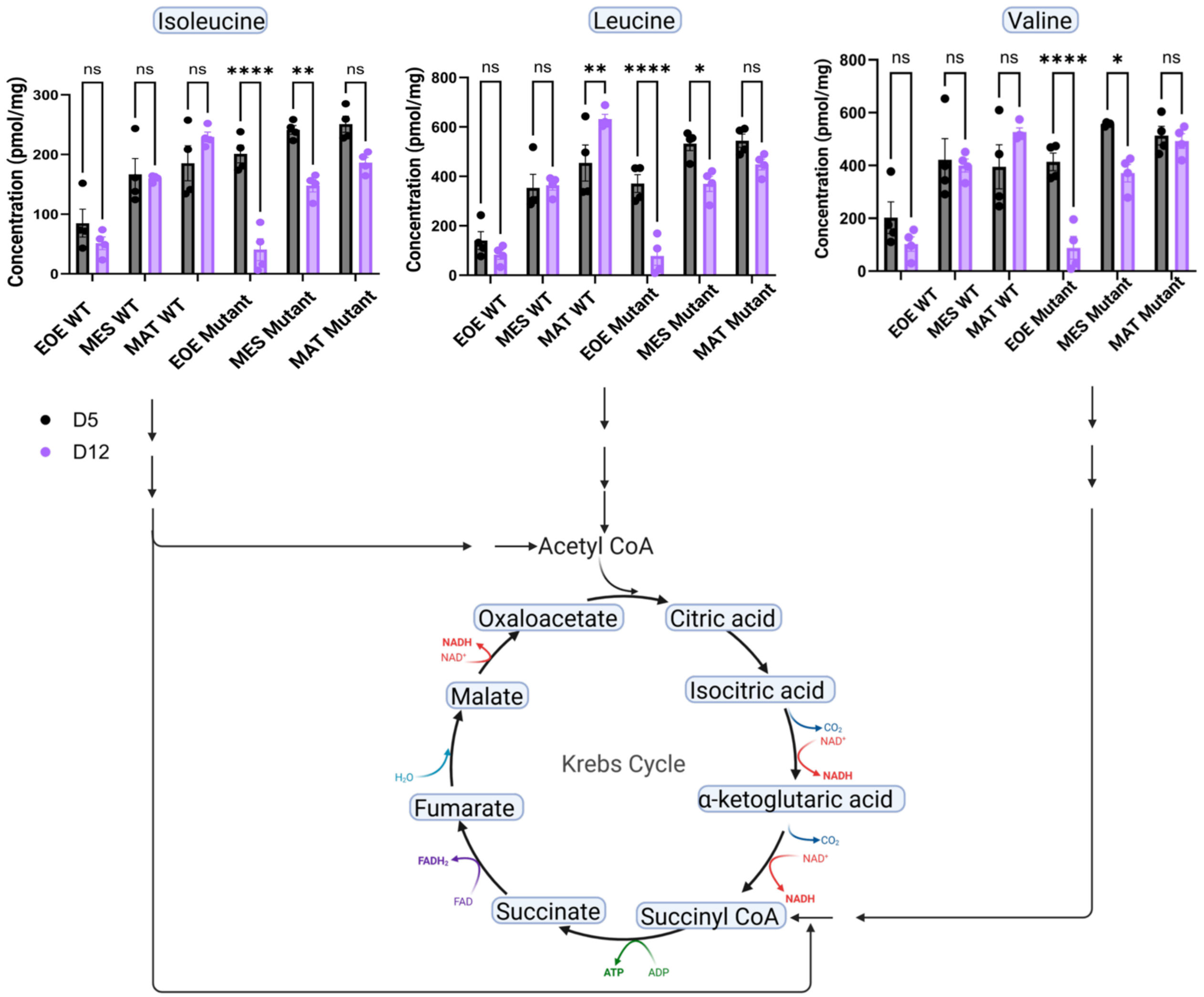

Figure S15. Intracellular concentration of branched chain amino acids (BCAAs) in *Slc13a5*$^{+/+}$ (WT) and *Slc13a5*$^{R337*/R337*}$ (Mutant) samples vary by genotype. Developing enamel organ epithelium (EOE) and pulpal mesenchyme (MSE) from *Slc13a5*$^{R337*/R337*}$ mice showed increased intracellular accumulation of BCAAs at day 5. At day 12, matrix samples showed no significant differences in BCAA levels between *Slc13a5*$^{R337*/R337*}$ and WT mice. Statistical significance was determined using ordinary two-way ANOVA, with $p < 0.05$ considered significant. Key: not significant (ns), increasing significance (* p < 0.05, ** p < 0.01, *** p < 0.001, **** p < 0.0001). At each time point, there were three samples from each genotype; each sample contained tissues from 12 molars of 3 mice.

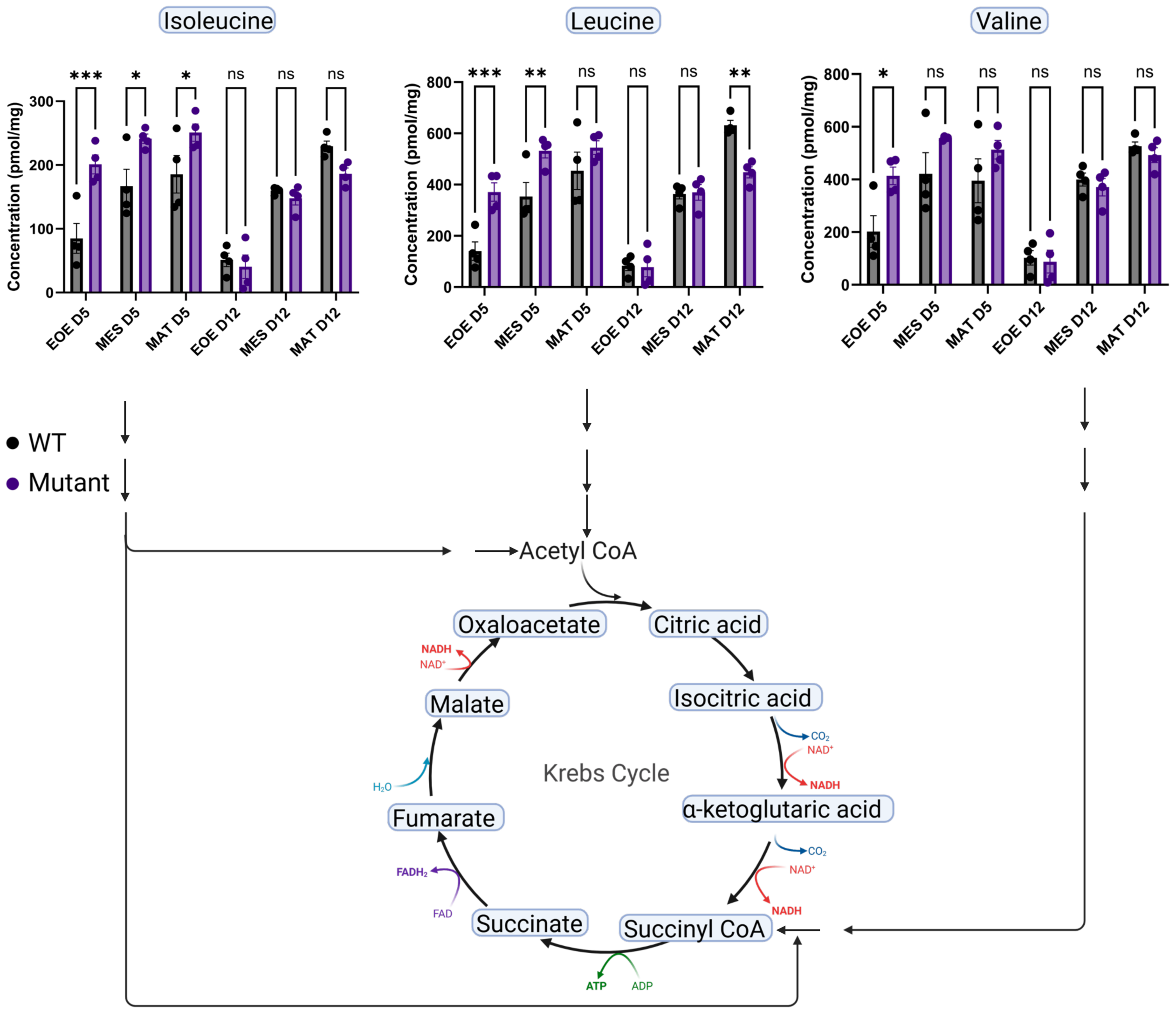

Figure S16. Intracellular concentration of branched-chain amino acids (BCAAs) in *Slc13a5*$^{+/+}$ (WT) and *Slc13a5*$^{R337*/R337*}$ (Mutant) samples vary by developmental day. *Slc13a5*$^{R337*/R337*}$-associated increases in the BCAAs in enamel organ epithelium (EOE) and pulpal mesenchyme cells (MES) significantly decreases as enamel development progressed from day 5 to day 12. No clear trends were observed with the matrix (MAT) sample. Statistics significances were based on ordinary two-way ANOVA and $p<0.05$ were deemed significant. Key: not significant (ns), increasing significance (* $p < 0.05$, ** $p < 0.01$, *** $p < 0.001$, **** $p < 0.0001$). At each time point, there were three samples from each genotype; each sample contained tissues from 12 molars of 3 mice.

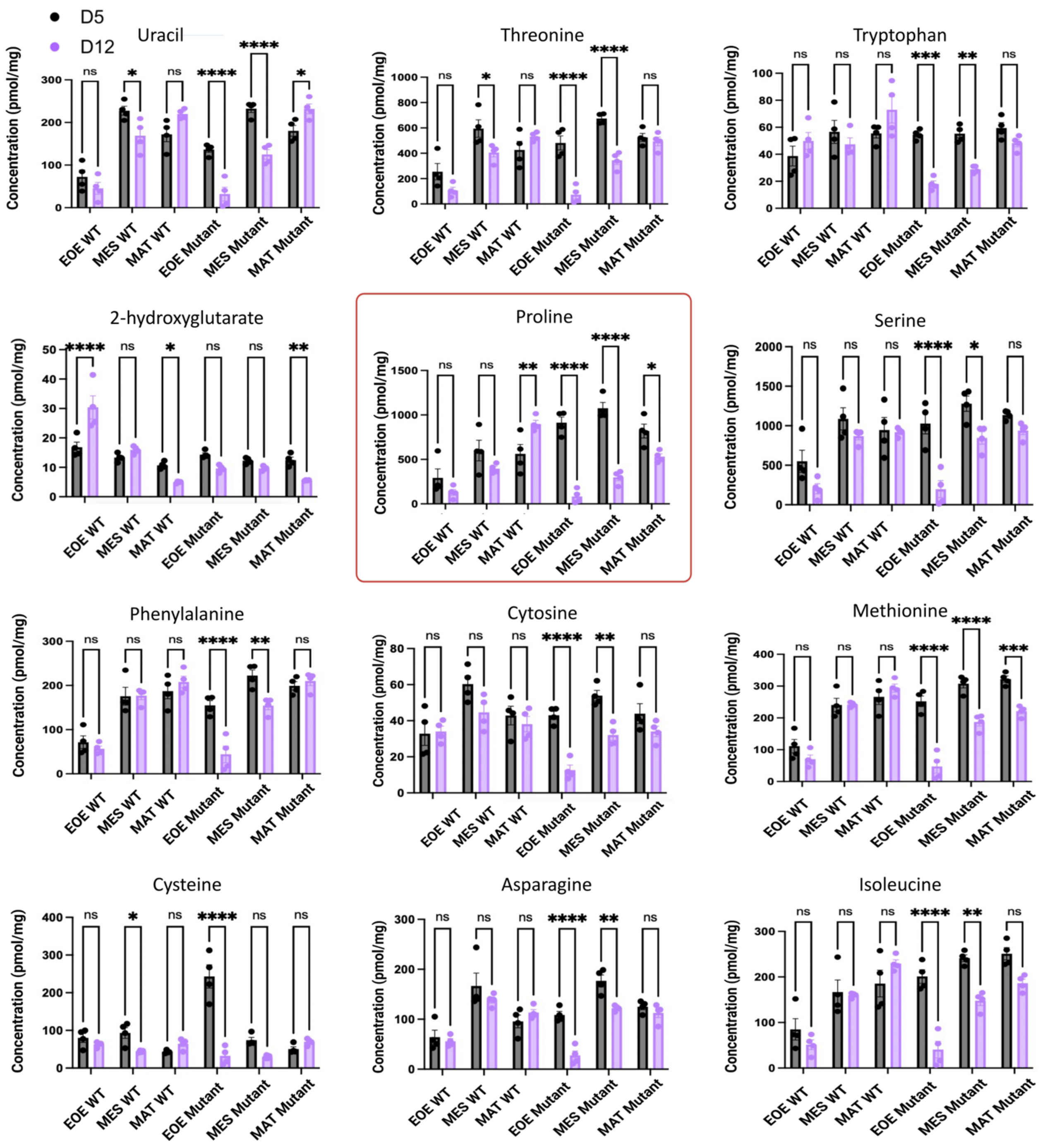

Figure S17. Alterations in non-TCA cycle metabolites between D5 and D12 molar enamel organ epithelium cells (EOE), pulpal mesenchyme cells (MES), and matrix (MAT) from *Slc13a5*$^{+/+}$ (WT) and *Slc13a5*$^{R337*/R337*}$ (Mutant) mice. A decrease in selected amino acids was observed in *Slc13a5*$^{R337*/R337*}$ enamel organ epithelium (EOE) at D12. The decrease in proline was significant in the EOE, mesenchyme (MES), and matrix (MAT) of *Slc13a5*$^{R337*/R337*}$ samples. Statistical significance was determined using ordinary two-way ANOVA, with $p < 0.05$ considered significant. Key: not significant (ns), increasing significance (* $p < 0.05$, ** $p < 0.01$, *** $p < 0.001$, **** $p < 0.0001$). At each time point, there were three samples from each genotype; each sample contained tissues from 12 molars of 3 mice.

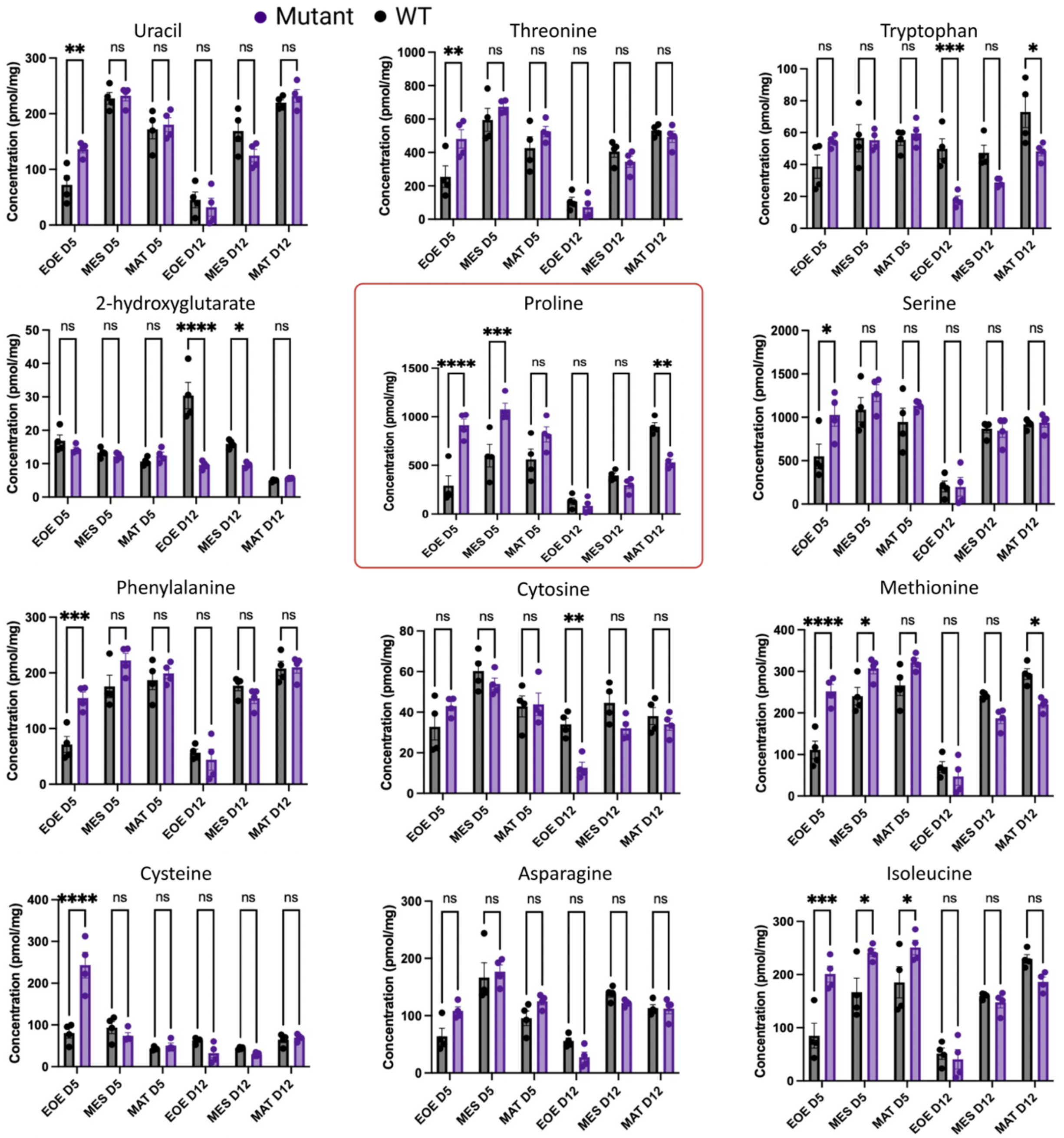

Figure S18. Alterations in non-TCA cycle metabolites between *Slc13a5*$^{+/+}$ (WT) and *Slc13a5*$^{R337*/R337*}$ (Mutant) enamel organ epithelium cells (EOE), pulpal mesenchyme cells (MES), and matrix (MAT) in D5 and D12 molar samples. *Proline content was significantly increased at D5 in the EOE, MES, and MAT regions of Slc13a5*$^{R337*/R337*}$ samples. At D12, proline content did not differ in the EOE and MES, but was decreased in the MAT. Statistical significance was determined using ordinary two-way ANOVA, with $p < 0.05$ considered significant. Key: not significant (ns), increasing significance (* $p < 0.05$, ** $p < 0.01$, *** $p < 0.001$, **** $p < 0.0001$). At each time point, there were three samples from each genotype; each sample contained tissues from 12 molars of 3 mice.

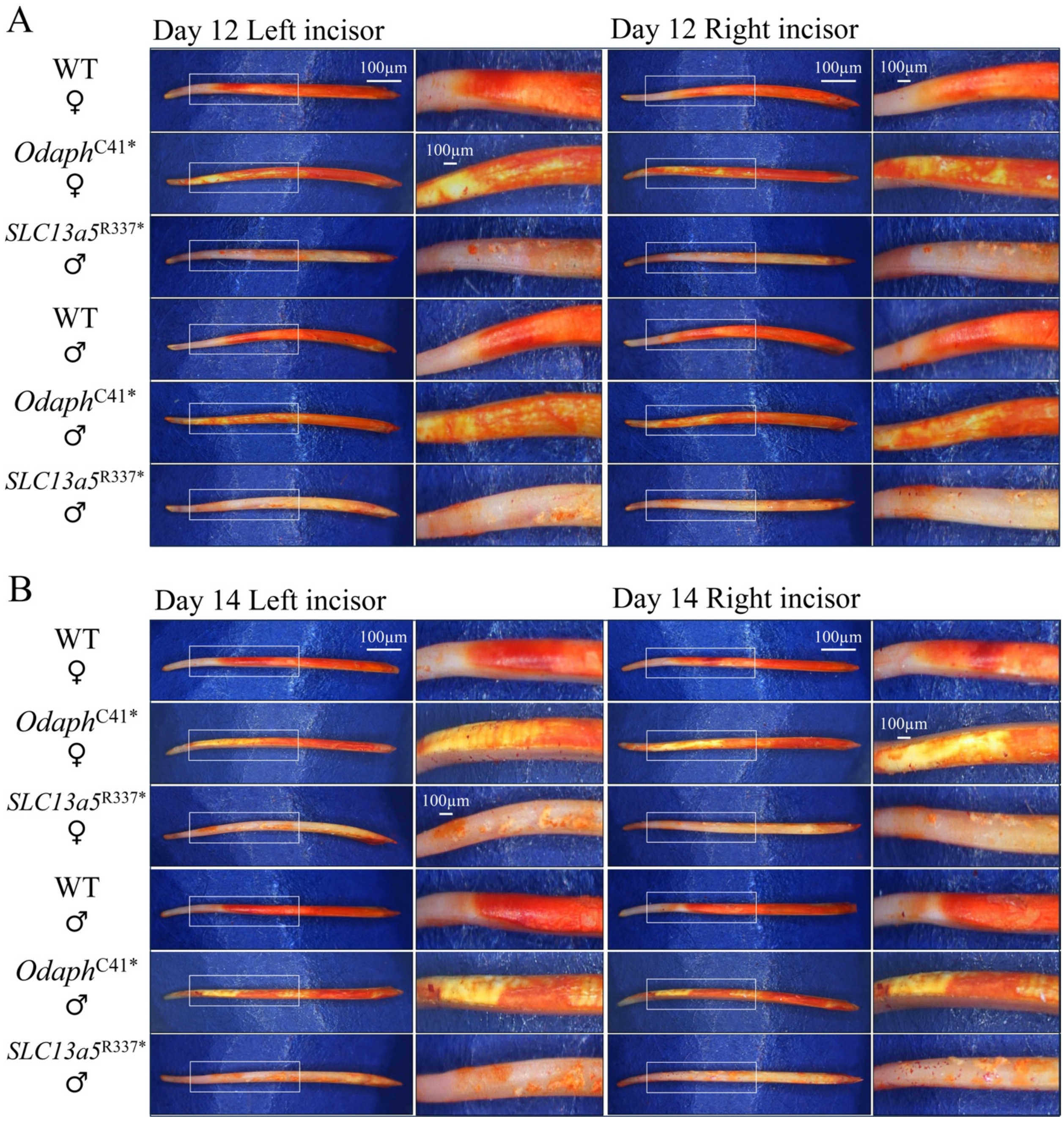

Figure S19. Comparison of pH variation on the labial surface of mandibular incisors from WT, *Slc13a5*$^{R337*/R337*}$, and *Odaph*$^{C41*/C41*}$ male and female mice using methyl red staining. (A) D12 WT incisors from both male and female mice exhibited strong, continuous orange-red staining. In contrast, *Slc13a5*$^{R337*/R337*}$ incisors showed almost no detectable methyl red staining in either sex. *Odaph*$^{C41*/C41*}$ mouse incisors presented irregular, patchy, and discontinuous staining in both sexes. (B) On D14 when enamel maturation is more advanced, these patterns persisted.
